# Supplementary material for: Mental Health Impact of Early Stages of the COVID-19 Pandemic on Individuals with Pre-Existing Mental Disorders: A Systematic Review of Longitudinal Research
Source: Int J Environ Res Public Health. 2023 Jan 4;20(2):948. doi: 10.3390/ijerph20020948 (PMC9858748; doi:10.3390/ijerph20020948)
Supplement: Supplementary file 1 [file ijerph-20-00948-s001.zip › Supplementary material/Supplementary material file 1.docx]

**Supplementary material file 1. Differences between pre-registered protocol and review**

As described in the protocol (see PROSPERO CRD42021251770), we originally planned to summarize the evidence on mental health consequences of the COVID-19 pandemic (question 1) as well as risk and protective factors for mental health (question 2) in individuals with pre-existing mental illness simultaneously. Furthermore, a third research question regarding the evidence on interventions to foster mental health in this population group during the pandemic was addressed based on the same literature searches (see supplementary material file 2 and separate protocol PROSPERO CRD42021251726). Literature searches, study selection, and data extraction were performed simultaneously for the three review questions. The 90 identified cross-sectional studies measuring risk/protective factors were very heterogeneous and there is still limited available evidence on intervention programs (i.e., small number of completed and published studies). Therefore, to provide a first evidence synthesis on the pandemic-related mental health impact in people with pre-existing mental disorders, the current review solely focused on potential mental health effects based on longitudinal research and differentiated by diagnosis.

**Table S1.1. Differences between pre-registered protocol and review**

|  | **Protocol** | **Review** |
| --- | --- | --- |
| Review question | Summarize available literature on mental health impact of COVID-19 pandemic on individuals with pre-existing mental disorders as well as possible (demographic, psychosocial etc.) risk and protective factors for mental health | Summarize available literature on mental health impact of COVID-19 pandemic |
| Types of participants |  | Post-traumatic stress disorder was added as relevant diagnosis |
|  |  | Order of eligible mental disorders was adapted to DSM-5 |
| Types of study to be included | Inclusion of study protocols for observational studies | Only published studies (i.e., no preprints or study protocols) |
| Comparators | No comparator pre-specified | Definition of eligible comparators (see Additional file 3) |
| Outcomes | Secondary outcomes also included routine data, information on mental healthcare supply (e.g., number of [psychiatric] admissions due to a mental disorders or healthcare/support system access and/or utilization), and coping strategies | No routine data, outcomes on healthcare supply/access, or coping strategies as secondary outcomes |
|  | Post-traumatic stress symptoms were named as secondary outcome | Post-traumatic stress symptoms were moved from secondary to primary outcomes |
|  |  | Loneliness was added as secondary outcome |
|  |  | Several potential disorder-specific outcomes were added (e.g., symptoms specific for eating disorder) |

*Note.* DSM-5: Diagnostic and Statistical Manual of Mental Disorders (5^th^ edition).
