# Supplementary material for: Mental Health Impact of Early Stages of the COVID-19 Pandemic on Individuals with Pre-Existing Mental Disorders: A Systematic Review of Longitudinal Research
Source: Int J Environ Res Public Health. 2023 Jan 4;20(2):948. doi: 10.3390/ijerph20020948 (PMC9858748; doi:10.3390/ijerph20020948)
Supplement: Supplementary file 1 [file ijerph-20-00948-s001.zip › Supplementary material/Supplementary material file 10.docx]

**Supplementary material file 10. Vote counting based on effect direction for each diagnostic group (diagnostic groups ordered by DSM-5)**

**Table S10.1. Autism spectrum disorders**

| **Study ID** | **Study design** | **Assessments** | **Outcome measure (direction; range)** | **Effect summary** | **Effect direction** |
| --- | --- | --- | --- | --- | --- |
| **Prior to versus during pandemic** | | | | | |
| Psychological distress | | | | | |
| Goldfarb 2022 [1] | L | P1: 09/2019 - 01/2020  D1: 04/2020 - 05/2020 | GHQ-12 (lower is better; range NR) | P1 vs. D1: *M*±*SD* = 15.6 ± 3.7 vs. 17.2 ± 3.6; Wilcoxon *Z* = -2.67; *p* = 0.004 (Wilcoxon signed rank test) | ↓ |
| **Peri-pandemic changes** | | | | | |
| Anxiety symptoms | | | | | |
| Adams 2021 [2] | L | D1: 11/03/2020 - 20/03/2020 D2: 18/05/2020 - 27/05/2020 | DASS-21 (subscale anxiety; lower is better; 0-42; cut off: 10) | D1 vs. D2: *n* (%) = 115 (41.8%) vs. 104 (37.8%); *p* = 0.131 (McNemar’s);  no significant change in anxiety symptoms from D1 to D2: all *F*s (1, 270) < 1.88, *p*s > 0.05 (Repeated-measures ANOVA) | ↗︎ |
| Lugo-Marin 2021 [3] | L | D1: NR (pre-lockdown)  D2: NR (post-lockdown; 8 weeks after lockdown onset) | SCL-90-R (lower is better; range NR) | D1 vs. D2: *M*±*SD* = 60.7 ± 10.9 vs. 58.2 ± 13.7; Wilcoxon *Z* = -1.26, *p* = 0.21 (Wilcoxon signed rank test) | ↗︎ |
| Depressive symptoms | | | | | |
| Adams 2021 [2] | L | D1: 11/03/2020 - 20/03/2020 D2: 18/05/2020 - 27/05/2020 | DASS-21 (subscale depression; lower is better; 0-42; cut off: 14) | D1 vs. D2: *n* (%) = 103 (37.5%) vs. 101 (36.7%); *p* = 0.758 (McNemar’s);  no significant change in depressive symptoms from D1 to D2: all *F*s (1, 270) < 1.88, *p*s > 0.05 (Repeated-measures ANOVA) | ↗︎ |
| Lugo-Marin 2021 [3] | L | D1: NR (pre-lockdown)  D2: NR (post-lockdown; 8 weeks after lockdown onset) | SCL-90-R (lower is better; range NR) | D1 vs. D2: *M*±*SD* = 62.4 ± 12.1 vs. 57.9 ± 15.1; Wilcoxon *Z* = -2.29, *p* < 0.05 (Wilcoxon signed rank test) | ↑ |
| Psychological distress | | | | | |
| Bal 2021 [4] | L | D1: 30/03/2020 - 10/04/2020  D2: 27/05/2020 - 06/06/2020 | 6 items from COVID-19 set (lower is better; 4 0*no symptoms]* - 16 *[severe]*) | D1 vs. D2: *M*±*SD* = 9.27 ± 3.16 vs. 9.10 ± 3.24; *t*(df) = -1.17 (394), *p* = 0.243; group difference within time point: D2 − D1: Cohen’s *d* = -0.06 (*t*-test) | ↗︎ |
| Brondino 2020 [5]; *psychiatric symptoms and problem behaviors* | L | D1: 19/02/2020  D2: 04/03/2020 | ABC (lower is better; range NR) | D1 vs. D2: *M*±*SD* = 24.83 ± 11.75 vs. 22.33 ± 12.18; *t*(df) = -1.07 (NR), *p* = 0.29 (*t*-test) | ↗︎ |
| Lugo-Marin 2021 [3] | L | D1: NR (pre-lockdown)  D2: NR (post-lockdown; 8 weeks after lockdown onset) | SCL-90-R (GSI; lower is better; range NR) | D1 vs. D2: *M*±*SD* = 64.7 ± 10.6 vs. 58.7 ± 14.9; Wilcoxon *Z* = -3.28; *p* < 0.05 (Wilcoxon signed rank test) | ↑ |
| Stress | | | | | |
| Adams 2021 [2] | L | D1: 11/03/2020 - 20/03/2020 D2: 18/05/2020 - 27/05/2020 | DASS-21 (subscale stress; lower is better; 0-42; cut-off: 19) | D1 vs. D2: n (%) = 87 (31.6%) vs. 84 (30.6%); *p* = 0.674 (McNemar’s);  no significant change in stress from D1 to D2: all *F*s (1, 270) < 1.88, *p*s > 0.05 (Repeated-measures ANOVA) | ↗︎ |
| Lugo-Marin 2021 [3] | L | D1: NR (pre-lockdown)  D2: NR (post-lockdown; 8 weeks after lockdown onset) | Self-developed survey/10-point semantic differential scale (1-4: *low*; 5-6: *no changes*; 7-10: *high*) | D1 vs. D2:  % reporting low stress = 30% vs. 37%; % reporting no changes in stress = 23% vs. 26%; % reporting high stress = 47% vs. 37%; only calculation of frequencies, test and *p* value NR | △_NA_ |

*Note.* Symbols in column ‘Effect direction’ represent the effect direction: ↑(direction 1): clear effect favoring pandemic (vs. pre-pandemic situation) or later peri-pandemic assessment (vs. earlier peri-pandemic assessment); ↗︎ (direction 2): unclear effect potentially favoring pandemic (vs. pre-pandemic situation) or later peri-pandemic assessment (vs. earlier peri-pandemic assessment); ∎ (direction 3): null effect; studies only narratively reporting that ‘no significant difference was observed’, studies reporting an actual null effect based on an effect estimate (e.g., Cohen’s *d* = 0); ↘︎ (direction 4): unclear effect potentially favoring pre-pandemic situation (vs. pandemic) or earlier peri-pandemic assessment (vs. later peri-pandemic assessment); ↓ (direction 5): clear effect favoring pre-pandemic situation (vs. pandemic) or earlier peri-pandemic assessment (vs. later peri-pandemic assessment); △_NA_: based on reported statistical values, probably clear or unclear effect (potentially) favoring the pandemic situation/later peri-pandemic assessment. However, vote counting used in this review was not possible since test and/or p value were not reported to assign to direction 1 or 2; ▽_NA_: based on reported statistical values, probably clear or unclear effect (potentially) favoring the pre-pandemic situation/earlier peri-pandemic assessment. However, vote counting used in this review was not possible since test and/or *p* value were not reported to assign to direction 4 or 5.

*Abbreviations:* ABC: Aberrant Behavior Checklist; ANOVA: analysis of variance; Cohen’s *d*: effect size; D: during COVID-19 assessment (e.g., D1: first during-COVID-19 assessment); DASS-21: Depression Anxiety Stress Scale-21; *df*: degrees of freedom; *F*: *F* value (*F*-test); GHQ-12: General Health Questionnaire–Short Form; GSI: Global Severity Index; ID: identification; L: longitudinal; *M*: mean; *n*: sample size; NR: not reported; P: pre-COVID-19 assessment (e.g., P1: first pre-COVID-19 assessment); *p*: *p* value; SCL-90-R: Symptom Checklist 90 Revised; *SD*: standard deviation; *t*: *t* value (*t*-test); *Z*: *Z* value (Wilcoxon test).

**Table S10.2. Schizophrenia spectrum and other psychotic disorders**

| **Study ID** | **Study design** | **Assessments** | **Outcome measure (direction; range)** | **Effect summary** | **Effect direction** |
| --- | --- | --- | --- | --- | --- |
| **Prior to versus during pandemic** | | | | | |
| Anxiety (symptoms) | | | | | |
| Kott 2020 [6] | R-CS | P1: NR (before the date of the first confirmed COVID-19 case within each country)  D1: NR (after the date of the first confirmed COVID-19 case within each country) | PANSS; anxiety (item G2; lower is better; range NR) | Studies in subjects with acute symptoms (“acute studies”): P1 vs. D1: “ significantly higher severity was observed in the data collected after the reference date for the following items: […] G2 […]”; test NR, *p* NR  Studies in subjects with predominantly negative symptoms: NR | ↓  NR |
| Depressive symptoms | | | | | |
| Pinkham 2020 [7]; *sad/depressed* | L | P1: 04/12/2018 - 04/01/2019 (study 1) and 11/07/2019 - 21/07/2019 (study 2); pre-pandemic symptom severity averaged across all completed surveys  D1: 03/04/2020 - 04/06/2020 | EMA (lower is better; 1-7) | P1 vs. D1: *M*±*SD* = 2.85 ± 1.58 vs. 3.16 ± 2.20; *t*(df) = 1.43 (NR), *p* > 0.05, Cohen's *d* = 0.15 (*t*-test) | ↘︎ |
| Diminished motivation and pleasure | | | | | |
| Strauss 2022 [8] | R-CS | P1: ~08-11/2018 ± 6 months  D1: 07/2020 - 10/2020 | BNSS (diminished MAP, 1 of 2 second-order high-level factors; lower is better; range NR) | P1 vs. D1: *M*±*SD* = 1.23 ± 1.17 vs. 2.11 ± 1.54, *t*(df) = 3.52 (NR), *p* < 0.001 (*t*-test) | ↓ |
| Excitement/energization | | | | | |
| Kott 2020 [6]; *excitement* | R-CS | P1: NR (before the date of the first confirmed COVID-19 case within each country)  D1: NR (after the date of the first confirmed COVID-19 case within each country) | PANSS; excitement (item P4; lower is better; range NR) | Studies in subjects with acute symptoms (“acute studies”): P1 vs. D1: “significantly lower severity was observed for items […], P4 […]”; test NR, *p* NR  Studies in subjects with predominantly negative symptoms: P1 vs. D1: “significantly lower [severity] for items [...], P4, [...]”; test NR, *p* NR | ↑;  ↑ |
| Pinkham 2020 [7]; *energized/excited* | L | P1: 04/12/2018 - 04/01/2019 (study 1) and 11/07/2019 - 21/07/2019 (study 2); pre-pandemic symptom severity averaged across all completed surveys  D1: 03/04/2020 - 04/06/2020 | EMA (lower is better; 1-7) | P1 vs. D1: *M*±*SD* = 3.90 ± 1.59 vs. 4.35 ± 2.13; *t*(df) = 2.42 (NR), *p* < 0.05, Cohen's *d* = 0.25 (*t*-test) | ↓ |
| Hallucinations | | | | | |
| Kott 2020 [6]; *hallucinatory behavior* | R-CS | P1: NR (before the date of the first confirmed COVID-19 case within each country)  D1: NR (after the date of the first confirmed COVID-19 case within each country) | PANSS; hallucinatory behavior (item P3; lower is better; range NR) | Studies in subjects with acute symptoms (“acute studies”): P1 vs. D1: “significantly higher severity was observed in the data collected after the reference date for the following items: […] P3 […]”; test NR, *p* NR  Studies in subjects with predominantly negative symptoms: P1 vs. D1: “significantly lower [severity] for items P3 [...]”; test NR, *p* NR | ↓;  ↑ |
| Pinkham 2020 [7]; *hearing voices* | L | P1: 04/12/2018 - 04/01/2019 (study 1) and 11/07/2019 - 21/07/2019 (study 2); pre-pandemic symptom severity averaged across all completed surveys  D1: 03/04/2020 - 04/06/2020 | EMA (lower is better; 1-7) | P1 vs. D1: *M*±*SD* = 2.47 ± 1.65 vs. 2.42 ± 1.95; *t*(df) = -0.23 (NR), *p* > 0.05, Cohen's *d* = 0.02 (*t*-test) | ↗︎ |
| Psychological distress | | | | | |
| Cordellieri 2021 [9]; *psychiatric symptoms* | L | P1: 11/2019  D1: 04/2020 | BPRS (lower is better; range NR) | P1 vs. D1: *F*(df) = 0.296 (NR), *p* = 0.596, η*_p_*^2^ = 0.022 (ANOVA);  “we did not find statistically significant differences between the BPRS scores” | NA |
| Sleep quantity | | | | | |
| Pinkham 2020 [7] | L | P1: 04/12/2018 - 04/01/2019 (study 1) and 11/07/2019 - 21/07/2019 (study 2); pre-pandemic symptom severity averaged across all completed surveys  D1: 03/04/2020 - 04/06/2020 | EMA (higher is better; in hours/minutes) | P1 vs. D1: *M*±*SD* = 7.35 ± 2.22 vs. 7.01 ± 2.65; *t*(df) = -1.19 (NR), *p* > 0.05, Cohen's *d* = 0.13 (*t*-test) | ↘︎ |
| Substance use | | | | | |
| Cordellieri 2021 [9]; *substance abuse* | L | P1: 11/2019  D1: 04/2020 | K Axis (subscale substance abuse; higher is better; 0 *[very severe compression]* - 100 *[high function]*) | P1 vs. D1: *MD* (*SE*) = -4.861 (0.265), *p* < 0.05; higher mean difference in the T2 compared to the T1 […]. This functional area seemed to improve during the lockdown (higher scores indicate a lower level of criticality in this area).” | ↑ |
| Pinkham 2020 [7);  *substances used (selected from a drop-down list)* | L | P1: 04/12/2018 - 04/01/2019 (study 1) and 11/07/2019 - 21/07/2019 (study 2); pre-pandemic symptom severity averaged across all completed surveys  D1: 03/04/2020 - 04/06/2020 | EMA (drop-down list; lower is better; range NR) | P1 vs. D1: *M*±*SD* = 0.75 ± 0.075 vs. 0.86 ± 0.86; *t*(df) = 1.20 (NR), *p* > 0.05, Cohen's *d* = 0.13 (*t*-test) | ↘︎ |
| Well-being | | | | | |
| Pinkham 2020 [7] | L | P1: 04/12/2018 - 04/01/2019 (study 1) and 11/07/2019 - 21/07/2019 (study 2); pre-pandemic symptom severity averaged across all completed surveys  D1: 03/04/2020 - 04/06/2020 | EMA (higher is better; 1-7) | P1 vs. D1: *M*±*SD* = 4.99 ± 1.36 vs. 5.47 ± 1.70; *t*(df) = 3.34 (NR), *p* < 0.05, Cohen's *d* = 0.35 (*t*-test) | ↑ |
| **Peri-pandemic changes** | | | | | |
| Anxiety symptoms | | | | | |
| Ma 2020^1^ [10] | L | D1: NR (before isolation)  D2: assessments on 10^th^-14^th^ days of isolation (isolation period for 30 participants:  10/01/2020 - 30/04/2020) | HAMA (lower is better; range NR) | D1 vs.D2: *M*±*SD* = 15.47 ± 1.48 vs. 16.97 ± 2.04; *t*(df) = -3.89 (NR), *p* = 0 (*t*-test) | ↓ |
| Wynn 2021 [11] | L | D1: mid 05/2020 - mid 08/2020  D2: mid 08/2020 - mid 10/2020 | GAD-7 (lower is better; range NR) | Psychosis group (PSY): D1 vs. D2: *M*±*SD* = 6.92 ± 6.15 vs. 5.47 ± 5.40; *p* NR (linear mixed-effects models); “Both […] and PSY showed […], followed by a significant decrease from initial to follow-up” | ↑ |
| Depressive symptoms | | | | | |
| Ma 2020 [10] | L | D1: NR (before isolation)  D2: assessments on 10^th^-14^th^ days of isolation (isolation period for 30 participants:  10/01/2020 - 30/04/2020) | HAMD (lower is better; range NR) | D1 vs. D2: *M*±*SD* = 19.97 ± 1.47 vs. 20.83 ± 3.82; *t*(df) = -1.14 (NR), *p* = 0.27 (*t*-test) | ↘︎ |
| Wynn 2021 [11] | L | D1: mid 05/2020 - mid 08/2020  D2: mid 08/2020 - mid 10/2020 | PHQ-9 (lower is better; range NR) | Psychosis group (PSY): D1 vs. D2: *M*±*SD* = 8.61 ± 6.32 vs. 6.60 ± 5.40; *p* NR (linear mixed-effects models); “Both […] and PSY showed […], followed by a significant decrease from initial to follow-up” | ↑ |
| Diminished motivation and pleasure | | | | | |
| Wynn 2021 [11] | L | D1: mid 05/2020 - mid 08/2020  D2: mid 08/2020 - mid 10/2020 | MAP-SR (lower is better; range NR) | Psychosis group (PSY): D1 vs. D2: *M*±*SD* = 34.83 ± 14.51 vs. 36.48 ± 13.31; *p* NR (linear mixed-effects models); “For Motivation and Pleasure […], the main effect of Time and the Group × Time interaction were not significant.” | ↘︎ |
| Loneliness | | | | | |
| Wynn 2021 [11] | L | D1: mid 05/2020 - mid 08/2020  D2: mid 08/2020 - mid 10/2020 | ULS (lower is better; range NR) | Psychosis group (PSY): D1 vs. D2: *M*±*SD* = 24.52 ± 14.99 vs. 20.52 ± 15.48; *p* < 0.05 (linear mixed-effects models); “For loneliness, there were significant main effects of Time and Group (Fig. 1D). […] Follow-up tests for the Time main effect revealed significant differences between each pair of time points (*p*’s < 0.05), with loneliness highest at the initial assessment” | ↑ |
| Psychological distress | | | | | |
| Daly 2021 [12] | L | D1: 10-18/03/2020  D2: 01-14/04/2020  D3: 15-28/04/2020  D4: 29/04 - 12/05/2020  D5: 13-26/05/2020  D6: 27/05-09/06/2020  D7:10-23/06/2020  D8: 24/06-20/07/2020 | PHQ-4 (lower is better; 0-12) | Increase D1 to D2: β = -0.38, 95% CI -0.97 to 0.20, *p* > 0.05 (linear regression model)^2^;  Decrease D2 to D8: β = 0.02, 95% CI -0.53 to 0.57, *p* > 0.05 (linear regression model)^2^ | ↗︎;  ↗︎ |
| Ma 2020 [10]; *severity of psychiatric symptoms* | L | D1: NR (before isolation)  D2: assessments on 10^th^-14^th^ days of isolation (isolation period for 30 participants:  10/01/2020 - 30/04/2020) | PANSS (total score; lower is better; range NR) | D1 vs. D2: *M*±*SD* = 90.23 ± 7.75 vs. 88.27 ± 10.52; *t*(df) = 0.94 (NR), *p* = 0.36 (*t*-test) | ↗︎ |
| Ma 2021 [13]; *psychopathology* | L | D1: 01/2020 (patients were uninfected)  D2: NR (within 3 days of diagnosis with COVID-19 after patient was transferred to isolation ward)  D3: NR (after patients were cured; before they were transferred out of isolation ward; transfer of last cured patient on 30/03/2020) | PANSS (total score; lower is better; range NR) | D1 vs. D2: Figure 1a in Ma 2021; PANSS score = ~75 vs. ~65; *M*±*SD* and *t*(df) NR, *p* = 0.225 (analysis NR);  D2 vs. D3: Figure 1a in Ma 2021; PANSS score = ~65 vs. ~77.5; *M*±*SD* and t(df) NR, *p* = 0.399 (analysis NR) | ↗︎;  ↘︎ |
| Sleep quality | | | | | |
| Ma 2020 [10] | L | D1: NR (before isolation)  D2: assessments on 10^th^-14^th^ days of isolation (isolation period for 30 participants:  10/01/2020 - 30/04/2020) | PSQI (total score; lower is better; range NR) | D1 vs. D2: *M*±*SD* = 5.57 ± 2.85 vs. 7.50 ± 3.50; *t*(df) = -3.00 (NR), *p* = 0 (*t*-test) | ↓ |
| Stress | | | | | |
| Ma 2020 [10] | L | D1: NR (before isolation)  D2: assessments on 10^th^-14^th^ days of isolation (isolation period for 30 participants:  10/01/2020 - 30/04/2020) | CPSS (lower is better; range NR) | D1 vs. D2: *M*±*SD* = 24.97 ± 5.39 vs. 27.17 ± 5.25; *t*(df) = -3.39 (NR), *p* = 0 (*t*-test) | ↓ |
| Ma 2021 [13] | L | D1: 01/2020 (patients were uninfected)  D2: NR (within 3 days of diagnosis with COVID-19 after patient was transferred to isolation ward)  D3: NR (after patients were cured; before they were transferred out of isolation ward; transfer of last cured patient on 30/03/2020) | CPSS (lower is better; range NR) | D1 vs. D2: Figure 1c in Ma 2021; CPSS score = ~16 vs. ~27, *M*±*SD* and *t*(df) NR, *p* < 0.001 (analysis NR);  D2 vs. D3: Figure 1c in Ma 2021; CPSS score = ~27 vs. ~17, *M*±*SD* and *t*(df) NR *p* < 0.001 (analysis NR) | ↓;  ↑ |
| Substance use | | | | | |
| Wynn 2021 [11]; *alcohol use, cannabis use (frequent use)* | L | D1: mid 05/2020 - mid 08/2020  D2: mid 08/2020 - mid 10/2020 | ASI (% participants reporting use in past 30 days for *none*, *moderate/occasional [1-8 days]* and *severe/frequent [9+ days]*; lower is better) | Alcohol use:  Psychosis group (PSY): D1 vs. D2: frequent: 10.4% vs. 9.6%; *p* NR (linear mixed-effects models); “For alcohol use, there were no significant main effects of Time or Group, […].”  Cannabis use:  Psychosis group (PSY): D1 vs. D2: frequent: 10.4% vs. 9.6%; *p* NR (linear mixed-effects models); “For cannabis use, there was […] a significant main effect of Time […]. However, there was a significant Group × Time interaction […]. The significant interaction was driven by the RHV reporting a significant increase in cannabis use at Initial relative to pre-COVID; there were no significant changes in the PSY or CTL groups.” | ↗︎    ↗︎ |

*Note.* Symbols in column ‘Effect direction’ represent the effect direction: ↑(direction 1): clear effect favoring pandemic (vs. pre-pandemic situation) or later peri-pandemic assessment (vs. earlier peri-pandemic assessment); ↗︎ (direction 2): unclear effect potentially favoring pandemic (vs. pre-pandemic situation) or later peri-pandemic assessment (vs. earlier peri-pandemic assessment); ∎ (direction 3): null effect; studies only narratively reporting that ‘no significant difference was observed’, studies reporting an actual null effect based on an effect estimate (e.g., Cohen’s *d* = 0); ↘︎ (direction 4): unclear effect potentially favoring pre-pandemic situation (vs. pandemic) or earlier peri-pandemic assessment (vs. later peri-pandemic assessment); ↓ (direction 5): clear effect favoring pre-pandemic situation (vs. pandemic) or earlier peri-pandemic assessment (vs. later peri-pandemic assessment); △_NA_: based on reported statistical values, probably clear or unclear effect (potentially) favoring the pandemic situation/later peri-pandemic assessment. However, vote counting used in this review was not possible since test and/or p value were not reported to assign to direction 1 or 2; ▽_NA_: based on reported statistical values, probably clear or unclear effect (potentially) favoring the pre-pandemic situation/earlier peri-pandemic assessment. However, vote counting used in this review was not possible since test and/or *p* value were not reported to assign to direction 4 or 5.

^1^ Case-control study with longitudinal comparison of the differences before and after isolation among the isolation group.

^2^ Adjusted for participant age, sex, race/ethnicity, and household income as covariates; for ‘increase’, a multivariate linear regression was conducted with ‘increase in PHQ-4 in the period D1 to D2 (10-18/03/2020 to 01-14/04/2020)’ as dependent variable, for ‘decrease’, a multivariate linear regression was conducted with ‘decrease in PHQ-4 in the period D2 to D8 (01-14/04/2020 to 24/06-20/07/2020)’ as dependent variable.

*Abbreviations:* ~: approximately; ANOVA: analysis of variance; ASI: Addiction Severity Index; β: regression coefficient; BNSS: Brief Negative Symptom Scale; BPRS: Brief Psychiatric Rating Scale; Cohen’s *d*: effect size; CI: confidence interval; CPSS Chinese Perceived Stress Scale; D: during COVID-19 assessment (e.g., D1: first during-COVID-19 assessment); *df*: degrees of freedom; EMA: Ecological Momentary Assessment; *F*: *F* value (*F*-test); GAD-7: General Anxiety Disorder; η*_p_*^2^: partial eta^2^; HAMA: Hamilton Anxiety Scale; HAM-D: Hamilton Rating Scale for Depression; ID: identification; K Axis: Kennedy Axis V; L: longitudinal; *M*: mean; MAP-SR: Motivation and Pleasure Scale – Self-Report; *MD*: mean difference; NR: not reported; P: pre-COVID-19 assessment (e.g., P1: first pre-COVID-19 assessment); *p*: *p* value; PANSS: Positive and Negative Symptom Scale; PHQ-4/-9: Patient Health Questionnaire-4/-9; PSQI Pittsburgh Sleep Quality Index; R-CS: repeated cross-sectional; *SD*: standard deviation; *SE*: standard error; *t*: *t* value (*t*-test); ULS: Revised UCLA Loneliness Scale.

**Table S10.3. Bipolar disorders**

| **Study ID** | **Study design** | **Assessments** | **Outcome measure (direction; range)** | **Effect summary** | **Effect direction** |
| --- | --- | --- | --- | --- | --- |
| **Prior to versus during pandemic** | | | | | |
| Anxiety symptoms | | | | | |
| Orhan 2021 [14] | L | P1: 2017 - 2018  D1: 04/2020 | BAI (lower is better; 0-63) | P1 vs. D1: *Mdn* (*IQR*) = 7 (15.3) vs. *Mdn* (*IQR*) = 5.5 (7), Wilcoxon *Z* = NR;  *p* = 0.02 (Wilcoxon signed-rank test) | ↑ |
| Yocum 2021 (mixed) [15] | L | For comparison pre-pandemic vs. peri-pandemic:  P1: 15/03/2015-2019 to 30/05/2015-2019  D1: 15/03/2020 - 30/05/2020 | GAD-7 (lower is better; range NR) | P1 vs. D1: linear effect in the log scale = 0.0365, *p* = 0.3246 (generalized estimating equations);  P1 vs. D1: *M* = 6.95 vs. 7.41, *p* = 0.32 | ↘︎ |
| Depressive symptoms | | | | | |
| Orhan 2021 [14] | L | P1: 2017 - 2018  D1: 04/2020 | CES-D (lower is better; range; cut-off: ≥16: clinically relevant depression) | P1 vs. D1: *Mdn* (*IQR*) = 13 (18) vs. 8 (13.8), Wilcoxon *Z* = NR; *p* < 0.01 (Wilcoxon signed-rank test) | ↑ |
| Yocum 2021  (mixed) [15] | L | For comparison pre-pandemic vs. peri-pandemic:  P1: 15/03/2015-2019 to 30/05/2015-2019  D1: 15/03/2020 - 30/05/2020 | PHQ-9 (lower is better; range NR) | P1 vs. D1: linear effect in the log scale = 0.0489, *p* = 0.1512 (generalized estimating equations);  P1 vs. D1: *M* = 7.28 vs. 7.92, *p* = 0.15 (test NR) | ↘︎ |
| (Hypo-)maniac symptoms | | | | | |
| Orhan 2021 [14] | L | P1: 2017 - 2018  D1: 04/2020 | YMRS (lower is better; range: NR; ≥12: clinically relevant (hypo)mania | P1 vs. D1: *Mdn* (*IQR*) = 2 (4) vs. 0 (3), Wilcoxon *Z* = NR; *p* < 0.01 (Wilcoxon signed-rank test) | ↑ |
| Loneliness | | | | | |
| Orhan 2021 [14] | L | P1: 2017 - 2018  D1: 04/2020 | Loneliness Scale (lower is better; 0-11) | P1 vs. D1: *Mdn* (*IQR*) = 3 (6) vs. 3 (4), Wilcoxon *Z* = NR; *p* = 0.06 (Wilcoxon signed-rank test) | ∎ |
| Sleep quality | | | | | |
| Yocum 2021  (mixed) [15]; *bad sleep quality* | L | For comparison pre-pandemic vs. peri-pandemic:  P1: 15/03/2015-2019 to 30/05/2015-2019  D1: 15/03/2020 - 30/05/2020 | PSQI (bad quality) | P1 vs. D1: *OR* = 0.9738, *p* = 0.8725 (generalized estimating equations) | ↗︎ |
| **Peri-pandemic changes** | | | | | |
| Anxiety symptoms | | | | | |
| Yocum 2021  (mixed) [15] | L | For peri-pandemic trajectories:  D1: 30/04/2020  D2: 14/05/2020  D3: 28/05/2020 | GAD-7 (lower is better; range NR) | D1 vs. D2: linear effect in the log scale = -0.0306, *p* = 0.5256 (generalized estimating equations)  D1 vs. D3: linear effect in the log scale = -0.0480, *p* = 0.4261 (generalized estimating equations) | ↗︎;  ↗︎ |
| Circadian rhythms | | | | | |
| Carta 2021 [16] | L | D1: 04/2020  D2: 06/2020 | BRIAN scale, total score (lower is better [indicates higher level of adjustment to biological rhythms]; 18-72) | D1 vs. D2:  Cagliari: *M*±*SD* = 44.1 ± 10.83 vs. 45.07 ± 12.00; Tunis: *M*±*SD* = 34.8 ± 8.05 vs. 35.4 ± 8.15  Repeated-measures ANOVA for D1 vs. D2 (for both Cagliari and Tunis): *F*(1,68) = 0.341, *p* = 0.561; circadian rhythms did not differ significantly between time points | ↘︎ |
| Depressive symptoms | | | | | |
| Carta 2021 [16] | L | D1: 04/2020  D2: 06/2020 | HAM-D (lower is better; range NR; cut-off for depressive episode: 14) | Cagliari: *n* (%) with HAM-D >14: D1 vs. D2 = 18 (45%) vs. 14 (35%), χ^2^ = 0.833; *p* = 0.361;  Tunis: *n* (%) with HAM-D >14: D1 vs. D2 = 0 (0%) vs. 0 (0%); test NR, *p* NR | ↗︎;  ∎_NA_ |
| Yocum 2021  (mixed) [15] | L | For peri-pandemic trajectories:  D1: 30/04/2020  D2: 14/05/2020  D3: 28/05/2020 | PHQ-9 (lower is better; 0-27) | D1 vs. D2: linear effect in the log scale = -0.0231, *p* = 0.5602 (generalized estimating equations)  D1 vs. D3: linear effect in the log scale = -0.1000, *p* = 0.0752 (generalized estimating equations) | ↗︎;  ↗︎ |
| Psychological distress | | | | | |
| Daly 2021 [12] | L | D1: 10-18/03/2020  D2: 01-14/04/2020  D3: 15-28/04/2020  D4: 29/04 - 12/05/2020  D5: 13-26/05/2020  D6: 27/05-09/06/2020  D7:10-23/06/2020  D8: 24/06-20/07/2020 | PHQ-4 (lower is better; 0-12) | Increase D1 to D2: β = 0.20, 95% CI -0.12 to 0.52, *p* > 0.05 (linear regression model)^1^;  Decrease D2 to D8: β = -0.01, 95% CI -0.27 to 0.25, *p* > 0.05 (linear regression model)^1^ | ↘︎;  ↘︎ |
| Sleep quality | | | | | |
| Yocum 2021  (mixed) [15]; *bad sleep quality* | L | For peri-pandemic trajectories:  D1: 30/04/2020  D2: 14/05/2020  D3: 28/05/2020 | PSQI (bad quality) | D1 vs. D2: *OR* = 0.9428, *p* = 0.6739  D1 vs. D3: *OR* = 0.9026, *p* = 0.5855 | ↗︎;  ↗︎ |
| Stress | | | | | |
| Yocum 2021  (mixed) [15]; *experiencing pandemic-related stress* | L | For peri-pandemic trajectories:  D1: 30/04/2020  D2: 14/05/2020  D3: 28/05/2020 | CIS (yes) | D1 vs. D2: *OR* = 1.0825, *p* = 0.5168  D1 vs. D3: *OR* = 0.8766, *p* = 0.3169 | ↘︎;  ↗︎ |

*Note.* Symbols in column ‘Effect direction’ represent the effect direction: ↑(direction 1): clear effect favoring pandemic (vs. pre-pandemic situation) or later peri-pandemic assessment (vs. earlier peri-pandemic assessment); ↗︎ (direction 2): unclear effect potentially favoring pandemic (vs. pre-pandemic situation) or later peri-pandemic assessment (vs. earlier peri-pandemic assessment); ∎ (direction 3): null effect; studies only narratively reporting that ‘no significant difference was observed’, studies reporting an actual null effect based on an effect estimate (e.g., Cohen’s *d* = 0); ↘︎ (direction 4): unclear effect potentially favoring pre-pandemic situation (vs. pandemic) or earlier peri-pandemic assessment (vs. later peri-pandemic assessment); ↓ (direction 5): clear effect favoring pre-pandemic situation (vs. pandemic) or earlier peri-pandemic assessment (vs. later peri-pandemic assessment); △_NA_: based on reported statistical values, probably clear or unclear effect (potentially) favoring the pandemic situation/later peri-pandemic assessment. However, vote counting used in this review was not possible since test and/or p value were not reported to assign to direction 1 or 2; ▽_NA_: based on reported statistical values, probably clear or unclear effect (potentially) favoring the pre-pandemic situation/earlier peri-pandemic assessment. However, vote counting used in this review was not possible since test and/or *p* value were not reported to assign to direction 4 or 5.

^1^ Adjusted for participant age, sex, race/ethnicity, and household income as covariates; for ‘increase’, a multivariate linear regression was conducted with ‘increase in PHQ-4 in the period D1 to D2 (10-18/03/2020 to 01-14/04/2020)’ as dependent variable, for ‘decrease’, a multivariate linear regression was conducted with ‘decrease in PHQ-4 in the period D2 to D8 (01-14/04/2020 to 24/06-20/07/2020)’ as dependent variable.

*Abbreviations:* ANOVA: analysis of variance; β: regression coefficient; BAI: Beck Anxiety Inventory; BRIAN: Biological Rhythms Interview of Assessment in Neuropsychiatry; CES‐D: Center for Epidemiologic Studies Depression Scale; CI: confidence interval; CIS: Coronavirus Impact Scale; D: during COVID-19 assessment (e.g., D1: first during-COVID-19 assessment); *F*: *F* value (*F*-test); GAD-7: General Anxiety Disorder; HAM-D: Hamilton Rating Scale for Depression; ID: identification; *IQR*: interquartile range; L: longitudinal; *M*: mean; *Mdn*: Median; *n*: sample size; NR: not reported; *OR*: Odds Ratio; P: pre-COVID-19 assessment (e.g., P1: first pre-COVID-19 assessment); *p*: *p* value; PHQ-4/-9: Patient Health Questionnaire-4/-9; PSQI Pittsburgh Sleep Quality Index; *S*D: standard deviation; χ^2^: χ^2^value (χ^2^ test); YMRS: Young Mania Rating Scale; *Z*: *Z* value (Wilcoxon test).

**Table S10.4. Depressive disorders**

| **Study ID** | **Study design** | **Assessments** | **Outcome measure (direction; range)** | **Effect summary** | **Effect direction** |
| --- | --- | --- | --- | --- | --- |
| **Prior to versus during pandemic** | | | | | |
| Anxiety symptoms | | | | | |
| Hamm 2020 [17] | L | P1: ~04/2019 ± 9 months (anxiety symptoms), ~05/2019 ± 8 months (depressive symptoms)  P2: ~12/2019 ± 5 months (depressive symptoms)  D1: 01/04/2020 - 23/04/2020 | PROMIS-anxiety (lower is better; range NR) | P1 vs. D1: *M*±*SD* = 63.5 ± 6.1 vs. 57.5 ± 8.0; *MD* (*SD*) = −5.8 (9.1); *t*(df) = 5.2 (64); *p* < 0.001 (*t*-test) | ↑ |
| Pan 2021 [18] | L | P1: 2006 - 2016  D1: 01/04/2020 - 13/05/2020 | BAI (lower is better; range NR) | Major depressive disorder: P1 vs. D1: *M* = 10.16 vs. 10.09; Adjusted change (*SE*) = 0.03 (0.63); *p* = 0.97 (mixed models)^1^  Dysthymic disorder: P1 vs. D1: *M* = 12.75 vs. 12.04; Adjusted change (*SE*) = -0.64 (0.70); *p* = 0.36 (mixed models)^1^ | ↘︎;  ↗︎ |
| Depressive symptoms | | | | | |
| Hamm 2020 [17] | L | P1: ~04/2019 ± 9 months (anxiety symptoms), ~05/2019 ± 8 months (depressive symptoms)  P2: ~12/2019 ± 5 months (depressive symptoms)  D1: 01/04/2020 - 23/04/2020 | PHQ-9 (lower is better; 0-27) | P1 vs. D1: *M*±*SD* = 14.7 ± 4.2 vs. 8.7 ± 5.0; *MD* (*SD*) = -6.0 (4.9); *t*(df) = 10.4 (71); *p* < 0.001 (*t*-test)  P2 vs. D1: *M*±*SD* = 8.8 ± 5.4 vs. 8.7 ± 5.0; *MD* (*SD*) = -0.1 (4.9); *t*(df) = 0.2 (71), *p* = 0.8 (*t*-test) | ↑;  ↗︎ |
| Pan 2021 [18] | L | P1: 2006 - 2016  D1: 01/04/2020 - 13/05/2020 | QIDS (lower is better; range NR) | Major depressive disorder: P1 vs. D1: *M* = 6.94 vs. 7.02; Adjusted change (*SE*) = 0.02 (0.32); *p* = 0.96 (mixed models)^1^  Dysthymic disorder: P1 vs. D1: *M* = 9.01 vs. 8.53; Adjusted change (*SE*) = -0.77 (0.35); *p* = 0.029 (mixed models)^1^ | ↘︎;  ↑ |
| Loneliness | | | | | |
| Pan 2021 [18] | L | P1: 2006 - 2016  D1: 01/04/2020 - 13/05/2020 | DJGLS (lower is better; range NR) | Major depressive disorder: P1 vs. D1: *M* = 2.41 vs. 2.56; Adjusted change (*SE*) = 0.03 (0.17); *p* = 0.88 (mixed models)^1^  Dysthymic disorder: P1 vs. D1: *M* = 3.08 vs. 2.90; Adjusted change (*SE*) = -0.43 (0.18); *p* = 0.017 (mixed models)^1^ | ↘︎;  ↑ |
| **Peri-pandemic changes** | | | | | |
| Psychological distress | | | | | |
| Daly 2021 [12] | L | D1: 10-18/03/2020  D2: 01-14/04/2020  D3: 15-28/04/2020  D4: 29/04 - 12/05/2020  D5: 13-26/05/2020  D6: 27/05-09/06/2020  D7:10-23/06/2020  D8: 24/06-20/07/2020 | PHQ-4 (lower is better; 0-12) | Increase D1 to D2: β = 0.22, 95% CI 0.12 to 0.33, *p* < 0.001 (linear regression model)^2^;  Decrease D2 to D8: β = 0.24, 95% CI 0.15 to 0.34, *p* < 0.001 (linear regression model)^2^ | ↓;  ↑ |

*Note.* Symbols in column ‘Effect direction’ represent the effect direction: ↑(direction 1): clear effect favoring pandemic (vs. pre-pandemic situation) or later peri-pandemic assessment (vs. earlier peri-pandemic assessment); ↗︎ (direction 2): unclear effect potentially favoring pandemic (vs. pre-pandemic situation) or later peri-pandemic assessment (vs. earlier peri-pandemic assessment); ∎ (direction 3): null effect; studies only narratively reporting that ‘no significant difference was observed’, studies reporting an actual null effect based on an effect estimate (e.g., Cohen’s *d* = 0); ↘︎ (direction 4): unclear effect potentially favoring pre-pandemic situation (vs. pandemic) or earlier peri-pandemic assessment (vs. later peri-pandemic assessment); ↓ (direction 5): clear effect favoring pre-pandemic situation (vs. pandemic) or earlier peri-pandemic assessment (vs. later peri-pandemic assessment); △_NA_: based on reported statistical values, probably clear or unclear effect (potentially) favoring the pandemic situation/later peri-pandemic assessment. However, vote counting used in this review was not possible since test and/or p value were not reported to assign to direction 1 or 2; ▽_NA_: based on reported statistical values, probably clear or unclear effect (potentially) favoring the pre-pandemic situation/earlier peri-pandemic assessment. However, vote counting used in this review was not possible since test and/or *p* value were not reported to assign to direction 4 or 5.

^1^ Adjusted for age, sex, education, living situation, and date of response.

^2^ Adjusted for participant age, sex, race/ethnicity, and household income as covariates; for ‘increase’, a multivariate linear regression was conducted with ‘increase in PHQ-4 in the period D1 to D2 (10-18/03/2020 to 01-14/04/2020)’ as dependent variable, for ‘decrease’, a multivariate linear regression was conducted with ‘decrease in PHQ-4 in the period D2 to D8 (01-14/04/2020 to 24/06-20/07/2020)’ as dependent variable.

*Abbreviations:* ~: approximately; β: regression coefficient; BAI: Beck Anxiety Inventory; CI: confidence interval; D: during COVID-19 assessment (e.g., D1: first during-COVID-19 assessment); *df*: degrees of freedom; DJGLS: De Jong Gierveld Loneliness Scale; ID: identification; L: longitudinal; *M*: mean; NR: not reported; P: pre-COVID-19 assessment (e.g., P1: first pre-COVID-19 assessment); *p*: *p* value; PHQ-4/-9: Patient Health Questionnaire-4/-9; PROMIS-anxiety: Patient Reported Outcomes Measurement Information System-anxiety scale; QIDS: Quick Inventory of Depressive Symptoms; *SD*: standard deviation, *SE*: standard error; *t*: *t* value (*t*-test).

**Table S10.5. Anxiety disorders**

| **Study ID** | **Study design** | **Assessments** | **Outcome measure (direction; range)** | **Effect summary** | **Effect direction** |
| --- | --- | --- | --- | --- | --- |
| **Prior to versus. during pandemic** | | | | | |
| Anxiety symptoms | | | | | |
| Pan 2021 [18] | L | P1: 2006 - 2016  D1: 01/04/2020 - 13/05/2020 | BAI (lower is better; range NR) | Panic disorder: P1 vs. D1: *M* = 12.75 vs. 12.26; Adjusted change (*SE*) = -0.57 (0.69); *p* = 0.41 (mixed models)^1^  Generalized anxiety disorder: P1 vs. D1: *M* = 12.51 vs. 12.02; Adjusted change (*SE*) = -0.39 (0.64); *p* = 0.54 (mixed models)^1^  Agoraphobia: P1 vs. D1: *M* = 13.15 vs. 12.82; Adjusted change (*SE*) = 0.14 (0.72); *p* = 0.85 (mixed models)^1^  Social anxiety disorder: P1 vs. D1: *M* = 12.26 vs. 11.84; Adjusted change (*SE*) = -0.40 (0.62); *p* = 0.52 (mixed models)^1^ | ↗︎;  ↗︎;  ↘︎;  ↗︎ |
| Depressive symptoms | | | | | |
| Pan 2021 [18] | L | P1: 2006 - 2016  D1: 01/04/2020 - 13/05/2020 | QIDS (lower is better; range NR) | Panic disorder: P1 vs. D1: *M* = 7.45 vs. 7.38; Adjusted change (*SE*) = -0.04 (0.35); *p* = 0.91 (mixed models)^1^  Generalized anxiety disorder: P1 vs. D1: *M* = 8.09 vs. 7.91; Adjusted change (*SE*) = -0.21 (0.33); *p* = 0.53 (mixed models)^1^  Agoraphobia: P1 vs. D1: *M* = 7.74 vs. 7.57; Adjusted change (*SE*) = -0.16 (0.37); *p* = 0.66 (mixed models)^1^  Social anxiety disorder: P1 vs. D1: *M* = 7.80 vs. 7.61; Adjusted change (*SE*) = -0.35 (0.32); *p* = 0.28 (mixed models)^1^ | ↗︎;  ↗︎;  ↗︎;  ↗︎ |
| Loneliness | | | | | |
| Pan 2021 [18] | L | P1: 2006 - 2016  D1: 01/04/2020 - 13/05/2020 | DJGLS (lower is better; range NR) | Panic disorder: P1 vs. D1: *M* = 2.58 vs. 2.67; Adjusted change (*SE*) = 0.04 (0.18); *p* = 0.81 (mixed models)^1^  Generalized anxiety disorder: P1 vs. D1: *M* = 2.75 vs. 2.78; Adjusted change (*SE*) = -0.05 (0.17); *p* = 0.78 (mixed models)^1^  Agoraphobia: P1 vs. D1: *M* = 2.76 vs. 2.75; Adjusted change (*SE*) = -0.14 (0.19); *p* = 0.47 (mixed models)^1^  Social anxiety disorder: P1 vs. D1: *M* = 2.88 vs. 2.84; Adjusted change (*SE*) = -0.26 (0.16); *p* = 0.11 (mixed models)^1^ | ↘︎;  ↗︎;  ↗︎;  ↗︎ |
| **Peri-pandemic changes** | | | | | |
| Psychological distress | | | | | |
| Daly 2021 [12] | L | D1: 10-18/03/2020  D2: 01-14/04/2020  D3: 15-28/04/2020  D4: 29/04 - 12/05/2020  D5: 13-26/05/2020  D6: 27/05-09/06/2020  D7:10-23/06/2020  D8: 24/06-20/07/2020 | PHQ-4 (lower is better; 0-12) | Increase D1 to D2: β = 0.26, 95% CI 0.13 to 0.39, *p* < 0.001 (linear regression model)^2^;  Decrease D2 to D8: β = 0.25, 95% CI 0.15 to 0.36, *p* < 0.001 (linear regression model)^2^ | ↓;  ↑ |

*Note.* Symbols in column ‘Effect direction’ represent the effect direction: ↑(direction 1): clear effect favoring pandemic (vs. pre-pandemic situation) or later peri-pandemic assessment (vs. earlier peri-pandemic assessment); ↗︎ (direction 2): unclear effect potentially favoring pandemic (vs. pre-pandemic situation) or later peri-pandemic assessment (vs. earlier peri-pandemic assessment); ∎ (direction 3): null effect; studies only narratively reporting that ‘no significant difference was observed’, studies reporting an actual null effect based on an effect estimate (e.g., Cohen’s *d* = 0); ↘︎ (direction 4): unclear effect potentially favoring pre-pandemic situation (vs. pandemic) or earlier peri-pandemic assessment (vs. later peri-pandemic assessment); ↓ (direction 5): clear effect favoring pre-pandemic situation (vs. pandemic) or earlier peri-pandemic assessment (vs. later peri-pandemic assessment); △_NA_: based on reported statistical values, probably clear or unclear effect (potentially) favoring the pandemic situation/later peri-pandemic assessment. However, vote counting used in this review was not possible since test and/or p value were not reported to assign to direction 1 or 2; ▽_NA_: based on reported statistical values, probably clear or unclear effect (potentially) favoring the pre-pandemic situation/earlier peri-pandemic assessment. However, vote counting used in this review was not possible since test and/or *p* value were not reported to assign to direction 4 or 5.

^1^ Adjusted for age, sex, education, living situation, and date of response.

^2^ Adjusted for participant age, sex, race/ethnicity, and household income as covariates; for ‘increase’, a multivariate linear regression was conducted with ‘increase in PHQ-4 in the period D1 to D2 (10-18/03/2020 to 01-14/04/2020)’ as dependent variable, for ‘decrease’, a multivariate linear regression was conducted with ‘decrease in PHQ-4 in the period D2 to D8 (01-14/04/2020 to 24/06-20/07/2020)’ as dependent variable.

*Abbreviations:* β: regression coefficient; BAI: Beck Anxiety Inventory; CI: confidence interval; D: during COVID-19 assessment (e.g., D1: first during-COVID-19 assessment); DJGLS: De Jong Gierveld Loneliness Scale; ID: identification; L: longitudinal; *M*: mean; NR: not reported; P: pre-COVID-19 assessment (e.g., P1: first pre-COVID-19 assessment); *p*: *p* value; PHQ-4: Patient Health Questionnaire-4; QIDS: Quick Inventory of Depressive Symptoms; *SE*: standard error.

**Table S10.6. Obsessive-compulsive disorders**

| **Study ID** | **Study design** | **Assessments** | **Outcome measure (direction; range)** | **Effect summary** | **Effect direction** |
| --- | --- | --- | --- | --- | --- |
| **Prior to versus during pandemic** | | | | | |
| Anxiety symptoms | | | | | |
| Pan 2021 [18] | L | P1: 2006 - 2016  D1: 01/04/2020 - 13/05/2020 | BAI (lower is better; range NR) | P1 vs. D1: *M* = 14.03 vs. 14.92; Adjusted change (*SE*) = 0.68 (0.97); *p* = 0.49 (mixed models)^1^ | ↘︎ |
| Loneliness | | | | | |
| Pan 2021 [18] | L | P1: 2006 - 2016  D1: 01/04/2020 - 13/05/2020 | DJGLS (lower is better; range NR) | P1 vs. D1: *M* = 2.50 vs. 2.70; Adjusted change (*SE*) = -0.13 (0.24); *p* = 0.57 (mixed models)^1^ | ↗︎ |
| OCD severity | | | | | |
| Chakraborty 2020 [19] | L | P1: NR (pre-pandemic scores; last recorded Y-BOCS severity score noted from the case register)  D1: 23/04/2020 - 22/05/2020 | Y-BOCS (total/severity score; lower is better; range NR) | P1 vs. D1: Y-BOCS score % increase: Most of the patients scored same or decreased in Y-BOCS (41 [48.8%]) or showed a < 5% increase (33 [39.3%]) as recorded in last visit; narrative results: “we did not find any increase in obsessive and compulsive symptoms […]” | NA |
| Khosravani 2021 [20] | L | P1: NR (before outbreak of COVID-19)  D1: 05/2020 - 07/2020 | Y-BOCS (total score; lower is better; range NR) | P1 vs. D1: *M*±*SD* = 19.6 ± 9.1 vs. 28.7 ± 8.2; *t*(df) = 12.7 (NR); *p* < 0.001 (*t*-test) | ↓ |
| Matsunaga 2020 [21] | L | P1: NR (before December 2019)  D1: 07/04/2020-02/05/2020 | Y-BOCS (total score; lower is better; range NR) | Fully remitted group (Y-BOCS total score < 8): P1 vs. D1: *M*±*SD* = 5.5 ± 1.4 vs. 5.7 ± 1.5, test NR, *p* NR; narrative results: “little acute impact on the phenomenological features and severity of OCD”  Partially remitted group (9 < Y-BOCS total score < 15): P1 vs. D1: *M*±*SD* = 12.2 ± 2.2 vs.13.0 ± 2.3; test NR, *p* NR; narrative results: “little acute impact on the phenomenological features and severity of OCD” | ▽_NA_  ▽_NA_ |
| Sharma 2021 [22] | R-CS | Historical control cohort:  P1: NR, baseline assessment (first visit to OCD clinic)  P2: 01/10/2018 – 28/02/2019 (Fu visit in clinic; 1 year prior to FU visit of pandemic cohort)  P3: 04/2019 - 05/2019 (2^nd^ FU visit in clinic; 1 year prior to 2^nd^ FU visit of pandemic cohort)  Pandemic cohort:  P1: NR, baseline assessment (first visit to OCD clinic)  P2: 01/10/2019 - 29/02/2020 (FU visit in clinic before pandemic)  D1: 26/04/2020 - 12/05/2020 (telephonic FU during pandemic) | Y-BOCS (total score; lower is better; range NR; remission: Y-BOCS total score <12) | [Baseline: Historical control cohort (P1) vs. pandemic cohort (P1): *M*±*SD* = 25.86 ± 7.06 vs. 25.54 ± 6.69; *t*(df) = -0.411 (NR), *p* = 0.682 (*t*-test)];  [Last FU: Historical control cohort (P2) vs. pandemic cohort (P2): *M*±*SD* = 16.08 ± 10.50 vs. 14.37 ± 10.70; *t*(df) = -1.706 (NR), *p* = 0.089 (*t*-test)];  Current: Historical control cohort (P3) vs. pandemic cohort (D1): *M*±*SD* = 14.26 ± 10.59 vs. 12.80 ± 9.95; *F*-test or *t*-test NR, *p* NR  overall: χ^2^ likelihood ratio test of interaction term (time x cohort) in linear mixed-effects model: χ^2^ = 2.73; *p* = 0.255 | △_NA_ |
| **Peri-pandemic changes** | | | | | |
| OCD severity | | | | | |
| Davide 2020 [23] | L | D1: 01/2020 - 02/2020  D2: 16-17/04/2020 | Y-BOCS-SC (lower is better; 0-40) | D1 vs. D2: *M*±*SD* = 15.97 ± 8.028 vs. 20.467 ± 8.4475; *t*(df) = -4.35 (29); *p* < 0.001 (*t*-test) | ↓ |
| Psychological distress | | | | | |
| Daly 2021 [12] | L | D1: 10-18/03/2020  D2: 01-14/04/2020  D3: 15-28/04/2020  D4: 29/04 - 12/05/2020  D5: 13-26/05/2020  D6: 27/05-09/06/2020  D7:10-23/06/2020  D8: 24/06-20/07/2020 | PHQ-4 (lower is better; 0-12) | Increase D1 to D2: β = 0.02, 95% CI -0.22 to 0.26, *p* > 0.05 (linear regression model)^2^;  Decrease D2 to D8: β = 0.34, 95% CI 0.08 to 0.60, *p* < 0.05 (linear regression model)^2^ | ↘︎;  ↑ |

*Note.* Symbols in column ‘Effect direction’ represent the effect direction: ↑(direction 1): clear effect favoring pandemic (vs. pre-pandemic situation) or later peri-pandemic assessment (vs. earlier peri-pandemic assessment); ↗︎ (direction 2): unclear effect potentially favoring pandemic (vs. pre-pandemic situation) or later peri-pandemic assessment (vs. earlier peri-pandemic assessment); ∎ (direction 3): null effect; studies only narratively reporting that ‘no significant difference was observed’, studies reporting an actual null effect based on an effect estimate (e.g., Cohen’s *d* = 0); ↘︎ (direction 4): unclear effect potentially favoring pre-pandemic situation (vs. pandemic) or earlier peri-pandemic assessment (vs. later peri-pandemic assessment); ↓ (direction 5): clear effect favoring pre-pandemic situation (vs. pandemic) or earlier peri-pandemic assessment (vs. later peri-pandemic assessment); △_NA_: based on reported statistical values, probably clear or unclear effect (potentially) favoring the pandemic situation/later peri-pandemic assessment. However, vote counting used in this review was not possible since test and/or p value were not reported to assign to direction 1 or 2; ▽_NA_: based on reported statistical values, probably clear or unclear effect (potentially) favoring the pre-pandemic situation/earlier peri-pandemic assessment. However, vote counting used in this review was not possible since test and/or *p* value were not reported to assign to direction 4 or 5.

^1^ Adjusted for age, sex, education, living situation, and date of response.

^2^ Adjusted for participant age, sex, race/ethnicity, and household income as covariates; for ‘increase’, a multivariate linear regression was conducted with ‘increase in PHQ-4 in the period D1 to D2 (10-18/03/2020 to 01-14/04/2020)’ as dependent variable, for ‘decrease’, a multivariate linear regression was conducted with ‘decrease in PHQ-4 in the period D2 to D8 (01-14/04/2020 to 24/06-20/07/2020)’ as dependent variable.

*Abbreviations:* β: regression coefficient; BAI: Beck Anxiety Inventory; CI: confidence interval; D: during COVID-19 assessment (e.g., D1: first during-COVID-19 assessment); *df*: degrees of freedom; DJGLS: De Jong Gierveld Loneliness Scale; FU: follow-up; ID: identification; L: longitudinal; *M*: mean; NR: not reported; OCD: obsessive-compulsive disorder; P: pre-COVID-19 assessment (e.g., P1: first pre-COVID-19 assessment); *p*: *p* value; PHQ-4: Patient Health Questionnaire-4; R-CS: repeated cross-sectional; *SD*: standard deviation, *SE*: standard error; *t*: *t* value (*t-*test); χ^2^ : χ^2^ value; Y-BOCS(-SC): Yale Brown Obsessive Compulsive Scale (-Symptom Checklist).

**Table S10.7. Post-traumatic stress disorders**

| **Study ID** | **Study design** | **Assessments** | **Outcome measure (direction; range)** | **Effect summary** | **Effect direction** |
| --- | --- | --- | --- | --- | --- |
| **Prior to versus during pandemic** | | | | | |
| Depressive symptoms | | | | | |
| Rutherford 2021 [24] | L | P1: before 13/03/2020 ^1^  D1: 01/04/2020 - 08/05/2020 | HRSD (lower is better; range NR) | P1 vs. D1: *M*±*SD* = 15.21 ± 1.09 vs. 17.08 ± 1.12, *t*(df) = 1.35 (69), *p* = 0.181 (*t*-test) | ↘︎ |
| Post-traumatic stress symptoms | | | | | |
| Rutherford 2021 [24] | L | P1: before 13/03/2020 ^1^  D1: 01/04/2020 - 08/05/2020 | PCL-5 (lower is better; range NR) | P1 vs. D1: *M*±*SD* = 42.27 ± 1.65 vs. 35.2 ± 2.02; *t*(df) = -3.53 (69), *p* = 0.0008 (*t*-test) | ↑ |
| **Peri-pandemic changes** | | | | | |
| Psychological distress | | | | | |
| Daly 2021 [12] | L | D1: 10-18/03/2020  D2: 01-14/04/2020  D3: 15-28/04/2020  D4: 29/04 - 12/05/2020  D5: 13-26/05/2020  D6: 27/05-09/06/2020  D7:10-23/06/2020  D8: 24/06-20/07/2020 | PHQ-4 (lower is better; 0-12) | Increase D1 to D2: β = 0.02, 95% CI -0.17 to 0.21, *p* > 0.05 (linear regression model)^2^;  Decrease D2 to D8: β = 0.18, 95% CI -0.01 to 0.37, *p* > 0.05 (linear regression model)^2^ | ↘︎;  ↗︎ |

*Note.* Symbols in column ‘Effect direction’ represent the effect direction: ↑(direction 1): clear effect favoring pandemic (vs. pre-pandemic situation) or later peri-pandemic assessment (vs. earlier peri-pandemic assessment); ↗︎(direction 2): unclear effect potentially favoring pandemic (vs. pre-pandemic situation) or later peri-pandemic assessment (vs. earlier peri-pandemic assessment); ∎ (direction 3): null effect; studies only narratively reporting that ‘no significant difference was observed’, studies reporting an actual null effect based on an effect estimate (e.g., Cohen’s *d* = 0); ↘︎ (direction 4): unclear effect potentially favoring pre-pandemic situation (vs. pandemic) or earlier peri-pandemic assessment (vs. later peri-pandemic assessment); ↓ (direction 5): clear effect favoring pre-pandemic situation (vs. pandemic) or earlier peri-pandemic assessment (vs. later peri-pandemic assessment); △_NA_: based on reported statistical values, probably clear or unclear effect (potentially) favoring the pandemic situation/later peri-pandemic assessment. However, vote counting used in this review was not possible since test and/or p value were not reported to assign to direction 1 or 2; ▽_NA_: based on reported statistical values, probably clear or unclear effect (potentially) favoring the pre-pandemic situation/earlier peri-pandemic assessment. However, vote counting used in this review was not possible since test and/or *p* value were not reported to assign to direction 4 or 5.

^1^ Baseline assessment up until 13/03/2020 (reported as pre-COVID-19 pandemic by the authors) however, first COVID-19 case in the USA registered on 20/01/2020 according to WHO data.

^2^ Adjusted for participant age, sex, race/ethnicity, and household income as covariates; for ‘increase’, a multivariate linear regression was conducted with ‘increase in PHQ-4 in the period D1 to D2 (10-18/03/2020 to 01-14/04/2020)’ as dependent variable, for ‘decrease’, a multivariate linear regression was conducted with ‘decrease in PHQ-4 in the period D2 to D8 (01-14/04/2020 to 24/06-20/07/2020)’ as dependent variable.

*Abbreviations:* β: regression coefficient; CI: confidence interval; D: during COVID-19 assessment (e.g., D1: first during-COVID-19 assessment); *df*: degrees of freedom; HRSD: Hamilton Rating Scale for Depression; ID: identification; L: longitudinal; *M*: mean; NR: not reported; P: pre-COVID-19 assessment (e.g., P1: first pre-COVID-19 assessment); *p*: *p v*alue; PCL-5: Post-traumatic Stress Disorder Checklist; PHQ-4: Patient Health Questionnaire-4; *SD*: standard deviation, *t*: *t* value (*t*-test).

**Table S10.8. Eating disorders**

| **Study ID** | **Study design** | **Assessments** | **Outcome measure (direction; range)** | **Effect summary** | **Effect direction** |
| --- | --- | --- | --- | --- | --- |
| **Prior to versus during pandemic** | | | | | |
| Depressive symptoms | | | | | |
| Giel 2021 [25] | L | P1: NR (entering IMPULS trial; IMPULS trial carried out between 03/2015 - 09/2017)  P2: NR (end of treatment/trial)  D1: 05/2020 - 07/2020 | BDI-II (lower is better; range NR) | P1 vs. D1: *M*±*SD* = 13.5 ± 9.4 vs. 14.1 ± 11.5, Wald χ^2^ = 1.92, *p* = 0.166 (Wald test)  P2 vs. D1: *M*±*SD* = 11.6 ± 9.2 vs. 14.1 ± 11.5, Wald χ^2^ = 5.41, *p* = 0.020 (Wald test) | ↘︎;  ↓ |
| Eating disorder specific psychopathology | | | | | |
| Castellini 2020 [26] | L | P1: 01/2019 - 09/2019  P2: 11/2019 - 01/2020 (pre-lockdown)  D1: 22/04/2020 - 03/05/2020 (in-lockdown) | EDE-Q (total score; lower is better; range NR) | [P1 vs. P2: Figure 1 in Castellini 2020; BN: reduction, *p* < 0.05, Cohen’s *d* = 0.52; AN: reduction, *p* < 0.05, Cohen’s *d* = 0.39 (mixed modelling)]  P1 vs. D1: Figure 1 in Castellini 2020; BN: reduction, *p* < 0.05, Cohen’s *d* = NR; AN: reduction, *p* < 0.05, Cohen’s *d* = NR (mixed modelling)  P2 vs. D1: Figure 1 in Castellini 2020; BN: reduction, *p* > 0.05, Cohen’s *d* = NR; AN: reduction; *p* < 0.05, Cohen’s *d* = 0.26 (mixed modelling);  [overall (BN + AN): *M*±*SD* = 2.72 ± 1.72 vs. 2.33 ± 1.63; *p* > 0.05, Cohen’s *d* = NR] | ↑; ↑  ↗︎; ↑ |
| Giel 2021 [25] | L | P1: NR (entering IMPULS trial; IMPULS trial carried out between 03/2015 - 09/2017)  P2: NR (end of treatment/trial)  D1: 05/2020 - 07/2020 | EDE-Q (total score; lower is better; range NR) | P1 vs. D1: *M*±*SD* = 2.5 ± 0.8 vs. 3.1 ± 0.7; Wald χ^2^ = 7.51, *p* = 0.006 (Wald test)  P2 vs. D1: *M*±*SD* = 2.0 ± 0.8 vs. 3.1 ± 0.7, Wald χ^2^ = 35.52, *p* <0.001 (Wald test) | ↓;  ↓ |
| Machado 2020 [27] | L | P1: NR (last available evaluation before COVID-19 lockdown period)  D1: 30/04/2020 - 15/05/2020 | EDE-Q (total score; lower is better; range NR) | P1 vs. D1: *M*±*SD* = 2.92 ± 1.57 vs. 2.93 ± 1.58, test NR, *p* NR, narratively reported: not significant after Bonferroni correction for multiple comparisons | ↘︎ |
| Loss of control over eating behavior/binge-eating episodes | | | | | |
| Castellini 2020 [26]; *objective binge eating (monthly)* | L | P1: 01/2019 - 09/2019  P2: 11/2019 - 01/2020 (pre-lockdown)  D1: 22/04/2020 - 03/05/2020 (in-lockdown) | NR (lower is better; probably frequency) | [P1 vs. P2: Figure 1 in Castellini 2020; BN: reduction, *p* < 0.05, Cohen’s *d* = 1.06; AN: reduction, *p* < 0.05, Cohen’s *d* = 0.41 (mixed modelling)]  P1 vs. D1: Figure 1 in Castellini 2020; BN: reduction, *p* < 0.05, Cohen’s *d* = NR; AN: reduction, *p* > 0.05, Cohen’s *d* = NR (mixed modelling)  P2 vs. D1: Figure 1 in Castellini 2020; BN: increase, *p* < 0.05, Cohen’s *d* = 0.32; AN: increase, *p* > 0.05, Cohen’s *d* = NR (mixed modelling);  [overall (BN + AN): *M*±*SD* = 2.33 ± 2.51 vs. 4.32 ± 7.17, *p* < 0.05, Cohen’s *d* = NR] | ↑; ↗︎  ↓; ↘︎ |
| Psychological distress | | | | | |
| Castellini 2020 [26]; *general psychopathology* | L | P1: 01/2019 - 09/2019  P2: 11/2019 - 01/2020 (pre-lockdown)  D1: 22/04/2020 - 03/05/2020 (in-lockdown) | BSI (GSI; lower is better; range NR) | [P1 vs. P2: Figure 1 in Castellini 2020; BN: reduction, *p* < 0.05, Cohen’s *d* = 0.78; AN: reduction, *p* < 0.05, Cohen’s *d* = 0.66 (mixed modelling)]  P1 vs. D1: Figure 1 in Castellini 2020; BN: reduction, *p* < 0.05, Cohen’s *d* = NR; AN: reduction, *p* < 0.05, Cohen’s *d* = NR (mixed modelling)  P2 vs. D1: Figure 1 in Castellini 2020; BN: reduction, *p* > 0.05, Cohen’s *d* = NR; AN: reduction; *p* > 0.05, Cohen’s *d* = NR (mixed modelling);  [overall (BN + AN): *M*±*SD* = 1.11 ± 0.72 vs. 0.99 ± 2.33, *p* > 0.05, Cohen’s *d* = NR] | ↑; ↑  ↗︎; ↗︎ |
| Giel 2021 [25]; *general psychopathology (comorbid mental health diagnosis)* | L | P1: NR (entering IMPULS trial; IMPULS trial carried out between 03/2015 - 09/2017)  P2: NR (end of treatment/trial)  D1: 05/2020 - 07/2020 | SCID expert rating (prevalence as dichotomous outcome; lower is better) | P1 vs. D1: Prevalence = 23.5% vs. 8.80%, Wald χ^2^ = 2.71, *p* = 0.100 (Wald test)  P2 vs D1: Prevalence = 41.2%, vs. 8.80%, Wald χ^2^ = 11.85, *p* = 0.002 (Wald test) | ↗︎;  ↑ |
| **Peri-pandemic changes** | | | | | |
| Anxiety symptoms | | | | | |
| Nisticò 2021 [28] | L | D1: 25/04/2020 - 28/04/2020  D2: 25/06/2020 - 28/06/2020 | DASS-21 (subscale anxiety; lower is better; range NR) | D1 vs. D2: *M*±*SD* = 11.4 ± 5.1 vs. 4.7 ± 4.3, *F*(df) = 2.783 (NR), *p* = 0.103, η*_p_*^2^ = 0.067 (Repeated-measures ANOVA) | ↗︎ |
| Depressive symptoms | | | | | |
| Nisticò 2021 [28] | L | D1: 25/04/2020 - 28/04/2020  D2: 25/06/2020 - 28/06/2020 | DASS-21 (subscale depression; lower is better; range NR) | D1 vs. D2: *M*±*SD* = 9.6 ± 5.9 vs. 8.5 ± 5.9, *F*(df) = 1.762 (NR), *p* = 0.192, η*_p_*^2^ = 0.043 (Repeated-measures ANOVA) | ↗︎ |
| Loss of control over eating behavior/binge-eating episodes | | | | | |
| Leenaerts 2021 [29]; *binge eating frequency* | L | D1: 10/01/2020 - 14/03/2020  D2: 19/03/2020 - 09/05/2020 | Self-developed item (0 *[no binge]* vs. 1 *[binge]*; lower is better) | D1 vs. D2 (before vs. during lockdown measures): β = 0.11, *SE* = 0.19, *p* = 0.548 (mixed effects model) | ↘︎ |
| Nisticò 2021 [28] | L | D1: 25/04/2020 - 28/04/2020  D2: 25/06/2020 - 28/06/2020 | EDE-Q single item (lower is better; range NR) | D1 vs. D2: *M*±*SD* = 5.7 ± 1.9 vs. 4.5 ± 2.2, *F(*df) = 6.465 (NR), *p* = 0.015, η*_p_*^2^ = 0.142 (Repeated-measures ANOVA) | ↑ |
| Psychological distress | | | | | |
| Daly 2021 [12] | L | D1: 10-18/03/2020  D2: 01-14/04/2020  D3: 15-28/04/2020  D4: 29/04 - 12/05/2020  D5: 13-26/05/2020  D6: 27/05-09/06/2020  D7:10-23/06/2020  D8: 24/06-20/07/2020 | PHQ-4 (lower is better; 0-12) | Increase D1 to D2: β = -0.06, 95% CI -0.44 to 0.31, *p* > 0.05 (linear regression model)^1^;  Decrease D2 to D8: β = 0.08, 95% CI -0.25 to 0.42, *p* > 0.05 (linear regression model)^1^ | ↗︎;  ↗︎ |
| Nisticò 2021 [28] | L | D1: 25/04/2020 - 28/04/2020  D2: 25/06/2020 - 28/06/2020 | DASS-21 (total score; lower is better; range NR) | D1 vs. D2: *M*±*SD* = 26.7 ± 13.9 vs. 23.3 ± 13.9, *F*(df) = 3.331 (NR), *p* = 0.076, η*_p_*^2^ = 0.079 (Repeated-measures ANOVA) | ↗︎ |
| Stress | | | | | |
| Nisticò 2021 [28] | L | D1: 25/04/2020 - 28/04/2020  D2: 25/06/2020 - 28/06/2020 | PSS (lower is better; range NR) | D1 vs. D2: *M*±*SD* = 24.7 ± 8.4 vs. 24.3 ± 8.4, *F*(df) = 0.134 (NR), *p* = 0.717, η*_p_*^2^ = 0.003 (Repeated-measures ANOVA) | ↗︎ |
| Well-being | | | | | |
| Nisticò 2021 [28] | L | D1: 25/04/2020 - 28/04/2020  D2: 25/06/2020 - 28/06/2020 | EDE-Q single item (higher is better; range NR) | D1 vs. D2: *M*±*SD* = 3.53 ± 2.1 vs. 4.2 ± 1.9, *F*(df) = 4.067 (NR), *p* = 0.051, η*_p_*^2^ = 0.094 (Repeated-measures ANOVA) | ↗︎ |

*Note.* Symbols in column ‘Effect direction’ represent the effect direction: ↑(direction 1): clear effect favoring pandemic (vs. pre-pandemic situation) or later peri-pandemic assessment (vs. earlier peri-pandemic assessment); ↗︎ (direction 2): unclear effect potentially favoring pandemic (vs. pre-pandemic situation) or later peri-pandemic assessment (vs. earlier peri-pandemic assessment); ∎ (direction 3): null effect; studies only narratively reporting that ‘no significant difference was observed’, studies reporting an actual null effect based on an effect estimate (e.g., Cohen’s *d* = 0); ↘︎ (direction 4): unclear effect potentially favoring pre-pandemic situation (vs. pandemic) or earlier peri-pandemic assessment (vs. later peri-pandemic assessment); ↓ (direction 5): clear effect favoring pre-pandemic situation (vs. pandemic) or earlier peri-pandemic assessment (vs. later peri-pandemic assessment); △_NA_: based on reported statistical values, probably clear or unclear effect (potentially) favoring the pandemic situation/later peri-pandemic assessment. However, vote counting used in this review was not possible since test and/or p value were not reported to assign to direction 1 or 2; ▽_NA_: based on reported statistical values, probably clear or unclear effect (potentially) favoring the pre-pandemic situation/earlier peri-pandemic assessment. However, vote counting used in this review was not possible since test and/or *p* value were not reported to assign to direction 4 or 5.

^1^ Adjusted for participant age, sex, race/ethnicity, and household income as covariates; for ‘increase’, a multivariate linear regression was conducted with ‘increase in PHQ-4 in the period D1 to D2 (10-18/03/2020 to 01-14/04/2020)’ as dependent variable, for ‘decrease’, a multivariate linear regression was conducted with ‘decrease in PHQ-4 in the period D2 to D8 (01-14/04/2020 to 24/06-20/07/2020)’ as dependent variable.

*Abbreviations:* AN: Anorexia nervosa; ANOVA: analysis of variance; β: regression coefficient; BDI‐II: Beck Depression Inventory‐II; BN: Bulimia Nervosa; BSI: Brief Symptom Inventory; Cohen’s *d*: effect size; CI: confidence interval; D: during COVID-19 assessment (e.g., D1: first during-COVID-19 assessment); DASS-21: Depression Anxiety and Stress Scale-21; *df*: degrees of freedom; EDE-Q: Eating Disorder Examination Questionnaire; *F*: *F* value (*F*-test); GSI: Global Severity Index; η*_p_*^2^: partial eta^2^; ID: identification; L: longitudinal; *M*: mean; NR: not reported; P: pre-COVID-19 assessment (e.g., P1: first pre-COVID-19 assessment); *p*: *p* value; PHQ-4: Patient Health Questionnaire-4; PSS: Perceived Stress Scale; SCID: Structured Clinical Interview for DSM-5; *SD*: standard deviation, *SE*: standard error; χ^2^ : Wald χ^2^ (Wald test).

**Table S10.9. Substance-related disorders**

| **Study ID** | **Study design** | **Assessments** | **Outcome measure (direction; range)** | **Effect summary** | **Effect direction** |
| --- | --- | --- | --- | --- | --- |
| **Prior to versus during pandemic** | | | | | |
| Alcohol use | | | | | |
| Liu 2021 [30] | L | P1: 10/2019 - 12/2019  D1: 02/2020 - 04/2020 (outbreak)  D2: 05/2020- 06/2020 (post-pandemic) | Self-developed questionnaire (in ml; lower is better) | P1 vs. D1: Figure 3 in Liu 2021; alcohol use in ml = ~120 ml vs. ~210 ml, test NR, *p* < 0.01;  P1 vs. D2: Figure 3 in Liu 2021; alcohol use in ml = ~120 ml vs. ~140 ml; test NR, *p* NR  [overall: significant time effect on alcohol use: *F*(df) = 54.45 (NR), *p* < 0.01 (Repeated-measures ANOVA)] | ↓;  ▽_NA_ |
| Amphetamine use | | | | | |
| Liu 2021 [30] | L | P1: 10/2019 - 12/2019  D1: 02/2020 - 04/2020 (outbreak)  D2: 05/2020- 06/2020 (post-pandemic) | Urine drug test board (positive rate %; lower is better) | P1 vs. D1: Figure 3 in Liu 2021; Positive rate (%) = ~12.5% vs. ~4%, test NR, *p* NR  P1 vs. D2: Figure 3 in Liu 2021; Positive rate (%) = ~12.5% vs. ~22.5%, test NR, *p* NR  [overall: significant time effect on amphetamine use: χ^2^ = 3.60, *p* = 0.03 (χ^2^ test)] | △_NA_;  ▽_NA_ |
| Anxiety symptoms | | | | | |
| Liu 2021 [30] | L | P1: 10/2019 - 12/2019  D1: 02/2020 - 04/2020 (outbreak)  D2: 05/2020- 06/2020 (post-pandemic) | HAMA (lower is better; range NR) | P1 vs. D1: Figure 1 in Liu 2021; HAMA score = ~5 vs. ~8, test NR, *p* < 0.01;  P1 vs. D2: Figure 1 in Liu 2021; HAMA score = ~5 vs. ~4, test NR, *p* NR  [overall: significant time effect on HAMA score: *F*(df) = 53.43 (NR), *p* < 0.01 (Repeated-measures ANOVA)] | ↓;  △_NA_ |
| Depressive symptoms | | | | | |
| Liu 2021 [30] | L | P1: 10/2019 - 12/2019  D1: 02/2020 - 04/2020 (outbreak)  D2: 05/2020- 06/2020 (post-pandemic) | HAMD (lower is better; range NR) | P1 vs. D1: Figure 1 in Liu 2021; HAMD score = ~6 vs. ~11, test NR, *p* < 0.01;  P1 vs. D2: Figure 1 in Liu 2021; HAMD score = ~6 vs. ~7, test NR, *p* NR  [overall: significant time effect on HAMD score: *F*(df) = 40.73 (NR), *p* < 0.01 (Repeated-measures ANOVA)] | ↓;  ▽_NA_ |
| Morphine use | | | | | |
| Liu 2021 [30] | L | P1: 10/2019 - 12/2019  D1: 02/2020 - 04/2020 (outbreak)  D2: 05/2020- 06/2020 (post-pandemic) | Urine drug test board (positive rate %; lower is better) | P1 vs. D1: Figure 3 in Liu 2021; Positive rate (%) = ~19% vs. ~9%, test NR, *p* NR  P1 vs. D2: Figure 3 in Liu 2021; Positive rate (%) = ~19% vs. ~29%, test NR, *p* NR  [overall: significant time effect on morphine use: χ^2^ = 2.37, *p* = 0.01 (χ^2^ test)] | △_NA_;  ▽_NA_ |
| Stress | | | | | |
| Liu 2021 [30] | L | P1: 10/2019 - 12/2019  D1: 02/2020 - 04/2020 (outbreak)  D2: 05/2020- 06/2020 (post-pandemic) | PSS (lower is better; range NR) | P1 vs. D1: Figure 1 in Liu 2021; PSS score = ~14 vs. ~21, test NR, *p* < 0.01;  P1 vs. D2: Figure 1 in Liu 2021; PSS score = ~14 vs. ~18, test NR, *p* NR  [overall: significant time effect on PSS score: *F*(df) = 96.26 (NR), *p* < 0.01 (Repeated-measures ANOVA)] | ↓;  ▽_NA_ |
| Tobacco use | | | | | |
| Liu 2021 [30] | L | P1: 10/2019 - 12/2019  D1: 02/2020 - 04/2020 (outbreak)  D2: 05/2020- 06/2020 (post-pandemic) | Self-developed questionnaire (lower is better; range NR) | P1 vs. D1: Figure 3 in Liu 2021; tobacco consumption = ~24 vs. ~28, *p* = 0.03;  P1 vs. D2: Figure 3 in Liu 2021; tobacco consumption = ~24 vs. ~29, *p* < 0.01;  [overall: significant time effect on tobacco consumption: *F*(df) = 36.67 (NR), *p* < 0.01 (Repeated-measures ANOVA)] | ↓;  ↓ |
| **Peri-pandemic changes** | | | | | |
| Alcohol use | | | | | |
| Gaume 2021 [31]; *impact of pandemic on use of alcohol* | L | D1: 17-24/04/2020  D2: 04-08/05/2020 | Self-developed single item (impact of pandemic on use; 1 *[decreased]*, 3 *[usual]*, 5 *[increased]*) | D1 vs. D2: *Mdn* (*IQR*) = 3 (2-3) vs. 3 (2-3), Wilcoxon *Z* = NR, *p* NR; narrative description that no impact (i.e., medians at 3 and usual consumption)  (Wilcoxon rank-sum test) | ∎_NA_ |
| Hochstatter 2021 [32] | R-CS | D1: 31/01/2020 - 12/03/2020  D2: 24/03/2020 - 04/05/2020 | Self-developed single items (past 7 days; prevalence; lower is better) | D1 (*n* = 194) vs. D2 (*n* = 148): Prevalence = 79 (41%) vs. 60 (41%), *OR* = 0.974, *p* = 0.480 (mixed effects logistic regression model)^1^ | ↗︎ |
| Liu 2021 [30] | L | P1: 10/2019 - 12/2019  D1: 02/2020 - 04/2020 (outbreak)  D2: 05/2020- 06/2020 (post-pandemic) | Self-developed questionnaire (in ml; lower is better) | D1 vs. D2: Figure 3 in Liu 2021; alcohol use in ml = ~210 ml vs. ~140 ml, test NR, *p* < 0.01  [overall: significant time effect on alcohol use: *F*(df) = 54.45 (df), *p* < 0.01 (Repeated-measures ANOVA)] | ↑ |
| Amphetamine use | | | | | |
| Liu 2021 [30] | L | P1: 10/2019 - 12/2019  D1: 02/2020 - 04/2020 (outbreak)  D2: 05/2020- 06/2020 (post-pandemic) | Urine drug test board (positive rate %; lower is better) | D1 vs. D2: Figure 3 in Liu 2021; Positive rate (%) = ~4% vs. ~22.5%, test NR, *p* < 0.01  [overall: significant time effect on amphetamine use: χ^2^ = 3.60, *p* = 0.03 (χ^2^ test)] | ↓ |
| Anxiety symptoms | | | | | |
| Liu 2021 [30] | L | P1: 10/2019 - 12/2019  D1: 02/2020 - 04/2020 (outbreak)  D2: 05/2020- 06/2020 (post-pandemic) | HAMA (lower is better; range NR) | D1 vs. D2: Figure 1 in Liu 2021; HAMA score = ~8 vs. ~4, test NR, *p* < 0.01  [overall: significant time effect on HAMA score: *F*(df) = 53.43 (NR), *p* < 0.01 (Repeated-measures ANOVA)] | ↑ |
| Cannabis use | | | | | |
| Gaume 2021 [31]; *cannabis use* | L | D1: 17-24/04/2020  D2: 04-08/05/2020 | Self-developed single item (substance use over last week; prevalence; lower is better) | D1 (*n* = 49) vs. D2 (*n* = 51): Prevalence = 25 (51.0%) vs. 18 (35.3%), χ^2^(df) = 2.52 (1), *p* = 0.11 (χ^2^ test) | ↗︎ |
| Hochstatter 2021 [32]; *marijuana use* | R-CS | D1: 31/01/2020 - 12/03/2020  D2: 24/03/2020 - 04/05/2020 | Self-developed single items (past 7 days; prevalence; lower is better) | D1 (*n* = 194) vs. D2 (*n* = 148): Prevalence = 62 (32%) vs. 47 (32%), *OR* = 0.474, *p* = 0.291 (mixed effects logistic regression model)^1^ | ↗︎ |
| Cocaine use | | | | | |
| Gaume 2021 [31]; *cocaine use* | L | D1: 17-24/04/2020  D2: 04-08/05/2020 | Self-developed single item (substance use over last week; prevalence; lower is better) | D1 (*n* = 49) vs. D2 (*n* = 51): Prevalence = 25 (51.0%) vs. 25 (49.0%), χ^2^ NR, *p* NR (χ^2^ test); narrative result that around half of participants used cocaine across both waves | △_NA_ |
| Depressive symptoms | | | | | |
| Liu 2021 [30] | L | P1: 10/2019 - 12/2019  D1: 02/2020 - 04/2020 (outbreak)  D2: 05/2020- 06/2020 (post-pandemic) | HAMD (lower is better; range NR) | D1 vs. D2: Figure 1 in Liu 2021; HAMD score = ~11 vs. ~7, test NR, *p* < 0.01 [overall: significant time effect on HAMD score: *F*(df) = 40.73 (NR), *p* < 0.01 (Repeated-measures ANOVA)] | ↑ |
| Heroin use | | | | | |
| Gaume 2021 [31];  *heroin use* | L | D1: 17-24/04/2020  D2: 04-08/05/2020 | Self-developed single item (substance use over last week; prevalence; lower is better) | D1 (*n* = 49) vs. D2 (*n* = 51): Prevalence = 21 (42.9%) vs. 29 (56.9%), χ^2^(df) = 1.96 (1), *p* = 0.16 (χ^2^ test) | ↘︎ |
| Morphine use | | | | | |
| Liu 2021 [30] | L | P1: 10/2019 - 12/2019  D1: 02/2020 - 04/2020 (outbreak)  D2: 05/2020- 06/2020 (post-pandemic) | Urine drug test board (positive rate %; lower is better) | D1 vs. D2: Figure 3 in Liu 2021; Positive rate (%) = ~9% vs. ~29%, test NR, *p* = 0.01;  [overall: significant time effect on morphine use: χ^2^ = 2.37, *p* = 0.01 (χ^2^ test)] | ↓ |
| Psychological distress | | | | | |
| Gaume 2021 [31]; *impact of pandemic on mental health in general* | L | D1: 17-24/04/2020  D2: 04-08/05/2020 | Self-developed single items (lower is better; 1 *[no impact]* to 5 *[big impact]*) | D1 vs. D2: *Mdn* (*IQR*) = 2 (1–3) vs. 2 (1–3), Wilcoxon *Z* = NR, *p* NR, narrative results: “questions targeting the impact of the pandemic on social situation and health showed little overall impact” (Wilcoxon rank-sum test) | ∎_NA_ |
| Stress | | | | | |
| Liu 2021 [30] | L | P1: 10/2019 - 12/2019  D1: 02/2020 - 04/2020 (outbreak)  D2: 05/2020- 06/2020 (post-pandemic) | PSS (lower is better; range NR) | D1 vs. D2: Figure 1 in Liu 2021; PSS score = ~21 vs. ~16, test NR, *p* = 0.02  [overall: significant time effect on PSS score: *F*(df) = 96.26 (NR), *p* < 0.01 (Repeated-measures ANOVA)] | ↑ |
| Tobacco use | | | | | |
| Liu 2021 [30] | L | P1: 10/2019 - 12/2019  D1: 02/2020 - 04/2020 (outbreak)  D2: 05/2020- 06/2020 (post-pandemic) | Self-developed questionnaire (lower is better; range NR) | D1 vs. D2: Figure 3 in Liu 2021; tobacco consumption = ~28 vs. ~29, test NR, *p* NR  [overall: significant time effect on tobacco consumption: *F*(df) = 36.67 (NR), *p* < 0.01 (Repeated-measures ANOVA)] | ▽_NA_ |
| Use of other drugs | | | | | |
| Gaume 2021 [31]; *impact of pandemic on use of other illegal drugs (any other drugs than heroin, cocaine, cannabis, alcohol, prescription drugs; none specified)* | L | D1: 17-24/04/2020  D2: 04-08/05/2020 | Self-developed single item (impact of pandemic on use; 1 *[decreased]*, 3 *[usual]*, 5 *[increased]*) | D1 vs. D2: *Mdn* (*IQR*) = 3 (2.5-3) vs. 1 (1-3), Wilcoxon *Z* = 1.93, *p* = 0.05 (Wilcoxon rank-sum test) | ↑ |
| Hochstatter 2021 [32]; *use of other illicit drugs (heroin, prescription opioids, cocaine, methamphetamine, sedatives)* | R-CS | D1: 31/01/2020 - 12/03/2020  D2: 24/03/2020 - 04/05/2020 | Self-developed single items (past 7 days; prevalence; lower is better) | D1 (*n* = 194) vs. D2 (*n* = 148): Prevalence = 20 (10%) vs. 28 (18%), *OR* = 2.09; *p* = 0.181 (mixed effects logistic regression model)^1^ | ↘︎ |
| Use of prescription drugs | | | | | |
| Gaume 2021 [31]; *impact of pandemic on use of prescription drugs* | L | D1: 17-24/04/2020  D2: 04-08/05/2020 | Self-developed single item (impact of pandemic on use; 1 *[decreased]*, 3 *[usual]*, 5 *[increased]*) | D1 vs. D2: *Mdn* (*IQR*) = 3 (2.5-3) vs. 3 (2-3), Wilcoxon *Z* = NR, *p* NR; narrative description that no impact (i.e., medians at 3 and usual consumption) (Wilcoxon rank-sum test) | ∎_NA_ |

*Note.* Symbols in column ‘Effect direction’ represent the effect direction: ↑(direction 1): clear effect favoring pandemic (vs. pre-pandemic situation) or later peri-pandemic assessment (vs. earlier peri-pandemic assessment); ↗︎ (direction 2): unclear effect potentially favoring pandemic (vs. pre-pandemic situation) or later peri-pandemic assessment (vs. earlier peri-pandemic assessment); ∎ (direction 3): null effect; studies only narratively reporting that ‘no significant difference was observed’, studies reporting an actual null effect based on an effect estimate (e.g., Cohen’s *d* = 0); ↘︎ (direction 4): unclear effect potentially favoring pre-pandemic situation (vs. pandemic) or earlier peri-pandemic assessment (vs. later peri-pandemic assessment); ↓ (direction 5): clear effect favoring pre-pandemic situation (vs. pandemic) or earlier peri-pandemic assessment (vs. later peri-pandemic assessment); △_NA_: based on reported statistical values, probably clear or unclear effect (potentially) favoring the pandemic situation/later peri-pandemic assessment. However, vote counting used in this review was not possible since test and/or p value were not reported to assign to direction 1 or 2; ▽_NA_: based on reported statistical values, probably clear or unclear effect (potentially) favoring the pre-pandemic situation/earlier peri-pandemic assessment. However, vote counting used in this review was not possible since test and/or *p* value were not reported to assign to direction 4 or 5.

^1^ Adjusted for gender, race, age, baseline employment status, whether they have ever been incarcerated, and whether they have been diagnosed with a mental health disorder other than SUD.

*Abbreviations:* ~: approximately; D: during COVID-19 assessment (e.g., D1: first during-COVID-19 assessment); df: degrees of freedom; *F*: *F* value (*F*-test); HAMA: Hamilton Anxiety Scale; HAMD: Hamilton Depression Scale; ID: identification; *IQR*: interquartile range; L: longitudinal; *M*: mean; *Mdn*: Median; *n*: sample size; NR: not reported; *OR*: Odds Ratio; P: pre-COVID-19 assessment (e.g., P1: first pre-COVID-19 assessment); *p*: *p* value; PSS: Perceived Stress Scale; R-CS: repeated cross-sectional; χ^2^ : χ^2^ value (χ^2^test); *Z*: *Z* value (Wilcoxon test).

**Table S10.10. Addictive disorders (gambling disorders)**

| **Study ID** | **Study design** | **Assessments** | **Outcome measure (direction; range)** | **Effect summary** | **Effect direction** |
| --- | --- | --- | --- | --- | --- |
| **Peri-pandemic changes** | | | | | |
| Gambling problem symptoms | | | | | |
| Donati 2021 [33] | L | D1: NR (before lockdown)  D2: 07/04/2020 - 28/05/2020 | SOGS (total score; lower is better; range NR; cut-off for pathologic gambling: 5) | D1 vs. D2: *M*±*SD* = 10.51 ± 3.13 vs. 1.23 ± 1.32; *t*(df) = 16.42 (42), *p* < 0.001, Cohen’s *d* = 2.51 (*t*-test) | ↑ |

*Note.* Symbols in column ‘Effect direction’ represent the effect direction: ↑(direction 1): clear effect favoring pandemic (vs. pre-pandemic situation) or later peri-pandemic assessment (vs. earlier peri-pandemic assessment); ↗︎ (direction 2): unclear effect potentially favoring pandemic (vs. pre-pandemic situation) or later peri-pandemic assessment (vs. earlier peri-pandemic assessment); ∎ (direction 3): null effect; studies only narratively reporting that ‘no significant difference was observed’, studies reporting an actual null effect based on an effect estimate (e.g., Cohen’s *d* = 0); ↘︎ (direction 4): unclear effect potentially favoring pre-pandemic situation (vs. pandemic) or earlier peri-pandemic assessment (vs. later peri-pandemic assessment); ↓ (direction 5): clear effect favoring pre-pandemic situation (vs. pandemic) or earlier peri-pandemic assessment (vs. later peri-pandemic assessment); △_NA_: based on reported statistical values, probably clear or unclear effect (potentially) favoring the pandemic situation/later peri-pandemic assessment. However, vote counting used in this review was not possible since test and/or p value were not reported to assign to direction 1 or 2; ▽_NA_: based on reported statistical values, probably clear or unclear effect (potentially) favoring the pre-pandemic situation/earlier peri-pandemic assessment. However, vote counting used in this review was not possible since test and/or *p* value were not reported to assign to direction 4 or 5.

*Abbreviations:* Cohen’s *d*: effect size; D: during COVID-19 assessment (e.g., D1: first during-COVID-19 assessment); *df*: degrees of freedom; ID: identification; L: longitudinal; *M*: mean; NR: not reported; *p*: *p* value; *SD*: standard deviation; SOGS: South Oaks Gambling Screen; *t*: *t* value (*t*-test).

**Table S10.11. Mixed group (individuals with various diagnoses of mental illness)**

| **Study ID** | **Study design** | **Assessments** | **Outcome measure (direction; range)** | **Effect summary** | **Effect direction** |
| --- | --- | --- | --- | --- | --- |
| **Prior to versus during pandemic** | | | | | |
| Anxiety symptoms | | | | | |
| Johnco 2021 [34] | L | P1: ~2009 - 2019 (1 to 129 [M = 68, SD = 43] months prior to COVID-19 lockdown)  D1: 04/2020 - 05/2020 | GAI (lower is better; range NR; cut-off for anxiety disorder: 9) | P1 vs. D1: *EMM* (*SE*) = 6.03 (0.94) vs. 5.81 (0.89), *F*(df) = 1.69 (NR), *p* = 0.2, Cohen's *d* = 0.01 (analysis NR) | ↗︎ |
| Mergel 2021 [35] | L | P1: 08/2019 - 03/2020  D1: 23/03/2020 – 20/04/2020  D2: 22/06/2020 - 19/07/2020;  first two assessments reported in Schützwohl 2020 | BSI-18 (subscale anxiety; lower is better; range NR) | Group 1 (chronic disorder):  P1 vs. D1: *M*±*SD* = 0.90 ± 0.8 vs. 0.91 ± 0.8, overall test *F*(df) = 0.01 (2), *p* = 0.994, adjusted *p* = 1.00, Cohen’s *f* = 0.02 (ANOVA)^2^; post-hoc test: *p* > 0.05 (Table 4 in Mergel 2021)  P1 vs. D2: *M*±*SD* = 0.90 ± 0.8 vs. 0.91 ± 0.7, overall test *F*(df) = 0.01 (2), *p* = 0.994, adjusted *p* = 1.00, Cohen’s *f* = 0.02 (ANOVA)^2^; post-hoc test: *p* > 0.05 (Table 4 in Mergel 2021)  Group 2 (acute disorder)^1^:  P1 vs. D1: *M*±*SD* = 1.36 ± 0.9 vs. 1.03 ± 0.8, overall test *F*(df) = 6.15 (1.6), *p* = 0.007, adjusted *p* = 0.015, Cohen’s *f* = 0.46 (ANOVA)^2^; post-hoc test: *p* < 0.05 (Table 4 in Mergel 2021)  P1 vs. D2: *M*±*SD* = 1.36 ± 0.9 vs. 0.91 ± 0.8, overall test *F*(df) = 6.15 (1.6), *p* = 0.007, adjusted *p* = 0.015, Cohen’s *f* = 0.46 (ANOVA)^2^; post-hoc test: *p* < 0.05 (Table 4 in Mergel 2021) | ↘︎;  ↘︎;  ↑;  ↑ |
| Depressive symptoms | | | | | |
| Johnco 2021 [34] | L | P1: ~2009 - 2019 (1 to 129 [M = 68, SD = 43] months prior to COVID-19 lockdown)  D1: 04/2020 - 05/2020 | GDS (lower is better; range NR; cut-off for likely depressive disorder: 10) | P1 vs. D1: *EMM* (*SE*) = 10.69 (1.20) vs. 12.78 (1.30), *F*(df) = 0.21 (NR), *p* = 0.6, Cohen's *d* = 0.26 (analysis NR) | ↘︎ |
| Mergel 2021 [35] | L | P1: 08/2019 - 03/2020  D1: 23/03/2020 – 20/04/2020  D2: 22/06/2020 - 19/07/2020;  first two assessments reported in Schützwohl 2020 | BSI-18 (subscale depression; lower is better; range NR) | Group 1 (chronic disorder):  P1 vs. D1: *M*±*SD* = 1.09 ± 1.0 vs. 1.14 ± 0.9, overall test *F*(df) = 0.31 (2), *p* = 0.738, adjusted *p* = 1.00, Cohen’s *f* = 0.11 (ANOVA)^2^; post-hoc test: *p* > 0.05 (Table 4 in Mergel 2021)  P1 vs. D2: *M*±*SD* = 1.09 ± 1.0 vs. 1.02 ± 0.9, overall test *F*(df) = 0.31 (2), *p* = 0.738, adjusted *p* = 1.00, Cohen’s *f* = 0.11 (ANOVA)^2^; post-hoc test: *p* > 0.05 (Table 4 in Mergel 2021)  Group 2 (acute disorder)^1^:  P1 vs. D1: *M*±*SD* = 1.62 ± 1.2 vs. 1.05 ± 0.9, overall test *F*(df) = 8.59 (2), *p* = 0.001, adjusted *p* = 0.002, Cohen’s *f* = 0.54 (ANOVA)^2^; post-hoc test: *p* < 0.05 (Table 4 in Mergel 2021)  P1 vs. D2: *M*±*SD* = 1.62 ± 1.2 vs. 1.01 ± 0.9, overall test F(df) = 8.59 (2), *p* = 0.001, adjusted *p* = 0.002, Cohen’s f = 0.54 (ANOVA)^2^; post-hoc test: *p* < 0.05 (Table 4 in Mergel 2021) | ↘︎;  ↗︎;  ↑;  ↑ |
| Pinkham 2020 [7];  *sad/depressed* | L | P1: 04/12/2018 - 04/01/2019 (study 1) and 11/07/2019 - 21/07/2019 (study 2); pre-pandemic symptom severity averaged across all completed surveys  D1: 03/04/2020 - 04/06/2020 | EMA (lower is better; 1-7) | P1 vs. D1: *M*±*SD* = 2.95 ± 1.33 vs. 2.95 ± 2.05; *t*(df) = -0.004 (NR), *p* > 0.05, Cohen's *d* = 0.00 (*t*-test) | ∎ |
| Riblet 2021 [36]; *hopelessness* | L | P1: 10/2019 - 12/2019  P2: 11/2019 - 01/2020  D1: 02/2020 - 03/2020  D2: 23/04/2020 - 04/05/2020 | BHS (lower is better; range NR) | P1 vs. D1: *M*±*SD* = 16.5 ± 3.0 vs. 9.4 ± 5.9  P1 vs. D2: *M*±*SD* = 16.5 ± 3.0 vs. 11.9 ± 5.9  P2 vs. D1: *M*±*SD* = 10.8 ± 5.2 vs. 9.4 ± 5.9  P2 vs. D2: *M*±*SD* = 10.8 ± 5.2 vs. 11.9 ± 5.9  [Overall test: *F* NR, *p* NR (Friedman test); narrative result: not significant (“only the decreases in perceived burdensomeness […] and suicidal ideation […] were statistically significant”)] | △_NA_;  △_NA_;  △_NA_;  ▽_NA_ |
| Loneliness | | | | | |
| Riblet 2021 [36];  *thwarted belongingness* | L | P1: 10/2019 - 12/2019  P2: 11/2019 - 01/2020  D1: 02/2020 - 03/2020  D2: 23/04/2020 - 04/05/2020 | INQ-15 (subscale thwarted belongingness [TB]; lower is better; range NR) | P1 vs. D1: *M*±*SD* = 44.5 ± 10.0 vs. 36.5 ± 12.7  P1 vs. D2: *M*±*SD* = 44.5 ± 10.0 vs. 35.6 ± 10.0  P2 vs. D1: *M*±*SD* = 35.4 ± 12.3 vs. 36.5 ± 12.7  P2 vs. D2: *M*±*SD* = 35.4 ± 12.3 vs. 35.6 ± 10.0  [Overall test: overall test: *F* NR, *p* NR (ANOVA); narrative result: not significant (“only the decreases in perceived burdensomeness […] and suicidal ideation […] were statistically significant”] | △_NA_;  △_NA_;  ▽_NA_;  ▽_NA_ |
| Psychological distress | | | | | |
| Johnco 2021 [34] | L | P1: ~2009 - 2019 (1 to 129 [M = 68, SD = 43] months prior to COVID-19 lockdown)  D1: 04/2020 - 05/2020 | K10 (lower is better; 10-50; cut-off for anxiety or depressive disorder: 20) | P1 vs. D1: *EMM* (*SE*) = 19.29 (1.35) vs. 18.93 (1.52), *F*(df) = 0.50 (NR), *p* = 0.5, Cohen's *d* = 0.20 (analysis NR) | ↗︎ |
| Mergel 2021 [35] | L | P1: 08/2019 - 03/2020  D1: 23/03/2020 – 20/04/2020  D2: 22/06/2020 - 19/07/2020;  first two assessments reported in Schützwohl 2020 | BSI (GSI; lower is better; range NR) | Group 1 (chronic disorder):  P1 vs. D1: *M*±*SD* = 0.88 ± 0.7 vs. 0.90 ± 0.6, overall test *F*(df) = 0.06 (2), *p* = 0.938, adjusted *p* = 1.00, Cohen’s *f* = 0.05 (ANOVA)^2^; post-hoc test: *p* > 0.05 (Table 4 in Mergel 2021)  P1 vs. D2: *M*±*SD* = 0.88 ± 0.7 vs. 0.87 ± 0.6, overall test *F*(df) = 0.06 (2), *p* = 0.938, adjusted *p* = 1.00, Cohen’s *f* = 0.05 (ANOVA)^2^; post-hoc test: *p* > 0.05 (Table 4 in Mergel 2021)  Group 2 (acute disorder)^1^:  P1 vs. D1: *M*±*SD* = 1.34 ± 0.8 vs. 0.99 ± 0.8, overall test *F*(df) = 6.53 (1.7), *p* = 0.005, adjusted *p* = 0.014, Cohen’s *f* = 0.48 (ANOVA)^2^; post-hoc test: *p* < 0.05 (Table 4 in Mergel 2021)  P1 vs. D2: *M*±*SD* = 1.34 ± 0.8 vs. 0.89 ± 0.7, overall test *F*(df) = 6.53 (1.7), *p* = 0.005, adjusted *p* = 0.014, Cohen’s *f* = 0.48 (ANOVA)^2^; post-hoc test: *p* < 0.05 (Table 4 in Mergel 2021) | ↘︎;  ↗︎;  ↑;  ↑ |
| Seitz 2021 [37]*; general psychopathology* | L | P1: 09/2018 - 11/2019  D1: 16/04/2020 - 18/05/2020 | BSI (GSI; lower is better; 0-4; cut-off for significant psychological distress: 0.62; cut-off to differentiate between outpatients with common psychiatric disorders and healthy individuals: 0.48) | P1 vs. D1: *M*±*SD* = 1.23 ± 0.61 vs. 1.35 ± 0.72, *MD* increase baseline-pandemic (SD) = 0.12 (0.59), *t*-test NR, *p* NR; narrative result: “participants both with and without psychiatric disorders experienced increases in general psychopathology” | ▽_NA_ |
| Quality of life/well-being | | | | | |
| Johnco 2021 [34]*; quality of life (psychological health)* | L | P1: ~2009 - 2019 (1 to 129 [M = 68, SD = 43] months prior to COVID-19 lockdown)  D1: 04/2020 - 05/2020 | WHOQoL-BREF (higher is better; range NR) | P1 vs. D1: *EMM* (*SE*) = 12.56 (0.73) vs. 11.89 (0.65), *F*(df) = 0.15 (NR), *p* = 0.7, Cohen's *d* = 0.04 (analysis NR) | ↘︎ |
| Pinkham 2020 [7]); *well-being* | L | P1: 04/12/2018 - 04/01/2019 (study 1) and 11/07/2019 - 21/07/2019 (study 2); pre-pandemic symptom severity averaged across all completed surveys  D1: 03/04/2020 - 04/06/2020 | EMA (higher is better; 1-7) | P1 vs. D1: *M*±*SD* = 4.42 ± 1.10 vs. 5.05 ± 1.30; *t*(df) = 3.23 (NR), *p* < 0.05, Cohen’s *d* = 0.43 (*t*-test) | ↑ |
| Sleep quantity | | | | | |
| Pinkham 2020 [7] | L | P1: 04/12/2018 - 04/01/2019 (study 1) and 11/07/2019 - 21/07/2019 (study 2); pre-pandemic symptom severity averaged across all completed surveys  D1: 03/04/2020 - 04/06/2020 | EMA (higher is better; in hours/minutes) | P1 vs. D1: *M*±*SD* = 6.85 ± 1.61 vs. 7.06 ± 2.64; *t*(df) = 0.61 (NR), *p* > 0.05, Cohen’s *d* = 0.08 (*t*-test) | ↗︎ |
| Social functioning/relationships | | | | | |
| Johnco 2021 [34]; *quality of life (social relationships)* | L | P1: ~2009 - 2019 (1 to 129 [M = 68, SD = 43] months prior to COVID-19 lockdown)  D1: 04/2020 - 05/2020 | WHOQoL-BREF (higher is better; range NR) | P1 vs. D1: *EMM* (*SE*) = 12.22 (1.09) vs. 12.22 (0.94), *F*(df) = 2.26 (NR), *p* = 0.2, Cohen's *d* = 0.13 (analysis NR) | ∎ |
| Mergel 2021 [35]; *perceived impairment in close social relationships* | L | P1: 08/2019 - 03/2020  D1: 23/03/2020 – 20/04/2020  D2: 22/06/2020 - 19/07/2020;  first two assessments reported in Schützwohl 2020 | IMET (lower is better; 0 *[no impairment]* - 10 *[no activity possible anymore]*) | **Group 1 (chronic disorder):**  P1 vs. D1: *M*±*SD* = 4.48 ± 3.6 vs. 5.09 ± 4.0, overall test *F*(df) = 0.45 (2), *p* = 0.642, adjusted *p* = 1.00, Cohen’s *f* = 0.14 (ANOVA)^2^; post-hoc test: *p* > 0.05 (Table 3 in Mergel 2021)  P1 vs. D2: *M*±*SD* = 4.48 ± 3.6 vs. 4.35 ± 3.4, overall test *F*(df) = 0.45 (2), *p* = 0.642, adjusted *p* = 1.00, Cohen’s *f* = 0.14 (ANOVA)^2^; post-hoc test: *p* > 0.05 (Table 3 in Mergel 2021)  **Group 2 (acute disorder)^1^:**  P1 vs. D1: *M*±*SD* = 4.90 ± 3.3 vs. 5.07 ± 3.4, overall test *F*(df) = 8.44 (2), *p* = 0.001, adjusted *p* = 0.004, Cohen’s *f* = 0.54 (ANOVA)^2^; post-hoc test: *p* > 0.05 (Table 3 in Mergel 2021)  P1 vs. D2: *M*±*SD* = 4.90 ± 3.3 vs. 2.47 ± 2.8, overall test *F*(df) = 8.44 (2), *p* = 0.001, adjusted *p* = 0.004, Cohen’s *f* = 0.54 (ANOVA)^2^; **post-hoc test: *p* < 0.05** (Table 3 in Mergel 2021) | ↘︎;  ↗︎;  ↘︎;  ↑ |
| Stress | | | | | |
| Mergel 2021 [35];  *stress and extraordinary strain* | L | P1: 08/2019 - 03/2020  D1: 23/03/2020 – 20/04/2020  D2: 22/06/2020 - 19/07/2020;  first two assessments reported in Schützwohl 2020 | IMET (lower is better; 0 *[can bear the strain]* - 10 *[can no longer bear the strain]*) | **Group 1 (chronic disorder):**  P1 vs. D1: *M*±*SD* = 5.95 ± 3.3 vs. 5.63 ± 3.3, overall test *F*(df) = 0.14 (2), *p* = 0.870, adjusted *p* = 1.00, Cohen’s *f* = 0.09 (ANOVA)^2^; post-hoc test: *p* > 0.05 (Table 3 in Mergel 2021)  P1 vs. D2: *M*±*SD* = 5.95 ± 3.3 vs. 6.00 ± 3.7, overall test *F*(df) = 0.14 (2), *p* = 0.870, adjusted *p* = 1.00, Cohen’s *f* = 0.09 (ANOVA)^2^; post-hoc test: *p* > 0.05 (Table 3 in Mergel 2021)  **Group 2 (acute disorder)^1^:**  P1 vs. D1: *M*±*SD* = 7.22 ± 2.5 vs. 5.30 ± 2.6, overall test *F*(df) = 12.51 (2), *p* < 0.001, adjusted *p* < 0.001, Cohen’s *f* = 0.69 (ANOVA)^2^; **post-hoc test: *p* < 0.05** (Table 3 in Mergel 2021)  P1 vs. D2: *M*±*SD* = 7.22 ± 2.5 vs. 4.52 ± 2.6, overall test *F*(df) = 12.51 (2), *p* < 0.001, adjusted *p* < 0.001, Cohen’s *f* = 0.69 (ANOVA)^2^; **post-hoc test: *p* < 0.05** (Table 3 in Mergel 2021) | ↗︎;  ↘︎;  ↑;  ↑ |
| Suicidality | | | | | |
| Riblet 2021 [36]; *suicidal ideation* | L | P1: 10/2019 - 12/2019  P2: 11/2019 - 01/2020  D1: 02/2020 - 03/2020  D2: 23/04/2020 - 04/05/2020 | BSS (lower is better; range NR) | P1 vs. D1: *M*±*SD* = 19.9 ± 4.6 vs. 9.5 ± 9.3  P1 vs. D2: *M*±*SD* = 19.9 ± 4.6 vs. 11.0 ± 6.4  P2 vs. D1: *M*±*SD* = 11.3 ± 6.6 vs. 9.5 ± 9.3  P2 vs. D2: *M*±*SD* = 11.3 ± 6.6 vs. 11.0 ± 6.4  [Overall test: overall test: *F* NR, *p* NR (Friedman test ); narrative result: not significant (“only the decreases in perceived burdensomeness […] and suicidal ideation […] were statistically significant”] | △_NA_;  △_NA_;  △_NA_;  △_NA_ |
| **Peri-pandemic changes** | | | | | |
| Anxiety symptoms | | | | | |
| Hennigan 2021 [38] | L | D1: ~04/2020 (appr. 6 months before assessment 1)  D2: 15/10/2020 - 29/10/2020 | BAI (lower is better; range NR) | **Total group:** D1 vs. D2: *M*±*SD* = 13.04 ± 13.12 vs. 14.58 ± 12.92, *MD* = -1.54, 95% CI -6.16 to 3.08, *t*(df) = 0.69 (NR), *p* = 0.50 (*t*-test) | ↘︎ |
| Mergel 2021 [35] | L | P1: 08/2019 - 03/2020  D1: 23/03/2020 – 20/04/2020  D2: 22/06/2020 - 19/07/2020;  first two assessments reported in Schützwohl 2020 | BSI-18 (subscale anxiety; lower is better; range NR) | **Group 1 (chronic disorder):**  D1 vs. D2: *M*±*SD* = 0.91 ± 0.8 vs. 0.91 ± 0.7, overall test *F*(df) = 0.01 (2), *p* = 0.994, adjusted *p* = 1.00, Cohen’s *f* = 0.02 (ANOVA)^2^; post-hoc test: *p* > 0.05 (Table 4 in Mergel 2021)  **Group 2 (acute disorder)^1^:**  D1 vs. D2: *M*±*SD* = 1.03 ± 0.8 vs. 0.91 ± 0.8, overall test *F*(df) = 6.15 (1.6), *p* = 0.007, adjusted *p* = 0.015, Cohen’s *f* = 0.46 (ANOVA)^2^; post-hoc test: *p* > 0.05 (Table 4 in Mergel 2021) | ∎  ↗︎ |
| Seethaler 2021 [39] | L | D1: 04/2020 - 05/2020  D2: 08/2020 | Single item (probably yes vs. no) | D1 vs. D2: yes: 19 (59.38%) vs. 14 (58.33%); McNemar test NR, *p* NR | △_NA_ |
| Depressive symptoms | | | | | |
| Hennigan 2021 [38]; *impact of pandemic on mood symptoms* | L | D1: ~04/2020 (appr. 6 months before assessment 1)  D2: 15/10/2020 - 29/10/2020 | Likert scales (lower is better; 0 *[no adverse impact]* -10 *[very severe impact]*) | **Total group:** D1 vs. D2: *M*±*SD* = 3.38 ± 3.25 vs. 2.67 ± 2.58, *MD* = 0.71, 95% CI -0.88 to 2.30, *t*(df) = 0.92 (NR), *p* = 0.37 (*t*-test) | ↗︎ |
| Mergel 2021 [35] | L | P1: 08/2019 - 03/2020  D1: 23/03/2020 – 20/04/2020  D2: 22/06/2020 - 19/07/2020;  first two assessments reported in Schützwohl 2020 | BSI-18 (subscale depression; lower is better; range NR) | **Group 1 (chronic disorder):**  D1 vs. D2: *M*±*SD* = 1.14 ± 0.9 vs. 1.02 ± 0.9, overall test *F*(df) = 0.31 (2), *p* = 0.738, adjusted *p* = 1.00, Cohen’s *f* = 0.11 (ANOVA)^2^; post-hoc test: *p* > 0.05 (Table 4 in Mergel 2021)  **Group 2 (acute disorder)^1^:**  D1 vs. D2: *M*±*SD* = 1.05 ± 0.9 vs. 1.01 ± 0.9, overall test *F*(df) = 8.59 (2), *p* = 0.001, adjusted *p* = 0.002, Cohen’s *f* = 0.54 (ANOVA)^2^; post-hoc test: *p* > 0.05 (Table 4 in Mergel 2021) | ↗︎;  ↗︎ |
| Riblet 2021 [36]; *hopelessness* | L | P1: 10/2019 - 12/2019  P2: 11/2019 - 01/2020  D1: 02/2020 - 03/2020  D2: 23/04/2020 - 04/05/2020 | BHS (lower is better; range NR) | D1 vs. D2: *M*±*SD* = 9.4 ± 5.9 vs 11.9 ± 5.9  [Overall test: *F* NR, *p* NR (Friedman test); narrative result: not significant (“only the decreases in perceived burdensomeness […] and suicidal ideation […] were statistically significant”] | ▽_NA_ |
| Seethaler 2021 [39] | L | D1: 04/2020 - 05/2020  D2: 08/2020 | GDS-15 (lower is better; 0-15; cut-off ≥ 5) | D1 vs. D2: *M*±*SD* = 5.66 ± 3.48 vs. 5.50 ± 3.62; *t*-test or Wilcoxon signed-rank test NR, *p* = 0.809 | ↗︎ |
| Loneliness (thwarted belongingness) | | | | | |
| Riblet 2021 [36] | L | P1: 10/2019 - 12/2019  P2: 11/2019 - 01/2020  D1: 02/2020 - 03/2020  D2: 23/04/2020 - 04/05/2020 | INQ-15 (subscale thwarted belongingness [TB]; lower is better; range NR) | D1 vs. D2: *M*±*SD* = 36.5 ± 12.7 vs. 35.6 ± 10.0  [Overall test: overall test: *F* NR, *p* NR (ANOVA); narrative result: not significant (“only the decreases in perceived burdensomeness […] and suicidal ideation […] were statistically significant”] | △_NA_ |
| Psychological distress | | | | | |
| Hennigan 2021^3^ [38] | L | D1: ~04/2020 (appr. 6 months before assessment 1)  D2: 15/10/2020 - 29/10/2020 | GAF (higher is better; range NR) | **Total group:** D1 vs. D2: *M*±*SD* = 62.38 ± 11.72 vs. 60.21 ± 17.13, MD = 2.17 , 95% CI -4.13 to 8.46, *t*(df) = 0.71 (NR), *p* = 0.48 (*t*-test) | ↘︎ |
| Mergel 2021 [35] | L | P1: 08/2019 - 03/2020  D1: 23/03/2020 – 20/04/2020  D2: 22/06/2020 - 19/07/2020;  first two assessments reported in Schützwohl 2020 | BSI (GSI; lower is better; range NR) | **Group 1 (chronic disorder):**  D1 vs. D2: *M*±*SD* = 0.90 ± 0.6 vs. 0.87 ± 0.6, overall test *F*(df) = 0.06 (2), *p* = 0.938, adjusted *p* = 1.00, Cohen’s *f* = 0.05 (ANOVA)^2^; post-hoc test: *p* > 0.05 (Table 4 in Mergel 2021)  **Group 2 (acute disorder)^1^:**  D1 vs. D2: *M*±*SD* = 0.99 ± 0.8 vs. 0.89 ± 0.7, overall test *F*(df) = 6.53 (1.7), *p* = 0.005, adjusted *p* = 0.014, Cohen’s *f* = 0.48 (ANOVA)^2^; post-hoc test: *p* > 0.05 (Table 4 in Mergel 2021) | ↗︎;  ↗︎ |
| Seethaler 2021 [39]; *severity of illness* | L | D1: 04/2020 - 05/2020  D2: 08/2020 | CGI-S (lower is better; 1-7) | D1 vs. D2: *M*±*SD* = 3.16 ± 1.59 vs. 3.00 ± 1.41; *t*-test or Wilcoxon signed-rank test NR, *p* = 0.868 | ↗︎ |
| Quality of life | | | | | |
| Hennigan 2021 [38]; *impact of pandemic on quality of life* | L | D1: ~04/2020 (appr. 6 months before assessment 1)  D2: 15/10/2020 - 29/10/2020 | Likert scales (lower is better; 0 *[no adverse impact]* -10 *[very severe impact]*) | **Total group:** D1 vs. D2: *M*±*SD* = 4.33 ± 2.63 vs. 3.83 ± 2.84, *MD* = 0.50, 95% CI -0.92 to 1.92, *t*(df) = 0.73 (NR), *p* = 0.47 (*t*-test) | ↗︎ |
| Social functioning/relationships | | | | | |
| Hennigan 2021 [38]; *impact of pandemic on social functioning* | L | D1: ~04/2020 (appr. 6 months before assessment 1)  D2: 15/10/2020 - 29/10/2020 | Likert scales (lower is better; 0 *[no adverse impact]* -10 *[very severe impact]*) | **Total group:** D1 vs. D2: *M*±*SD* = 4.54 ± 2.94 vs. 4.00 ± 2.93, *MD* = 0.54, 95% CI -1.07 to 2.15, *t*(df) = 0.70 (NR), *p* = 0.49 (*t*-test) | ↗︎ |
| Mergel 2021 [35]; *perceived impairment in close social relationships* | L | P1: 08/2019 - 03/2020  D1: 23/03/2020 – 20/04/2020  D2: 22/06/2020 - 19/07/2020;  first two assessments reported in Schützwohl 2020 | IMET (lower is better; 0 *[no impairment]* - 10 *[no activity possible anymore]*) | **Group 1 (chronic disorder):**  D1 vs. D2: *M*±*SD* = 5.09 ± 4.0 vs. 4.35 ± 3.4, overall test *F*(df) = 0.45 (2), *p* = 0.642, adjusted *p* = 1.00, Cohen’s *f* = 0.14 (ANOVA)^2^; post-hoc test: *p* > 0.05 (Table 3 in Mergel 2021)  **Group 2 (acute disorder)^1^:**  D1 vs. D2: *M*±*SD* = 5.07 ± 3.4 vs. 2.47 ± 2.8, overall test *F*(df) = 8.44 (2), *p* = 0.001, adjusted *p* = 0.004, Cohen’s *f* = 0.54 (ANOVA)^2^; **post-hoc test: *p* < 0.05** (Table 3 in Mergel 2021) | ↗︎;  ↑ |
| Stress | | | | | |
| Mergel 2021 [35];  *stress and extraordinary strain* | L | P1: 08/2019 - 03/2020  D1: 23/03/2020 – 20/04/2020  D2: 22/06/2020 - 19/07/2020;  first two assessments reported in Schützwohl 2020 | IMET (lower is better; 0 *[can bear the strain]* – 10 *[can no longer bear the strain]*) | **Group 1 (chronic disorder):**  D1 vs. D2: *M*±*SD* = 5.63 ± 3.3 vs. 6.00 ± 3.7, overall test *F*(df) = 0.14 (2), *p* = 0.870, adjusted *p* = 1.00, Cohen’s *f* = 0.09 (ANOVA)^2^; post-hoc test: *p* > 0.05 (Table 3 in Mergel 2021)  **Group 2 (acute disorder)^1^:**  D1 vs. D2: *M*±*SD* = 5.30 ± 2.6 vs. 4.52 ± 2.6, overall test *F*(df) = 12.51 (2), *p* < 0.001, adjusted *p* < 0.001, Cohen’s *f* = 0.69 (ANOVA)^2^; post-hoc test: *p* > 0.05 (Table 3 in Mergel 2021) | ↘︎;  ↗︎ |
| Suicidality | | | | | |
| Riblet 2021 [36]; *suicidal ideation* | L | P1: 10/2019 - 12/2019  P2: 11/2019 - 01/2020  D1: 02/2020 - 03/2020  D2: 23/04/2020 - 04/05/2020 | BSS (lower is better; range NR) | D1 vs. D2: *M*±*SD* = 9.5 ± 9.3 vs. 11.0 ± 6.4  [Overall test: overall test: *F* NR, *p* NR (Friedman test); narrative result: not significant (“only the decreases in perceived burdensomeness […] and suicidal ideation […] were statistically significant”] | ▽_NA_ |
| Seethaler 2021 [39]; *current suicidality (suicidal thoughts)* | L | D1: 04/2020 - 05/2020  D2: 08/2020 | Single item (prevalence; lower is better) | D1 vs. D2: Prevalence = 11 (34.38%) vs. 4 (16.67%), McNemar test NR, *p* NR | △_NA_ |

*Note.* Symbols in column ‘Effect direction’ represent the effect direction: ↑(direction 1): clear effect favoring pandemic (vs. pre-pandemic situation) or later peri-pandemic assessment (vs. earlier peri-pandemic assessment); ↗︎ (direction 2): unclear effect potentially favoring pandemic (vs. pre-pandemic situation) or later peri-pandemic assessment (vs. earlier peri-pandemic assessment); ∎ (direction 3): null effect; studies only narratively reporting that ‘no significant difference was observed’, studies reporting an actual null effect based on an effect estimate (e.g., Cohen’s *d* = 0); ↘︎ (direction 4): unclear effect potentially favoring pre-pandemic situation (vs. pandemic) or earlier peri-pandemic assessment (vs. later peri-pandemic assessment); ↓ (direction 5): clear effect favoring pre-pandemic situation (vs. pandemic) or earlier peri-pandemic assessment (vs. later peri-pandemic assessment); △_NA_: based on reported statistical values, probably clear or unclear effect (potentially) favoring the pandemic situation/later peri-pandemic assessment. However, vote counting used in this review was not possible since test and/or p value were not reported to assign to direction 1 or 2; ▽_NA_: based on reported statistical values, probably clear or unclear effect (potentially) favoring the pre-pandemic situation/earlier peri-pandemic assessment. However, vote counting used in this review was not possible since test and/or *p* value were not reported to assign to direction 4 or 5.

^1^ Acute disorder (group 2): individuals who were receiving acute psychiatric or psychotherapeutic treatment **at the time of the initial survey (i.e., 08/2019 - 03/2020; within three days of admission).**

^2^ Repeated-measures ANOVAs with post-hoc pairwise comparisons (Bonferroni-Holm method to obtain adjusted *p* values).

^3^ Psychological distress in Hennigan 2021 also assessed using Clinical Global Impression-Severity [CGI-S]); however, results not reported in publication.

*Abbreviations:* ~: approximately; ANOVA: analysis of variance; BAI: Beck Anxiety Inventory; BHS: Beck Hopelessness Scale; BSI(-18): Brief Symptom Inventory(-18); BSS: Beck Scale for Suicidal Ideation; CGI-S: Clinical Global Impression – Severity; Cohen’s *d*: effect size; Cohen’s *f*: effect size; CI: confidence interval; D: during COVID-19 assessment (e.g., D1: first during-COVID-19 assessment); *df*: degrees of freedom; EMA: Ecological Momentary Assessment; *EMM*: estimated marginal means; *F*: *F* value (*F*-test); GAF: Global Assessment of Function; GAI: Geriatric Anxiety Inventory; GDS(-15): Geriatric Depression Scale(-15); GSI: Global Severity Index; ID: identification; IMET: Index for the Assessment of Health Impairments; INQ-15: Interpersonal Needs Questionnaire-15; K10: Kessler-10; L: longitudinal; *M*: mean; *MD*: mean difference; NR: not reported; P: pre-COVID.

**References**

1. Goldfarb, Y.; Gal, E.; Golan, O. I Implications of employment changes caused by COVID-19 on mental health and work-related pychological need satisfaction of Autistic employees: A mixed-methods longitudinal study. *J Autism Dev Disord* **2022**, *52*, 89-102, doi:10.1007/s10803-021-04902-3.
2. Adams, R.E.; Zheng, S.; Taylor, J.L.; Bishop, S.L. Ten weeks in: COVID-19-related distress in adults with autism spectrum disorder. *Autism* **2021**, *25*, 2140-2145, doi:10.1177/13623613211005919.
3. Lugo-Marín, J.; Gisbert-Gustemps, L.; Setien-Ramos, I.; Español-Martín, G.; Ibañez-Jimenez, P.; Forner-Puntonet, M.; Arteaga-Henríquez, G.; Soriano-Día, A.; Duque-Yemail, J.D.; Ramos-Quiroga, J.A. COVID-19 pandemic effects in people with Autism Spectrum Disorder and their caregivers: Evaluation of social distancing and lockdown impact on mental health and general status. *Res Autism Spectr Disord* **2021**, *83*, 101757, doi:10.1016/j.rasd.2021.101757.
4. Bal, V.H.; Wilkinson, E.; White, L.C.; Law, J.K.; Feliciano, P.; Chung, W.K. Early pandemic experiences of autistic adults: Predictors of psychological distress. *Autism Res* **2021**, *14*, 1209-1219, doi:10.1002/aur.2480.
5. Brondino, N.; Damiani, S.; Politi, P. Effective strategies for managing COVID-19 emergency restrictions for adults with severe ASD in a daycare center in Italy. *Brain Sci* **2020**, *10*, doi:10.3390/brainsci10070436.
6. Kott, A.; Daniel, D.G. P.508 COVID-19 impact on entry symptom severity in schizophrenia clinical trials – preliminary data. *European Neuropsychopharmacology* **2020**, *40*, S286-S287, doi:10.1016/j.euroneuro.2020.09.372.
7. Pinkham, A.E.; Ackerman, R.A.; Depp, C.A.; Harvey, P.D.; Moore, R.C. A longitudinal investigation of the effects of the COVID-19 pandemic on the mental health of individuals with pre-existing severe mental illnesses. *Psychiatry Res* **2020**, *294*, 113493, doi:10.1016/j.psychres.2020.113493.
8. Strauss, G.P.; Macdonald, K.I.; Ruiz, I.; Raugh, I.M.; Bartolomeo, L.A.; James, S.H. The impact of the COVID-19 pandemic on negative symptoms in individuals at clinical high-risk for psychosis and outpatients with chronic schizophrenia. *Eur Arch Psychiatry Clin Neurosci* **2022**, *272*, 17-27, doi:10.1007/s00406-021-01260-0.
9. Cordellieri, P.; Barchielli, B.; Masci, V.; Viani, F.; de Pinto, I.; Priori, A.; Torriccelli, F.D.; Cosmo, C.; Ferracuti, S.; Giannini, A.M.; et al. Psychological health status of psychiatric patients living in treatment communities before and during the COVID-19 lockdown: A brief report. *Int J Environ Res Public Health* **2021**, *18*, doi:10.3390/ijerph18073567.
10. Ma, J.; Hua, T.; Zeng, K.; Zhong, B.; Wang, G.; Liu, X. Influence of social isolation caused by coronavirus disease 2019 (COVID-19) on the psychological characteristics of hospitalized schizophrenia patients: a case-control study. *Translational Psychiatry* **2020**, *10*, 411, doi:10.1038/s41398-020-01098-5.
11. Wynn, J.K.; McCleery, A.; Novacek, D.; Reavis, E.A.; Tsai, J.; Green, M.F. Clinical and functional effects of the COVID-19 pandemic and social distancing on vulnerable veterans with psychosis or recent homelessness. *J Psychiatr Res* **2021**, *138*, 42-49, doi:10.1016/j.jpsychires.2021.03.051.
12. Daly, M.; Robinson, E. Psychological distress and adaptation to the COVID-19 crisis in the United States. *J Psychiatr Res* **2021**, *136*, 603-609, doi:10.1016/j.jpsychires.2020.10.035.
13. Ma, J.; Jiang, T.; Huang, H.; Li, R.; Zhang, L.; Liu, L.; Liu, X. Mental symptoms and stress of hospitalized schizophrenia patients with 2019 novel coronavirus disease: An observation study. *Front Psychiatry* **2021**, *12*, 557611, doi:10.3389/fpsyt.2021.557611.
14. Orhan, M.; Korten, N.; Paans, N.; de Walle, B.; Kupka, R.; van Oppen, P.; Kok, A.; Sonnenberg, C.; Schouws, S.; Dols, A. Psychiatric symptoms during the COVID-19 outbreak in older adults with bipolar disorder. *Int J Geriatr Psychiatry* **2021**, *36*, 892-900, doi:10.1002/gps.5489.
15. Yocum, A.K.; Zhai, Y.; McInnis, M.G.; Han, P. Covid-19 pandemic and lockdown impacts: A description in a longitudinal study of bipolar disorder. *J Affect Disord* **2021**, *282*, 1226-1233, doi:10.1016/j.jad.2021.01.028.
16. Carta, M.G.; Ouali, U.; Perra, A.; Ben Cheikh Ahmed, A.; Boe, L.; Aissa, A.; Lorrai, S.; Cossu, G.; Aresti, A.; Preti, A.; et al. Living with bipolar disorder in the time of Covid-19: Biorhythms during the severe lockdown in Cagliari, Italy, and the moderate lockdown in Tunis, Tunisia. *Front Psychiatry* **2021**, *12*, 634765, doi:10.3389/fpsyt.2021.634765.
17. Hamm, M.E.; Brown, P.J.; Karp, J.F.; Lenard, E.; Cameron, F.; Dawdani, A.; Lavretsky, H.; Miller, J.P.; Mulsant, B.H.; Pham, V.T.; et al. xperiences of American older adults with pre-existing depression during the beginnings of the COVID-19 pandemic: A multicity, mixed-methods study. *Am J Geriatr Psychiatry* **2020**, *28*, 924-932, doi:10.1016/j.jagp.2020.06.013.
18. Pan, K.Y.; Kok, A.A.L.; Eikelenboom, M.; Horsfall, M.; Jörg, F.; Luteijn, R.A.; Rhebergen, D.; Oppen, P.V.; Giltay, E.J.; Penninx, B. The mental health impact of the COVID-19 pandemic on people with and without depressive, anxiety, or obsessive-compulsive disorders: a longitudinal study of three Dutch case-control cohorts. *Lancet Psychiatry* **2021**, *8*, 121-129, doi:10.1016/s2215-0366(20)30491-0.
19. Chakraborty, A.; Karmakar, S. Impact of COVID-19 on Obsessive Compulsive Disorder (OCD). *Iranian Journal of Psychiatry* **2020**, *15*, 256-259, doi:10.18502/ijps.v15i3.3820.
20. Khosravani, V.; Aardema, F.; Samimi Ardestani, S.M.; Sharifi Bastan, F. The impact of the coronavirus pandemic on specific symptom dimensions and severity in OCD: A comparison before and during COVID-19 in the context of stress responses. *J Obsessive Compuls Relat Disord* **2021**, *29*, 100626, doi:10.1016/j.jocrd.2021.100626.
21. Matsunaga, H.; Mukai, K.; Yamanishi, K. Acute impact of COVID-19 pandemic on phenomenological features in fully or partially remitted patients with obsessive-compulsive disorder. *Psychiatry Clin Neurosci* **2020**, *74*, 565-566, doi:10.1111/pcn.13119.
22. Sharma, L.P.; Balachander, S.; Thamby, A.; Bhattacharya, M.; Kishore, C.; Shanbhag, V.; Sekharan, J.T.; Narayanaswamy, J.C.; Arumugham, S.S.; Reddy, J.Y.C. Impact of the COVID-19 pandemic on the short-term course of obsessive-compulsive disorder. *J Nerv Ment Dis* **2021**, *209*, 256-264, doi:10.1097/nmd.0000000000001318.
23. Davide, P.; Andrea, P.; Martina, O.; Andrea, E.; Davide, D.; Mario, A. The impact of the COVID-19 pandemic on patients with OCD: Effects of contamination symptoms and remission state before the quarantine in a preliminary naturalistic study. *Psychiatry Res* **2020**, *291*, 113213, doi:10.1016/j.psychres.2020.113213.
24. Rutherford, B.R.; Choi, C.J.; Chrisanthopolous, M.; Salzman, C.; Zhu, C.; Montes-Garcia, C.; Liu, Y.; Brown, P.J.; Yehuda, R.; Flory, J.; et al. The COVID-19 pandemic as a traumatic stressor: Mental health responses of older adults with chronic PTSD. *Am J Geriatr Psychiatry* **2021**, *29*, 105-114, doi:10.1016/j.jagp.2020.10.010.
25. Giel, K.E.; Schurr, M.; Zipfel, S.; Junne, F.; Schag, K. Eating behaviour and symptom trajectories in patients with a history of binge eating disorder during COVID-19 pandemic. *Eur Eat Disord Rev* **2021**, *29*, 657-662, doi:10.1002/erv.2837.
26. Castellini, G.; Cassioli, E.; Rossi, E.; Innocenti, M.; Gironi, V.; Sanfilippo, G.; Felciai, F.; Monteleone, A.M.; Ricca, V. The impact of COVID-19 epidemic on eating disorders: A longitudinal observation of pre versus post psychopathological features in a sample of patients with eating disorders and a group of healthy controls. *Int J Eat Disord* **2020**, *53*, 1855-1862, doi:10.1002/eat.23368.
27. Machado, P.P.P.; Pinto-Bastos, A.; Ramos, R.; Rodrigues, T.F.; Louro, E.; Gonçalves, S.; Brandão, I.; Vaz, A. Impact of COVID-19 lockdown measures on a cohort of eating disorders patients. *J Eat Disord* **2020**, *8*, 57, doi:10.1186/s40337-020-00340-1.
28. Nisticò, V.; Bertelli, S.; Tedesco, R.; Anselmetti, S.; Priori, A.; Gambini, O.; Demartini, B. The psychological impact of COVID-19-related lockdown measures among a sample of Italian patients with eating disorders: a preliminary longitudinal study. *Eat Weight Disord* **2021**, *26*, 2771-2777, doi:10.1007/s40519-021-01137-0.
29. Leenaerts, N.; Vaessen, T.; Ceccarini, J.; Vrieze, E. How COVID-19 lockdown measures could impact patients with bulimia nervosa: Exploratory results from an ongoing experience sampling method study. *Eat Behav* **2021**, *41*, 101505, doi:10.1016/j.eatbeh.2021.101505.
30. Liu, X.; Jin, X.; Zhang, Y.; Zhang, L.; Li, Y.; Ma, J. Effect of coronavirus disease 2019 on the psychology and behavior of patients on methadone maintenance treatment in Wuhan, China: A clinical observational study. *Front Psychiatry* **2021**, *12*, 653662, doi:10.3389/fpsyt.2021.653662.
31. Gaume, J.; Schmutz, E.; Daeppen, J.B.; Zobel, F. Evolution of the illegal substances market and substance users' social situation and health during the COVID-19 pandemic. *Int J Environ Res Public Health* **2021**, *18*, doi:10.3390/ijerph18094960.
32. Hochstatter, K.R.; Akhtar, W.Z.; Dietz, S.; Pe-Romashko, K.; Gustafson, D.H.; Shah, D.V.; Krechel, S.; Liebert, C.; Miller, R.; El-Bassel, N.; et al. Potential influences of the COVID-19 pandemic on drug use and HIV care among people living with HIV and substance use disorders: experience from a pilot mHealth intervention. *AIDS Behav* **2021**, *25*, 354-359, doi:10.1007/s10461-020-02976-1.
33. Donati, M.A.; Cabrini, S.; Capitanucci, D.; Primi, C.; Smaniotto, R.; Avanzi, M.; Quadrelli, E.; Bielli, G.; Casini, A.; Roaro, A. Being a gambler during the COVID-19 pandemic: A study with Italian patients and the effects of reduced exposition. *Int J Environ Res Public Health* **2021**, *18*, doi:10.3390/ijerph18020424.
34. Johnco, C.J.; Chen, J.T.H.; Muir, C.; Strutt, P.; Dawes, P.; Siette, J.; Dias, C.B.; Hillebrandt, H.; Maurice, O.; Wuthrich, V.M. Long-term relapse rates after cognitive behaviour therapy for anxiety and depressive disorders among older adults: A follow-up study during COVID-19. *Australas J Ageing* **2021**, *40*, 208-212, doi:10.1111/ajag.12928.
35. Mergel, E.; Schützwohl, M. A longitudinal study on the COVID-19 pandemic and its divergent effects on social participation and mental health across different study groups with and without mental disorders. *Soc Psychiatry Psychiatr Epidemiol* **2021**, *56*, 1459-1468, doi:10.1007/s00127-021-02025-9.
36. Riblet, N.B.; Stevens, S.P.; Shiner, B.; Cornelius, S.; Forehand, J.; Scott, R.C.; Watts, B.V. Longitudinal examination of COVID-19 public health measures on mental health for rural patients with serious mental illness. *Mil Med* **2021**, *186*, e956-e961, doi:10.1093/milmed/usaa559.
37. Seitz, K.I.; Bertsch, K.; Herpertz, S.C. A prospective study of mental health during the COVID-19 pandemic in childhood trauma-exposed individuals: Social support matters. *J Trauma Stress* **2021**, *34*, 477-486, doi:10.1002/jts.22660.
38. Hennigan, K.; McGovern, M.; Plunkett, R.; Costello, S.; McDonald, C.; Hallahan, B. A longitudinal evaluation of the impact of the COVID-19 pandemic on patients with pre-existing anxiety disorders. *Ir J Psychol Med* **2021**, *38*, 258-265, doi:10.1017/ipm.2021.32.
39. Seethaler, M.; Just, S.; Stötzner, P.; Bermpohl, F.; Brandl, E.J. Psychosocial Impact of COVID-19 pandemic in elderly psychiatric patients: a longitudinal study. *Psychiatr Q* **2021**, *92*, 1439-1457, doi:10.1007/s11126-021-09917-8.
