# Supplementary material for: Mental Health Impact of Early Stages of the COVID-19 Pandemic on Individuals with Pre-Existing Mental Disorders: A Systematic Review of Longitudinal Research
Source: Int J Environ Res Public Health. 2023 Jan 4;20(2):948. doi: 10.3390/ijerph20020948 (PMC9858748; doi:10.3390/ijerph20020948)
Supplement: Supplementary file 1 [file ijerph-20-00948-s001.zip › Supplementary material/Supplementary material file 2.docx]

**Supplementary material file 2. Search strategy**

Date of last search for all databases was 31 May 2021.

**PsycINFO (Ovid)** – APA PsycInfo 1806 to May Week 4 2021 (1956 records)

1. covid.mp.
2. COVID-19.mp.
3. COVID19.mp.
4. ("SARS-CoV-2" or "SARS-CoV2" or SARSCoV2 or "SARSCoV-2" or "SARS coronavirus 2").mp.
5. (2019 nCoV or 2019nCoV or 2019-novel CoV or nCov 2019 or nCov 19).mp.
6. ("severe acute respiratory syndrome coronavirus 2" or "novel coronavirus disease" or "novel corona virus disease" or "corona virus disease 2019" or "coronavirus disease 2019" or "novel coronavirus pneumonia" or "novel corona virus pneumonia").mp.
7. (severe acute respiratory syndrome coronavirus 2).mp.
8. or/1-7
9. Anxiety Disorders/
10. exp Bipolar Disorder/
11. Chronic Mental Illness/
12. Mental Disorders/
13. Major Depression/
14. Posttraumatic Stress Disorder/
15. Psychosis/
16. Psychiatric Hospitals/
17. Schizophrenia/
18. Serious Mental Illness/
19. exp Somatoform Disorders/
20. "Substance Related and Addictive Disorders"/
21. "Substance Use Disorder"/
22. (mental health adj1 (patient* or disorder* or care or clinic* or condition* or service*)).tw.
23. (mental adj2 (disorder* or illness* or disease* or healthcare)).tw.
24. (psychiatric or psychiatry or psychological crisis or depression* or depressive or anxiety disorder* or addiction disorder* or phobic disorder* or neurotic disorder* or bipolar or panic disorder* or panic attack* or paranoi* or psychotic or psychosis or psychoses or schizo* or delusional disorder* or neurose* or neurosi* or psychoneuros* or psycho neuros* or traumatic stress disorder* or posttraumatic stress disorder* or PTSD or substance related disorder* or "substance use" or drug abuse or drug addiction or substance abuse or alcohol related disorder* or alcoholism or "alcohol use" or gambling or gaming disorder* or somatoform disorder* or social phobia* or phobic disorder* or anorexia or bulimia or eating disorder* or obsessive compulsive disorder* or autism or autistic disorder* or asperger*).tw.
25. or/9-24
26. 8 and 25

**Web of Science** – 1945-present; Emerging Citation Index and Science Citation Index (4750 records)

1. AB=((COVID OR "COVID-19" OR COVID19) OR ("SARS-CoV-2" OR "SARS-CoV2" OR SARSCoV2 OR "SARSCoV-2" OR "SARS coronavirus 2") OR ("2019 nCoV" OR "2019nCoV" OR "2019-novel CoV" OR "nCov 2019" OR "nCov 19") OR ("severe acute respiratory syndrome coronavirus 2" OR "novel coronavirus disease" OR "novel corona virus disease" OR "corona virus disease 2019" OR "coronavirus disease 2019" OR "novel coronavirus pneumonia" OR "novel corona virus pneumonia") OR ("severe acute respiratory syndrome coronavirus 2")) OR TI=((COVID OR "COVID-19" OR COVID19) OR ("SARS-CoV-2" OR "SARS-CoV2" OR SARSCoV2 OR "SARSCoV-2" OR "SARS coronavirus 2") OR ("2019 nCoV" OR 2019nCoV OR "2019-novel CoV" OR "nCov 2019" OR "nCov 19") OR ("severe acute respiratory syndrome coronavirus 2" OR "novel coronavirus disease" OR "novel corona virus disease" OR "corona virus disease 2019" OR "coronavirus disease 2019" OR "novel coronavirus pneumonia" OR "novel corona virus pneumonia") OR ("severe acute respiratory syndrome coronavirus 2"))
2. AB=(("mental health" NEAR/1 (patient* OR disorder* OR care OR clinic* OR condition* OR service*)) OR (mental NEAR/2 (disorder* OR illness* OR disease* OR healthcare)) OR (psychiatric OR psychiatry OR "psychological crisis" OR depression* OR depressive OR "anxiety disorder*" OR "addiction disorder*" OR "phobic disorder*" OR "neurotic disorder*" OR bipolar OR "panic disorder*" OR "panic attack*" OR paranoi* OR psychotic OR psychosis OR psychoses OR schizo* OR "delusional disorder*" OR neurose* OR neurosi* OR psychoneuros* OR "psycho-neuros*" OR "traumatic stress disorder*" OR "posttraumatic stress disorder*" OR PTSD OR "substance-related disorder*" OR "substance use" OR "drug abuse" OR "drug addiction" OR "substance abuse" OR "alcohol-related disorder*" OR alcoholism OR "alcohol use" OR gambling OR "gaming disorder*" OR "somatoform disorder*" OR "social phobia*" OR "phobic disorder*" OR anorexia OR bulimia OR "eating disorder*" OR "obsessive-compulsive disorder*" OR autism OR "autistic disorder*" OR asperger*)) OR TI=(("mental health" NEAR/1 (patient* OR disorder* OR care OR clinic* OR condition* OR service*)) OR (mental NEAR/2 (disorder* OR illness* OR disease* OR healthcare)) OR (psychiatric OR psychiatry OR "psychological crisis" OR depression* OR depressive OR "anxiety disorder*" OR "addiction disorder*" OR "phobic disorder*" OR "neurotic disorder*" OR bipolar OR "panic disorder*" OR "panic attack*" OR paranoi* OR psychotic OR psychosis OR psychoses OR schizo* OR "delusional disorder*" OR neurose* OR neurosi* OR psychoneuros* OR "psycho-neuros*" OR "traumatic stress disorder*" OR "posttraumatic stress disorder*" OR PTSD OR "substance-related disorder*" OR "substance use" OR "drug abuse" OR "drug addiction" OR "substance abuse" OR "alcohol-related disorder*" OR alcoholism OR "alcohol use" OR gambling OR "gaming disorder*" OR "somatoform disorder*" OR "social phobia*" OR "phobic disorder*" OR anorexia OR bulimia OR "eating disorder*" OR "obsessive-compulsive disorder*" OR autism OR "autistic disorder*" OR asperger*))
3. #1 AND #2

**WHO Global literature on COVID-19 database** (2167 records)

(exclusion of primary sources already included in the Cochrane COVID-19 Study Register or searched separately: ICTRP, Embase, Medline, Scopus, PubMed, bioRxiv, Web of Science, APA PsycINFO, PMC)

"mental health patients" or "mental health disorder" or "mental health disorders" or "mental health care" or "mental healthcare" or "mental health clinic" or "mental health clinics" or "mental health service" or "mental health services" or "mental disorder" or "mental disorders" or "mental disease" or "mental diseases" or "mental illness" or "mental illnesses" or "mental health condition" or "mental health conditions" or psychiatric or psychiatry or "psychological crisis" or depression* or depressive or "addiction disorder" or "addiction disorders" or "anxiety disorder" or "anxiety disorders" or "phobic disorder" or "phobic disorders" or "neurotic disorder" or "neurotic disorders" or bipolar or "panic disorder" or "panic disorders" or "panic attack" or "panic attacks" or paranoi* or psychotic or psychosis or psychoses or schizo* or "delusional disorder" or "delusional disorders" or neurose* or neurosi* or psychoneuros* or "traumatic stress disorder" or "posttraumatic stress disorder" or "traumatic stress disorders" or "posttraumatic stress disorders" or PTSD or "substance related disorders" or "substance related disorder" or "substance use" or "drug abuse" or "drug addiction" or "substance abuse" or "alcohol related disorder" or "alcohol related disorders" or alcoholism or "alcohol use" or gambling or "gaming disorder" or "gaming disorders" or "social phobia" or "social phobias" or "somatoform disorder" or "somatoform disorders" or "phobic disorder" or "phobic disorders" or anorexia or bulimia or "eating disorder" or "eating disorders" or "obsessive compulsive disorder" or "obsessive compulsive disorders" or autism or "autistic disorder" or "autistic disorders" or asperger*

**Cochrane COVID-19 Study Register (covid-19.cochrane.org)** (5520 records)

"mental health patients" or "mental health disorder" or "mental health disorders" or "mental health care" or "mental healthcare" or "mental health clinic" or "mental health clinics" or "mental health service" or "mental health services" or "mental disorder" or "mental disorders" or "mental disease" or "mental diseases" or "mental illness" or "mental illnesses" or "mental health condition" or "mental health conditions" or psychiatric or psychiatry or "psychological crisis" or depression* or depressive or "addiction disorder" or "addiction disorders" or "anxiety disorder" or "anxiety disorders" or "phobic disorder" or "phobic disorders" or "neurotic disorder" or "neurotic disorders" or bipolar or "panic disorder" or "panic disorders" or "panic attack" or "panic attacks" or paranoi* or psychotic or psychosis or psychoses or schizo* or "delusional disorder" or "delusional disorders" or neurose* or neurosi* or psychoneuros* or "traumatic stress disorder" or "posttraumatic stress disorder" or "traumatic stress disorders" or "posttraumatic stress disorders" or PTSD or "substance related disorders" or "substance related disorder" or "substance use" or "drug abuse" or "drug addiction" or "substance abuse" or "alcohol related disorder" or "alcohol related disorders" or alcoholism or "alcohol use" or gambling or "gaming disorder" or "gaming disorders" or "social phobia" or "social phobias" or "somatoform disorder" or "somatoform disorders" or "phobic disorder" or "phobic disorders" or anorexia or bulimia or "eating disorder" or "eating disorders" or "obsessive compulsive disorder" or "obsessive compulsive disorders" or autism or "autistic disorder" or "autistic disorders" or asperger*
