# Supplementary material for: Mental Health Impact of Early Stages of the COVID-19 Pandemic on Individuals with Pre-Existing Mental Disorders: A Systematic Review of Longitudinal Research
Source: Int J Environ Res Public Health. 2023 Jan 4;20(2):948. doi: 10.3390/ijerph20020948 (PMC9858748; doi:10.3390/ijerph20020948)
Supplement: Supplementary file 1 [file ijerph-20-00948-s001.zip › Supplementary material/Supplementary material file 3.docx]

**Supplementary material file 3. Eligibility criteria for the systematic review**

**Table S3.1. Eligibility criteria for the systematic review**

| Population | Inclusion:   - Individuals aged 18 years and older, with pre-existing mental disorders according to ICD-10 or DSM-5 classification systems:   - Autism spectrum disorders (ASD)   - Schizophrenia, schizotypal and delusional disorders / schizophrenia spectrum and other psychotic disorders (e.g., schizophrenia, psychotic disorders, schizoaffective disorders)   - Bipolar disorders   - Depressive disorders (e.g., major depressive disorder [MDD], persistent depressive disorder/dysthymic disorder)   - Anxiety disorders (e.g., generalized anxiety disorders, phobic disorders like social phobia, panic disorders)   - Obsessive-compulsive disorders (OCD)   - Post-traumatic stress disorders (PTSD)   - Somatoform / somatic symptom disorders   - Eating disorders (e.g., anorexia nervosa)   - Substance-related disorders/substance use disorder (SUD)   - Gambling / gaming disorders - Participants have been diagnosed with the respective mental illness before the COVID-19 pandemic (e.g., based on a structured clinical interview like the Mini International Neuropsychiatric Interview [M.I.N.I.] or the Structured Clinical Interview for DSM-5 [SCID-5]) OR   participants have been in psychiatric and/or psychotherapeutic treatment before the COVID-19 pandemic (and have been diagnosed at the start of this treatment, for example)   - Mixed samples: In case a study includes participants with a broad range of mental diagnoses (e.g., only description that “patients with mental disorders” or “psychiatric patients” were included), this study was considered for this review if at least 75% of the included patients could be attributed to any of the above-mentioned types/clusters of mental illness, such as depressive disorders - Irrespective of country, sex, health status (e.g., severity of mental disorder, mental disorder in an acute phase or in remission, comorbidities), and setting of study conduction (e.g., clinical setting, outpatient setting)   Exclusion:   - Samples solely comprising other population groups (e.g., general population, healthcare workers, other patient populations) |
| --- | --- |
| Exposure | Inclusion:   - Exposure to COVID-19 pandemic (i.e., survey period after the first officially registered COVID-19 case in the respective country based on national dates on daily COVID-19 cases and deaths by date reported to World Health Organization; https://covid19.who.int/info/)   Exclusion:   - Exposure to other epidemic or pandemic infectious disease outbreaks (e.g., SARS, MERS, Ebola, HIV, influenza) |
| Comparator | Inclusion:   - Pre-pandemic assessment of mental health (i.e., before first COVID-19 case in respective country) - Earlier peri-pandemic assessment of mental health   Exclusion:   - Exposure to / assessment during any other pandemics, epidemics, or other macro-stressors (e.g., natural disasters) |
| Outcomes | Inclusion:   - Primary outcomes: disorder-specific outcomes such as:   - Symptoms of autism spectrum disorder   - Symptoms of schizophrenia and psychotic disorders   - Symptoms of bipolar disorder   - Depressive symptoms   - Anxiety symptoms   - Symptoms of obsessive-compulsive disorder   - (Post-)traumatic stress symptoms   - Symptoms of somatoform disorder   - Symptoms of eating disorder (e.g., eating disorder-specific psychopathology)   - Symptoms of substance-related disorders / substance use disorder   - Symptoms of gambling / gaming disorders - Secondary outcomes: general non-disorder-specific mental health outcomes, such as:   - Psychological distress   - General mental health, well-being, or quality of life   - (Perceived) stress   - Sleep problems / sleep quality   - Self-harm, suicidal ideation   - (Perceived) social support, loneliness   Exclusion:   - The missing reporting of the described primary or secondary outcomes was not an exclusion criterion in this review. |
| Study design | Inclusion:   - Quantitative longitudinal survey studies (measuring mental health before versus during the COVID-19 pandemic or at several [peri-pandemic] time points during the pandemic in the same individuals) - Quantitative repeated cross-sectional survey studies (measuring mental health before versus during the COVID-19 pandemic or at several [peri-pandemic] time points during the pandemic in different individuals)   Exclusion:   - Preprints or study protocols - Retrospective studies - Intervention studies - Qualitative survey studies - Theoretical / discussion papers - Narrative reviews / systematic reviews / meta-analyses - Letters to the editor / editorials / commentaries |

*Note.* DSM-5: Diagnostic and Statistical Manual of Mental Disorders (5^th^ edition); ICD-10: International Classification of Diseases (10^th^ edition).
