# Supplementary material for: Mental Health Impact of Early Stages of the COVID-19 Pandemic on Individuals with Pre-Existing Mental Disorders: A Systematic Review of Longitudinal Research
Source: Int J Environ Res Public Health. 2023 Jan 4;20(2):948. doi: 10.3390/ijerph20020948 (PMC9858748; doi:10.3390/ijerph20020948)
Supplement: Supplementary file 1 [file ijerph-20-00948-s001.zip › Supplementary material/Supplementary material file 4.docx]

**Supplementary material file 4. Description of machine learning mechanisms used during title/abstract screening**

For title/abstract screening, two mechanisms of active machine learning (ML) in EPPI-Reviewer were used to accelerate the screening process and to ensure an efficient workflow: i) priority screening to order records for manual title/abstract screening, and ii) building a customized ML classifier to automatically exclude likely-irrelevant references based on already included eligible records. When using priority screening at the level of title/abstract screening, the machine actively and iteratively learns the characteristics of included and excluded studies and is able to predict whether a given record is more likely to be relevant or irrelevant. It results in relevant records being screened in the beginning of the screening process.

To use priority screening, an initial set of relevant (i.e., meeting the eligibility criteria) and irrelevant observational studies was identified from the citations entered into EPPI-Reviewer and manually screened (in ‘normal data entry mode’, i.e., by a single reviewer). The screening mode was changed to ‘comparison data entry mode’ to double-screen titles/abstracts. Priority screening was set up, with a list of records to be screened being automatically generated by EPPI-Reviewer based on the machine’s learning from the manually screened citations. Subsequently, the list of studies generated in the previous step was screened manually by two reviewers working independently. During title/abstract screening, the step of generating this list was re-run, constantly incorporating new screening decisions. This process was repeated until a plateau in the screening plot was reached (see Figure S4.1), indicating that among the unscreened records, no further relevant studies were likely.


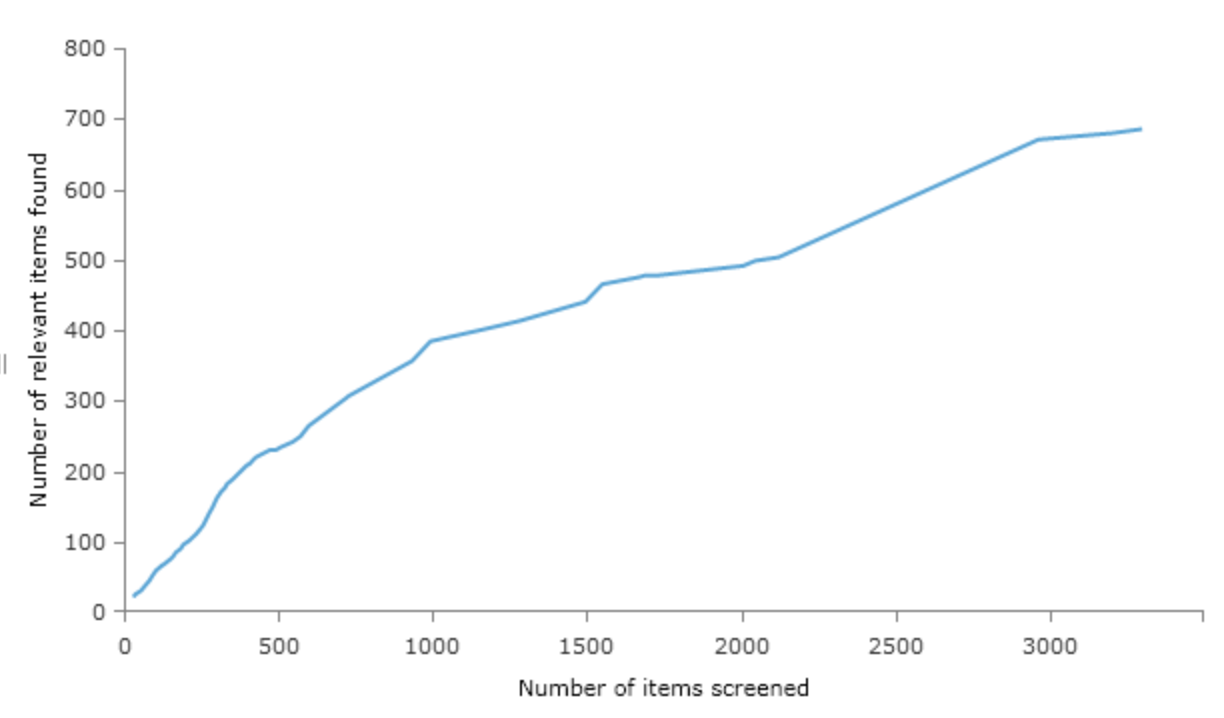


**Figure S4.1.** Screening plot for title/abstract screening in EPPI-Reviewer.

After this plateau was reached during title/abstract screening (i.e., after 3330 records manually screened using priority screening), we built a (customized) ML classifier in EPPI-Reviewer which assigned the 6767 unscreened citations a value indicating the likelihood of being relevant or not. The ML model considered 6259 records to have chance of less than 10% of being relevant (see Figure S4.2). After performing a sensitivity analysis on 150 references within the 5–9% range, that were screened as ineligible by two reviewers, we automatically excluded (i.e., without human screening) all references falling in the 0–9% range (6109 records). The remaining unscreened titles/abstracts (i.e., >10%; 508 records) were manually screened by two independent reviewers, before starting the full-text screening in EPPI-Reviewer.


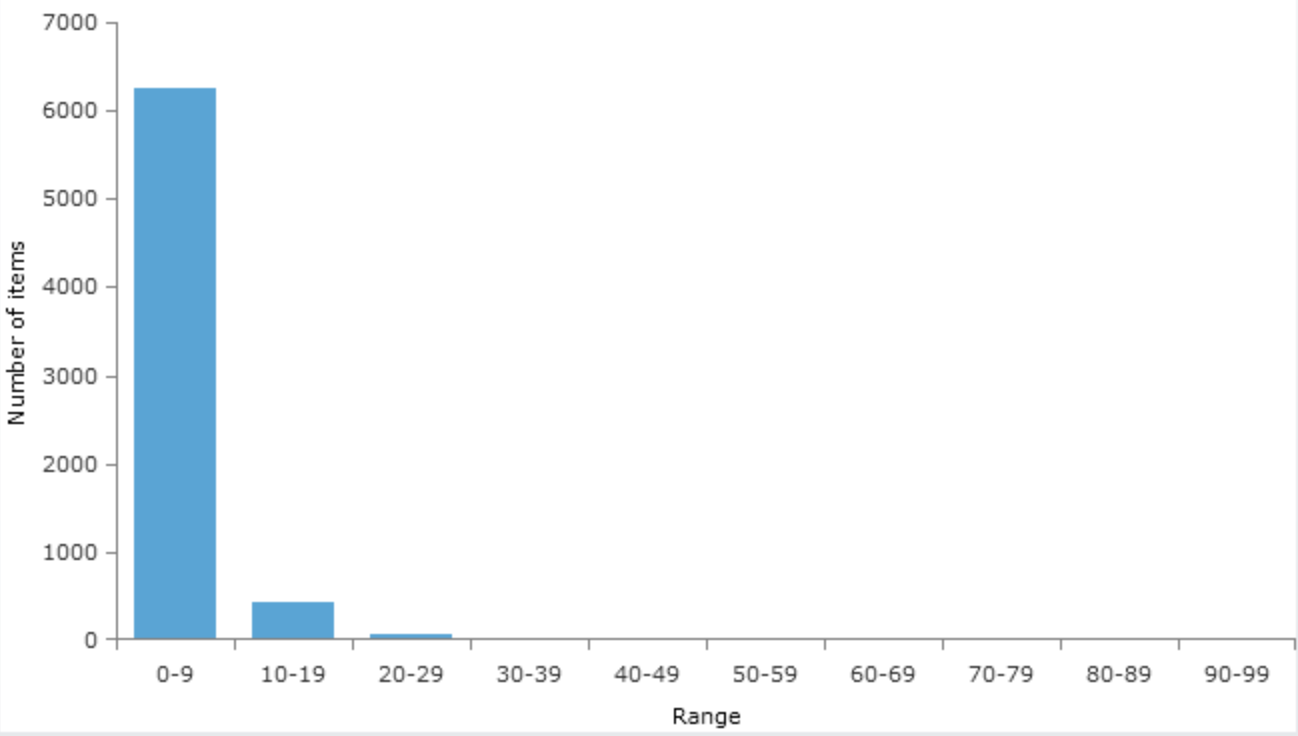


**Figure S4.2.** Distribution of ML classifier scores across unscreened records.
