# Supplementary material for: Mental Health Impact of Early Stages of the COVID-19 Pandemic on Individuals with Pre-Existing Mental Disorders: A Systematic Review of Longitudinal Research
Source: Int J Environ Res Public Health. 2023 Jan 4;20(2):948. doi: 10.3390/ijerph20020948 (PMC9858748; doi:10.3390/ijerph20020948)
Supplement: Supplementary file 1 [file ijerph-20-00948-s001.zip › Supplementary material/Supplementary material file 5.docx]

**Supplementary material file 5. Customized data extraction sheet**

**Table S5.1. Customized data extraction sheet**

| **Category** | **Extracted data** |
| --- | --- |
| Study information | - Study ID - Citation and contact details |
| Eligibility | - Confirm eligibility for review |
| Methods | - Study design - Survey period - Time points of assessment of mental health (i.e., pre- and peri-pandemic assessments or solely peri-pandemic assessments) |
| Participants | - Country - Setting (e.g., inpatient, outpatient setting) - Inclusion/exclusion criteria - Studied sub-population(s) depending on diagnosis of mental disorder - Diagnostic assessment (e.g., clinician-based, self-report) - Previous treatment - Duration of illness - Sample size - Sex (% female) - Age (mean age [*SD*] or alternative data) |
| Outcomes | - Outcome definition - Type of outcome (e.g., continuous, dichotomous) - Assessment tool with coding (e.g., lower is better), scale, and total range - Time points assessed - Cut-off values used for dichotomous outcomes (e.g., prevalence) |
| Results | - For each outcome of interest: - Summary data for each group (e.g., means and *SD*s; median and *IQR*; prevalence rates) - Results of statistical analyses (e.g., *t-*test, analysis of variance; difference in prevalence rates; regression analysis) - Number of participants analyzed - Effect sizes (if available), such as (standardized) mean differences - Effect direction |

*Note.* Detailed extracted data based on this sheet for each study can be requested from the authors.

*Abbreviations:* *IQR*: interquartile range; *SD*: standard deviation.
