# Supplementary material for: Mental Health Impact of Early Stages of the COVID-19 Pandemic on Individuals with Pre-Existing Mental Disorders: A Systematic Review of Longitudinal Research
Source: Int J Environ Res Public Health. 2023 Jan 4;20(2):948. doi: 10.3390/ijerph20020948 (PMC9858748; doi:10.3390/ijerph20020948)
Supplement: Supplementary file 1 [file ijerph-20-00948-s001.zip › Supplementary material/Supplementary material file 6.docx]

**Supplementary material file 6. Adapted quality assessment tool**

**Table S6.1. Adapted quality assessment tool**

| **Item** | **Abbreviation of this item** | **Reason for modification/reason for omission of the item in the table** | **Description of answer options** |
| --- | --- | --- | --- |
| 1. Was the research question or objective in this paper clearly stated?  (original item #1) | 1. Research question clearly defined | not modified/omitted | - Yes: goal in conducting this research described and easy to understand - No: goal in conducting this research not described or not easy to understand |
| 2. Was the study population clearly specified and defined?  (original item #2) | 2. Study population clearly defined | not modified/omitted | - Yes: description of the sample population using demographics, location, and time period - No: no sufficient description |
| Was the participation rate of eligible persons at least 50%?  (original item #3) | omitted | Since several observational studies in the context of the COVID-19 pandemic investigated a subsample of an original study that was recontacted during the pandemic (not necessarily only focused on people with mental illness), the original participation rate was largely unclear. |  |
| 3. Were all the subjects selected or recruited from the same or similar populations (including the same time period)? Were inclusion and exclusion criteria for being in the study pre-specified and applied uniformly to all participants?  (original item #4) | 3. Selection criteria clearly defined | not modified/omitted | - Yes: inclusion and exclusion criteria were developed prior to recruitment or selection of the study population; the same underlying criteria were used for all subjects involved - No: inclusion and exclusion criteria were not developed prior to recruitment or selection of the study population; the underlying criteria were not used in the same way for all subjects involved - Not reported: no in- or exclusion criteria reported |
| 4. Was a sample size justification, power description, or variance and effect estimates (instead of sample size calculations) provided?  (original item #5) | 4. Sample size justification, power description, or variance and effect estimates described | not modified/omitted | - Yes: a sample size justification, power description, or variance and effect estimates (instead of sample size calculations) were calculated and reported - No: a sample size justification, power description, or variance and effect estimates (instead of sample size calculations) were NOT calculated and reported - Not reported: sample size justification, power description, or variance and effect estimates were calculated but not reported   (assessed based on original study & follow-up studies) |
| For the analyses in this paper, were the exposure(s) of interest measured prior to the outcome(s) being measured?  (original item #6) | omitted | Since the exposure was not predictable, this question is not applicable. |  |
| Was the time frame sufficient so that one could reasonably expect to see an association between exposure and outcome if it existed?  (original item #7) | omitted | This question was not applicable as the time frame over which the exposure (i.e., pandemic) must occur until effects on the outcome (i.e., mental health) become apparent is unclear |  |
| 5. For exposures that can vary in amount or level, did the study examine different levels of the exposure as related to the outcome (e.g., categories of exposure, or exposure measured as continuous variable)?  (original item #8) | 5. Exposure clearly specified | This item is based on the assumption of a linear relationship between exposure and effect. Normally, a study receives a better evaluation if it is conducted at several points in time or exposure levels, because the correlation becomes more visible. However, since a linear relationship cannot be assumed for studies included in this review, the item was modified. | - Yes: exposure to the SARS-CoV-2 pandemic was clearly stated, that is, the study was conducted after the first SARS-CoV-2 infection in the respective country (based on WHO data) was publicly reported - No: exposure to the SARS-CoV-2 pandemic was not clearly reported |
| 6. Were the exposure measures (independent variables) clearly defined, valid, reliable, and implemented consistently across all study participants?  (original item #9) | 6. Exposure consistent across all study participants | This question was modified in the sense that the heterogeneity of exposure is assessed, since in the case of the COVID-19 pandemic as exposure, significant differences over a longer period might occur, for example, through loosening or tightening of the initial restrictions or if the number of infections increases or decreases. A smaller survey period is therefore desirable in the sense of a more homogeneous sample. | - Yes: survey period (during COVID-19 pandemic) ≤ 4 weeks - No: survey period (during COVID-19 pandemic) > 4 weeks - Not reported: survey period was not clearly defined |
| 7. Was the exposure assessed more than once over time?  (original item #10) | 7. Exposure assessed more than once over time | Not modified/omitted | Prior to vs. during pandemic:   - Yes: pre-pandemic assessment and ≥ 2 peri-pandemic assessments - No: only pre-pandemic and < 2 peri-pandemic assessments   Peri-pandemic (no pre-pandemic data):   - Yes: ≥ 3 peri-pandemic assessments - No: <3 pre-pandemic assessments |
| 8. Were the outcome measures (dependent variables) clearly defined, valid, reliable, and implemented consistently across all study participants?  (original item #11) | 8. Outcome measures clearly defined, valid, reliable and implemented consistently | Not modified/omitted | - Yes: all outcome measures were clearly defined, valid, reliable, and implemented consistently - Partly: ≥50% of the outcome measures were clearly defined, valid, reliable, and implemented consistently - No: none of the outcome measures were clearly defined, valid, reliable, and implemented consistently - Not reported: no outcome measures were reported |
| Were the outcome assessors blinded to the exposure status of participants?  (original item #12) | omitted | Blinding the studies with the COVID-19 pandemic (exposure) is not possible, that is, the question is not applicable. |  |
| 9. Was loss to follow-up after baseline 20% or less?  (original item #13) | 9. Loss to follow-up ≤20% | Not modified/omitted | - Yes: loss to follow-up after baseline ≤ 20% OR study sample was composed based on those participants for whom baseline data from patient registers were available and there is no loss to follow-up - No: loss to follow-up after baseline > 20% - NR: study does not report the number of participants who possibly dropped out or were excluded during the study (e.g., between two peri-pandemic assessments) - NA: loss to follow-up cannot be assessed due to **repeated cross-sectional study design**   OR  only a subsample from an original study (not necessarily only in people with mental illness) was recontacted and the loss to follow-up with respect to the baseline cannot be adequately assessed and <2 peri-pandemic assessments  (**special case:** for studies investigating a subsample from an original study but reporting at least two peri-pandemic assessments, the loss to follow-up across the peri-pandemic measurements was judged)  (assessed based on original study & follow-up studies) |
| 10. Were key potential confounding variables measured and adjusted statistically for their impact on the relationship between exposure(s) and outcome(s)?  (original item #14) | 10. Confounding variable(s) assessed/adjusted for | Not modified/omitted | - Yes: at least one potential confounding variable was measured and adjusted for in statistical analysis - No: no potential confounding variables were measured and adjusted for in statistical analysis |

*Note.* Adapted tool based on National Institutes of Health (NIH) Quality Assessment Tool for Observational Cohort and Cross-Sectional Studies [1].

**References**

1. National Institutes of Health. Study Quality Assessment Tools. Quality Assessment Tool for Observational Cohort and Cross-Sectional Studies. 2021. Available online: <https://www.nhlbi.nih.gov/health-topics/study-quality-assessment-tools> (accessed on 29 November 2022).
