# Supplementary material for: Mental Health Impact of Early Stages of the COVID-19 Pandemic on Individuals with Pre-Existing Mental Disorders: A Systematic Review of Longitudinal Research
Source: Int J Environ Res Public Health. 2023 Jan 4;20(2):948. doi: 10.3390/ijerph20020948 (PMC9858748; doi:10.3390/ijerph20020948)
Supplement: Supplementary file 1 [file ijerph-20-00948-s001.zip › Supplementary material/Supplementary material file 7.docx]

**Supplementary material file 7. Details on data synthesis methods**

Initially, we had planned to conduct pairwise meta-analyses comparing mental health outcomes prior to versus during the COVID-19 pandemic as well as between multiple peri-pandemic assessments (see PROSPERO CRD42021251770). However, we considered a meta-analytic approach to be inappropriate for this review and decided to use narrative synthesis, specifically vote counting based on the direction of effects [1,3] for several reasons.

First, within each diagnostic group (e.g., anxiety disorders), the available evidence for the pre-specified comparisons was limited (i.e., maximum of five studies measuring a specific mental health outcome such as anxiety symptoms in a diagnostic group). Second, outcomes were in part incompletely reported (e.g., no effect measure reported, but only direction of effect with *p* value or missing variances of effects). Different statistical analyses and effect measures were used across the included studies (e.g., dichotomous outcomes and reporting of prevalence, continuous outcomes analyzed using paired *t-*tests, Wilcoxon test, analysis of variance, multilevel models, or regression analysis). Finally, there were concerns about diversity in the patient populations and outcomes (e.g., differences in previous treatment or duration of mental illness) preventing meta-analyses. Vote counting based on effect directions was preferred over combining *p* values due to the inconsistency in effect measures and data reported across studies. Moreover, directional hypotheses and, thus, one-sided *p* values were not available for all comparisons, especially for peri-pandemic changes in mental health.

To assign studies to one of the two important comparisons, that is, measuring pre- to peri-pandemic or peri-pandemic changes of mental health (or both) we relied on the survey periods stated in publication reports. Specifically, we considered how much time had passed since the first COVID-19 case registered in the respective country, which partly differed from the study authors’ description. For example, Hochstatter et al. [2] reported that their study compared data before (i.e., January 1 to March 12, 2020) and during the pandemic (i.e., March 24 to May 04, 2020). However, since the first case in the United States had occurred on January 20, 2020 (according to the World Health Organization) and the greater part of the first assessment took place afterwards, we assumed the first assessment to be mostly during the pandemic and considered the comparison of mental health in this study as peri-pandemic.

General mental health outcomes (see Additional file 3) and those which can be specific to a mental disorder but are also of general interest (e.g., anxiety symptoms, depressive symptoms) were more frequently reported across various diagnostic groups based on the available evidence and, thus, were considered for vote counting independent of the number of available studies. However, contrary to the protocol describing these variables as primary outcomes of this review, disorder-specific outcomes had to be assessed by at least two studies within a diagnostic group across the two forms of temporal comparisons (i.e., pre- to peri-pandemic or peri-pandemic changes). We chose this approach to avoid that vote counting was based on numerous singular outcomes that have only been measured by individual studies and to make valuable conclusions based on the narrative synthesis. Exceptions were disorder-specific symptoms, which seemed to be of importance of a specific diagnostic group (e.g., post-traumatic stress symptoms in individuals with post-traumatic stress disorder; hypo-maniac symptoms in individuals with bipolar disorder) but were only measured by a very small number of studies. In general, if eligible (longitudinal/repeated cross-sectional) studies additionally reported mental health outcomes assessed retrospectively, the latter were not considered for vote counting. Given the focus of this review, vote counting was limited to mental health outcomes, excluding potentially other reported outcomes (e.g., job satisfaction).

**References**

1. Campbell, M.; McKenzie, J. E.; Sowden, A.; Katikireddi, S. V.; Brennan, S. E.; Ellis, S.; Hartmann-Boyce, J.; Ryan, R.; Shepperd, S.; Thomas, J.; Welch, V.; Thomson, H. Synthesis without meta-analysis (SWiM) in systematic reviews: reporting guideline. *BMJ* **2020***, 368*, l6890. doi:10.1136/bmj.l6890.
2. Hochstatter, K. R.; Akhtar, W. Z.; Dietz, S.; Pe-Romashko, K.; Gustafson, D. H.; Shah, D. V.; Krechel, S.; Liebert, C.; Miller, R.; El-Bassel, N.; Westergaard, R. P. Potential influences of the COVID-19 pandemic on drug use and HIV care among people living with HIV and substance use disorders: Experience from a pilot mHealth intervention. *AIDS Behav* **2021**, *25*, 354–359. doi:10.1007/s10461-020-02976-1.
3. McKenzie, J. E.; Brennan, S. E. Chapter 12: Synthesizing and presenting findings using other methods. In: *Cochrane Handbook for Systematic Reviews of Interventions version 6.3 (updated February 2022)*; Higgins, J. P. T.; Thomas, J.; Chandler, J.; Cumpston, M.; Li, T.; Page, M. J. et al.; Cochrane, 2022. Available online: [www.training.cochrane.org/handbook](http://www.training.cochrane.org/handbook) (accessed 29 November 2022).
