# Supplementary material for: Mental Health Impact of Early Stages of the COVID-19 Pandemic on Individuals with Pre-Existing Mental Disorders: A Systematic Review of Longitudinal Research
Source: Int J Environ Res Public Health. 2023 Jan 4;20(2):948. doi: 10.3390/ijerph20020948 (PMC9858748; doi:10.3390/ijerph20020948)
Supplement: Supplementary file 1 [file ijerph-20-00948-s001.zip › Supplementary material/Supplementary material file 8.docx]

**Supplementary material file 8. Detailed study characteristics**

**Table S8.1. Detailed study characteristics**

| **Study ID** | **Diagnostic**  **groups** | **Country** | **Study design** | **Setting category (setting details)** | **Diagnostic assessment** | **Previous treatment (Y/N) / description / duration of illness** | **Sample size / n(%) female / age (*M* ± *SD*)** | **Assessments** | **Outcomes (tools); bold outcomes were used for vote counting** |
| --- | --- | --- | --- | --- | --- | --- | --- | --- | --- |
| **A. PRE- TO PERI-PANDEMIC CHANGES OF MENTAL HEALTH** | | | | | | | | | |
| Castellini 2020 [1] | ED^1^ | Italy | L | Outpatient (outpatient Clinic for EDs of University of Florence) | C | Yes / pre-lockdown: psychotherapy (Individual Enhanced Cognitive Behavioral Therapy) once per week; during lockdown: online medical examinations and psychotherapy / NR | 74 / 74 (100%) / 31.74 ± 12.76 | P1: 01/2019 - 09/2019  P2: 11/2019 - 01/2020  D1: 22/04/2020 - 03/05/2020 | **Eating disorder specific psychopathology (overall;** stratified by diagnosis AN and BN; **EDE-Q); general psychopathology (overall;** stratified by diagnosis AN and BN; **BSI)**; **objective binge eating monthly** (overall and stratified by AN and BN; NR) |
| Chakraborty 2020 [2] | OCD | India | L | Outpatient (OCD patients who visited department; whose adherence to medication were good and **used to visit outpatient department** in their scheduled time) | NR; C | Yes / NR (patients had visited department and were under continued treatment from the same) / NR | 84 / 64 (76.2%) / NR | P1: NR (pre-pandemic scores; last recorded Y-BOCS severity score noted from the case register)  D1: 23/04/2020 - 22/05/2020 | **OCD severity (Y-BOCS)** |
| Cordellieri 2021 [3] | SSOPD^2^ | Italy | L | Residential care (residential accredited psychiatric facilities) | NR; probably C | Yes / living in psychiatric residential facilities / NR (chronic - residential living) | 22 / 10 (45.5%) / 31.82 ± 6.96 | P1: 11/2019  D1: 04/2020 | Cognitive domains (e.g., orientation to time; MMSE);  patients’ functioning (overall and subscales; psychological impairment; social skills; violence; ADL-occupational skills; substance abuse; compromising of physical conditions: medical impairment; ancillary impairment [legal, financial, milieu]; K Axis with average/global representation of functioning [GAF Eq], global assessment of functioning [GAF K], danger level [DL]);  **psychiatric symptoms (BPRS)** |
| Giel 2021 [4] | ED^3,4^ | Germany | L | NR (IMPULS trial: Department of Psychosomatic Medicine and Psychotherapy, Tübingen, Germany; participants recruited via emails, bulletins, flyers, press releases, outpatient psychotherapists) | C | Yes / psychotherapy in IG participants from IMPULS trial (CBT-oriented group therapy^5^ / duration of illness (from original IMPULS trial 3 years before): IG: 15.9 ± 11.4, CG: 15.5 ± 12.2 | 42 (52% of IMPULS trial sample) / 34 (81%) / 41.1 ± 12.6 (at baseline), 41.3 ± 12.6 (end of treatment), 45.5 ± 12.6 (COVID-19 follow-up) | P1: NR (entering IMPULS trial; IMPULS trial carried out between 03/2015 - 09/2017)  P2: NR (end of treatment/trial)  D1: 05/2020 - 07/2020 | **Depressive symptoms (BDI-II);** **eating disorder specific psychopathology (EDE-Q);** **general psychopathology (SCID)** |
| Goldfarb 2022 [5] | ASD^6^ | Israel | L | Other (employment-related services: employed adults with ASD recruited through organizations offering employment-related services for autistic adults) | C | NR / NR / NR | 23 (completed both surveys) / 4 (17.4%) / 30.22 ± 7.4 | P1: 09/2019 - 01/2020  D1: 04/2020 - 05/2020 | **Psychological (emotional) distress (GHQ-12)** |
| Hamm 2020 [6] | DD^7^ | USA | L | Probably outpatient (participants recruited from both primary care and mental health sites; see [OPTIMUM clinical trial](https://www.sciencedirect.com/science/article/abs/pii/S1064748119303380)) | C | Yes / antidepressant treatment (possibly psychotherapy) / 38.6 years (age at survey M ± SD = 69.2 ± 6.0; age at onset 30.6 ± 18.8) | 73 / 50 (68.5%) /  69.2 ± 6.0 | P1: ~04/2019 ± 9 months **(anxiety symptoms)**^8^**,** ~05/2019 ± 8 months **(depressive symptoms)**^8^  P2: ~12/2019 ± 5 months **(depressive symptoms**)^8^  D1: 01/04/2020 - 23/04/2020 | **Anxiety symptoms (PROMIS-anxiety); depressive symptoms (PHQ-9)** |
| Johnco 2021 [7] | Mixed (anxiety and/or depressive disorder); no subgroup data reported^9^ | Australia | L | NR (NR) | C | Yes / previous treatment with CBT as part of clinical trials / on average 5.67 years post-treatment | 37 / 24 (65%) / 75 ± 5 | P1: ~2009 - 2019 (1 to 129 [M = 68, SD = 43] months prior to COVID-19 lockdown)  D1: 04/2020 - 05/2020 | **Anxiety symptoms (GAI);** **depressive symptoms (GDS);** **psychological distress (K10);** quality of life (physical health, **psychological health**, **social relationships,** environment; **WHOQoL-BREF**) |
| Khosravani 2021 [8] | OCD^10^ | Iran | L | NR (re-evaluation of a cohort of treatment-seeking OCD patients) | C | Yes / 100% pharmacological treatment, 30% previous or current psychotherapeutic treatment including CBT / age of OCD onset: 26.6 ± 8.55; illness duration: 9.6 ± 7.0 years | 270 / 155 (57.4%) / 36 ± 12.1 | P1: NR (before outbreak of COVID-19)  D1: 05/2020 - 07/2020 | **OCD severity (total** and subscales obsessions, compulsions; **Y-BOCS);** OC symptom dimension (total and subscales contamination, responsibility for harm, unacceptable obsessional thoughts, symmetry; DOCS) |
| Kott 2020 [9] | SSOPD^11^ | international / NR | R-CS | NR (data collected from ongoing schizophrenia clinical trials; acute and chronic studies/patients) | NR | NR / NR / NR | NR / NR / NR | P1: NR (before the date of the first confirmed COVID-19 case within each country)  D1: NR (after the date of the first confirmed COVID-19 case within each country) | Active social avoidance, **anxiety,** conceptual disorganization, disorientation, **excitement, hallucinatory behavior,** lack of judgment and insight, stereotyped thinking, tension  (all assessed using **PANSS**) |
| Machado 2020 [10] | ED^12^ | Portugal | L | Probably clinical-acute (currently or previously in treatment at Portuguese specialized hospital unit, University Hospital Center of São João) | NR; probably C since currently or previously in treatment | Yes / 26 (60.5%) currently on ED treatment / NR | 43 / 41 (95.3%) / 27.60 ± 8.45 | P1: NR (last available evaluation before COVID-19 lockdown period)^13^  D1: 30/04/2020 - 15/05/2020 | **Eating disorder psychopathology (EDE-Q)**; emotion regulation difficulties (DERS-SF); impulsivity (UPPS-P); psychosocial impairment (CIA) |
| Matsunaga 2020 [11] | OCD | Japan | L | NR (consecutively treated for more than 3 years in one OCD clinic) | C | Yes / NR (consecutively treated for more than three years in one OCD clinic) / >3 years | 60 (total); **fully remitted persons:** n = 24, **partially remitted persons:** n = 36 / of total sample: 35 (58.3%) / total sample: 41.5 ± 7.9 | P1: NR (before December 2019)  D1: 07/04/2020-02/05/2020 | **OCD severity (Y-BOCS)** separately reported for fully and partially remitted persons |
| Orhan 2021 [12] | BD | Netherlands | L | Probably outpatient (bipolar elderly cohort, patients in contact with services; see [baseline study](https://pubmed.ncbi.nlm.nih.gov/24495405/) in 2017 and 2018) | C | NR / NR / NR (chronic: > 2 years; see recruitment) | 81 / 45 (55.6%) / 66.1 ± 7.2 | P1: 2017 - 2018  D1: 04/2020 | **Anxiety symptoms (BAI); depressive symptoms (CES-D); (hypo-)maniac symptoms (YMRS); loneliness (Loneliness Scale)**; social participation (SPS) |
| Pan 2021 [13] | OCD; DD; AD  (Mixed but subgroup data reported for three different diagnoses and sub-diagnoses along with total patient sample)^14^ | Netherlands | L | NR (recruited from three pre-existing cohort studies: NESDA [recruited from the community, primary care, and specialized mental health care]; NESDO [recruited through specialized mental health-care services], NOCDA [recruited from mental health-care institutions]) | C (before trial start in three cohort studies, respectively) | Yes / in need of mental health treatment: overall: 50/1517 (4%); patients: 48/1181 (4%); current mental health treatment: overall: 605/1517 (44%); patients: 590/1181 (55%)/ NR | Overall: 1517, lifetime mental health disorder: 1181 (77.9%), **panic disorder:** n = 428-481; **generalized anxiety disorder:** n = 413-456; **agoraphobia:** n = 360-404; **social anxiety disorder:** n = 465-523; **major depressive disorder:** n = 852-984;  **dysthymic disorder:** n = 336-373; **obsessive compulsive disorder:**  n = 120-124 (only outcomes anxiety and loneliness assessed in this subgroup) /  Overall: 976 (64%); patients: 791 (67%) / Overall: 56.1 ± 13.2; patients: 55.7 ± 12.9 ^15^ | P1: 2006 - 2016  D1: 01/04/2020 - 13/05/2020 | **Anxiety symptoms (BAI); depressive symptoms (QIDS);** **loneliness (DJGLS);** worry (PSWQ) |
| Peckham 2021 [14] | Mixed (schizophrenia or delusional/ psychotic illness or bipolar disorder), but no subgroup data reported | UK | L | NR (recruited via primary or secondary care; OWLS study participants recruited from 17 mental health trusts [in 6 clinical research networks areas across urban and rural settings in England]; contacted by telephone or letter) | C | NR / NR / NR | 367 / 180 (49%) / 50.5 (SD NR) | P1: 04/2016 - 03/2020  D1: 07/2020-12/2020 | Percentage of individuals smoking tobacco (HIS, derived from Fagerstrom Test for Nicotine Dependence) |
| Pinkham 2020 [15] | SSOPD (Mixed for ‘affective disorder’, i.e., bipolar and depressive disorders, but subgroup data for schizophrenia spectrum reported)^16^ | USA | L | Outpatient (recruited from UCSD, UM, UTD via online advertisement / flyers at outpatient clinics; no participants receiving inpatient care) | C | NR / NR / NR | **Schizophrenia spectrum:** 92; **affective disorders:** 56 / schizophrenia spectrum: 50 (54.3%); affective disorders: 40 (71.4%) / schizophrenia spectrum: 42.95 ± 10.76; affective disorders: 40.77 ± 11.76 | P1: 04/12/2018 - 04/01/2019 (study 1) and 11/07/2019 - 21/07/2019 (study 2); pre-pandemic symptom severity averaged across all completed surveys  D1: 03/04/2020 - 04/06/2020 | **Energized/excited;** happy; **hearing voices;** paranoia; **sad/depressed; sleep (in hours);** **substances used;** **well-being (EMA, respectively)** |
| Rutherford 2021 [16] | PTSD | USA | L | Probably outpatient (pre-existing cohort; data collected from participants in a larger ongoing study examining the influence of chronic PTSD on biological aging processes in older adults) | C | NR / NR (see exclusion criteria: current treatment with mood stabilizers or antipsychotic medications) / chronic PTSD (duration at least 6 months) | 46 / 25 (54.3%) / 62.5 ± 9.0 | P1: before 13/03/2020 ^17^  D1: 01/04/2020 - 08/05/2020 | **Depressive symptoms (HRSD);** cannabis/alcohol use (Drug/Alcohol Followback Assessment); **post-traumatic stress symptoms (PCL-5)** |
| Seitz 2021 [17] | Mixed (PTSD, MDD, SSD; no subgroup data reported) | Germany | L | Mixed inpatient/ outpatient (recruitment through a clinical referral from inpatient and outpatient units as well as via advertisements) | C | Yes / medication at baseline: antidepressants (31.8%), antipsychotics (5.9%), anticonvulsants (1.2%) / NR | 63 / NR: Only provided for full sample (individuals with psychiatric disorders and healthy volunteers) / NR | P1: 09/2018 - 11/2019  D1: 16/04/2020 - 18/05/2020 | **General psychopathology (BSI);** post-traumatic stress symptoms (PCL-5)^19^ |
| Sharma 2021 [18] | OCD | India | R-CS | Outpatient (attended the specialty OCD clinic of a university psychiatric hospital in India for a follow-up visit) | C | Yes / nearly all patients on active pharmacological treatment in both OCD groups (pandemic and historical cohort); previous history of CBT: pandemic cohort: 93 (39%), historical cohort: 60 (33%); CBT practice (yes): 53 (57%), historical cohort: 26 (43.3%) / age at onset of OCD: pandemic cohort: 21.44 ± 8.52; historical cohort: 21.83 ± 8.53; duration of illness: pandemic cohort: 10.92 ± 7.41, historical cohort: 11.14 ± 7.98 | **pandemic cohort:**  240 / 89 (37%) / 32.28 ± 9.70; **historical cohort (data from medical records of independent set of OCD patients followed up during same period 1 year prior):**207 / 72 (34.8%) / 32.97 ± 11.14 | **Historical control cohort:**  P1: NR, baseline assessment (first visit to OCD clinic)  P2: 01/10/2018 – 28/02/2019 (Fu visit in clinic; 1 year prior to FU visit of pandemic cohort)  P3: 04/2019 - 05/2019 (2^nd^ FU visit in clinic; 1 year prior to 2^nd^ FU visit of pandemic cohort)  **Pandemic cohort:**  P1: NR, baseline assessment (first visit to OCD clinic)  P2: 01/10/2019 - 29/02/2020 ^18^ (FU visit in clinic before pandemic)  D1: 26/04/2020 - 12/05/2020 (telephonic FU during pandemic) | **OCD severity (Y-BOCS);** OCD global improvement (CGI-I); OCD symptom severity (CGI-S); proportion of responders, non-responders, remitters (Y-BOCS, CGI-I, CGI-S) |
| Strauss 2022 [19] | SSOPD^20^ | USA | L | Outpatient (recruitment in mental health clinics in Georgia, outpatient setting) | C | Yes / pre-pandemic: 93% taking medications for any psychiatric conditions, 23% with access to remote healthcare (e.g., teletherapy/ video therapy) / NR (chronic) | 32 patients with chronic schizophrenia or schizoaffective disorders / patients: 24 (75%) / patients: 40.13 ± 13.25 | P1: ~08-11/2018 ± 6 months^21^  D1: 07/2020 - 10/2020 | Negative symptoms:  anhedonia, alogia, asociality, avolition, blunted affect, diminished expression, **motivation and pleasure (BNSS)** |
| **B. PERI-PANDEMIC CHANGES OF MENTAL HEALTH** | | | | | | | | | |
| Adams 2021 [20] | ASD | USA | L | NR (pre-existing cohort: Simons Foundation Powering Autism Research for Knowledge [SPARK] registry) | C; inclusion criterion: diagnoses from professional before age of 18 years | Yes / NR / age of autism diagnosis/at onset: 8.71 ± 4.66; duration: 17.74 (since age at survey 26.45 ± 4.66) | 322 (participants consented); 315 completed survey at D1; 275 completed survey at D2; 275 completed both surveys + were analyzed / 135 (49.1%) / 26.45 ± 4.66 | D1: 11/03/2020 - 20/03/2020 D2:18/05/2020 - 27/05/2020 | **Anxiety symptoms (DASS); depressive symptoms (DASS); stress (DASS)^22^** |
| Bal 2021[21] | ASD | USA | L | NR (many participants recruited from university-based autism programs; enrolled in SPARK study: nationwide recruitment with 21 clinical sites and social media strategy) | Self-reported (self-report of a professional ASD diagnosis; many participants are recruited from university‐based autism programs) | Yes / 52.5% receiving mental health services / pre-pandemic: 83.8% with previous mental health diagnosis, 42% with diagnosis before 18 years | 396 (completed both surveys) / 233 (58.8%)  / 37.38 ± 13.36 | D1: 30/03/2020 - 10/04/2020  D2: 27/05/2020 - 06/06/2020 | **Psychological distress (six items from COVID-19 set)^23^;** impact of COVID-19 (number of disrupted areas, total impact: severity for all areas, total coping, “resilient” for seven areas, such as employment; self-developed items) |
| Brondino 2020 [22] | ASD | Italy | L | Daycare (daycare center for adolescents and adults with ASD in Lombardy Region) | C | Yes / 83.3% pharmacotherapy, 66.6% antipsychotics; 27.8% mood stabilizers; physical activity, horticultural therapy, daily cognitive training for language production, augmentative and alternative communication (AAC) use, daily occupational therapy, art therapy (twice a week) / NR | 18 / 5 (27.8%) / 22.72 ± 4.75 | D1: 19/02/2020 ^24^  D2: 04/03/2020 | **Psychiatric symptoms and problem behaviors (total score of irritability, lethargy/social withdrawal, stereotypic behavior, hyperactivity, inappropriate speech; ABC)** |
| Carta 2021 [23] | BD^25^ | Italy, Tunisia | L | Probably outpatient (in care at one of the centers:  Center for Psychiatry of Consultation and Psychosomatics of the University of Cagliari, or Outpatient Clinic of Department Psychiatry A of Razi Hospital La Manouba) | C | Yes / in care at one of the centers named under setting; pharmacotherapy: mood stabilizers (Cagliari: 29 [96.6%], Tunis: 38 [95%]), antipsychotics (Cagliari: 21 [70%], Tunis: 20 [50%]), antidepressants (Cagliari: 1 [3.3%], Tunis: 6 [15%]) / ≥ 1 year (in treatment) | Cagliari (exposed to rigid lockdown): 40 / 28 (70%) / 48.57 ± 11.64;  Tunis (less severe lockdown/not exposed): 30 / 16 (53.3%) / 41.8 ± 13.22 | D1: 04/2020  D2: 06/2020 | **Circadian rhythms (overall** and subscales activity, nutrition/ eating patterns, rhythms/dominant rhythm, sleep, social rhythm; **BRIAN scale**); **depressive symptoms (HAM-D)** |
| Daly 2021 [24] | SSOPD; OCD; ED; BD; DD; AD; PTSD  (Mixed but subgroup data reported)^26^ | USA | L | NR (NR) | Self-reported (patients indicated whether they had been diagnosed by doctor or other healthcare professional) | NR / NR / NR | Of 7319 patients in total sample, 27.5% (n=2013) reporting pre-existing mental health diagnoses; anxiety disorders: 16.2% (n = NR); bipolar disorders: 3.4% (n = NR); depressive disorders: 19.1% (n = NR); eating disorders: 1.9% (n= NR); obsessive compulsive disorders: 3.1% (n = NR); post-traumatic stress disorders: 6.4% (n = NR); schizophrenia/ psychotic disorders:  0.8% (n = NR) / 3755 (51.3%) / 48.9 ± 16.5 ^8^ | D1: 10-18/03/2020  D2: 01-14/04/2020  D3: 15-28/04/2020  D4: 29/04 - 12/05/2020  D5: 13-26/05/2020  D6: 27/05-09/06/2020  D7:10-23/06/2020  D8: 24/06-20/07/2020 | **Psychological distress (PHQ-4)** |
| Davide 2020 [25] | OCD | Italy | L | Outpatient (subjects were outpatients) | C | Yes / attended OCD Clinic at Department of Psychiatry, San Martino University Hospital; psycho-pharmacotherapy with or without CBT / NR | 30 / 16 (53.33%) / 43.17 ± 14.87 | D1: 01/2020 - 02/2020 ^24^  D2: 16-17/04/2020 | **OCD severity (total and subscales obsessions, compulsions; Y-BOCS-SC)** |
| Donati 2021 [26] | GD^27^ | Italy | L | Outpatient (problem gamblers in intermediate to advanced phase of outpatient treatment) | C | Yes / Mostly psychological treatment (78%), psychological + pharmacological (10%), psychological + psychoeducational (4%), psychoeducational + economic tutoring (7%), psychological + pharmacological + psychoeducational + economic tutoring (1%) / 7 years on average (under treatment) | 135 / 26 (19%) / 50.07 ± 13.33 | D1: NR (before lockdown)^24^  D2: 07/04/2020 - 28/05/2020 | **Gambling problem symptoms (total score; modified SOGS)** |
| Gaume 2021 [27] | SUD | Switzerland | L | Mixed inpatient /outpatient (ATC of Lausanne University Hospital; decreased visit frequency when appropriate (i.e., certain patients were given several take-home OAT doses), telehealth services; OAT home delivery for vulnerable patients) | NR; probably C | Yes / OAT program at ATC, Lausanne University Hospital / NR | D1: 49; D2: 51 / D1: 11 (22.9%); D2: 12 (27.3%) / D1: *Mdn* (*IQR*): 39 (32–50); D2: 41 (34–48) | D1: 17-24/04/2020  D2: 04-08/05/2020 | **Use and purchase of heroin/cocaine/cannabis; impact of pandemic on alcohol use/cannabis use/cocaine use/heroin use/use of prescription drugs/other drugs (self-developed single items); impact of pandemic on social and health conditions (social/financial situation, fear of police controls, stealing or racketeering of substances, stress and anxiety, mental health in general, physical health in general; self-developed single items)** |
| Hennigan 2021 [28] | Mixed; various anxiety disorders and OCD; no subgroup data reported)^28^ | Ireland | L | NR (Individuals attending Galway–Roscommon Mental Health Services) | C | Yes / 21 (87.5%) participants of total sample were prescribed psychotropic medication; 10 (41.7%) with prescribed SSRI, 8 (33.3%) with prescribed SNRI; 9 (37.5%) with more than one psychotropic medication / NR | Total (mixed): 24 / 16 (66.7%) / 37.4 ± 11.4 | D1: ~04/2020 (appr. 6 months before assessment 1)^29^  D2: 15/10/2020 - 29/10/2020 | **Anxiety symptoms (BAI; HARS); clinical global impression-severity (CGI-S)^30^; clinical global impression-improvement (CGI-I)^30^; global functioning (GAF);**  **impact of COVID-19 pandemic on anxiety symptoms, mood symptoms, social functioning, occupational functioning, quality of life; Likert scales); OCD severity (total and subscales obsessions, compulsions; Y-BOCS)** |
| Hochstatter 2021 [29] | SUD^31^ | USA | R-CS | Other, mobile health application (platform (A-CHESS) to support people with opioid use disorders (discussion forums, cognitive behavioral therapy, games and relaxation activities, educational information) | NR; probably C since participants were selected for A-CHESS platform | Yes / treatment included in the A-CHESS platform + 5 (8%) receiving medication-assisted treatment / NR | 64 / 16 (25%) / 49 (NR) | D1: 31/01/2020 - 12/03/2020 ^32^  D2: 24/03/2020 - 04/05/2020 | **Alcohol use/marijuana use/other illicit drugs (self-developed single item); confidence rating for staying clean and sober (single item)** |
| Leenaerts 2021 [30] | ED^33^ | Belgium | L | NR (ongoing Belgian Spinning- Out-Of-Control study; participants with recent-onset BN followed during a 1-year-period) | C | Yes / 4 (26.7%) in treatment / maximum illness duration of 5 years (inclusion criterion); *Mdn* = 3 (*IQR*: 2-5) years | 15 / 15 (100%) / *Mdn* (*IQR*) = 23 (4) | D1: 10/01/2020 - 14/03/2020  D2: 19/03/2020 - 09/05/2020 | **Loss of control over their eating behavior/binge eating episode (self-developed items); negative affect (PANAS); positive affect (PANAS)** |
| Lugo-Marin 2021 [31] | ASD | Spain | L | NR (recruited from population monitored within Comprehensive Care Program for Autism Spectrum Disorder (PAITEA), from the Hospital Vall d’Hebron Psychiatry Department) | C | Yes / in part pharmacological treatment (6% reported changes in pharmacotherapy during confinement vs. 94% who reported no changes) / NR (chronic; see recruitment) | 35 / 12 (34.3%) / 32.8 ± 13.1 | D1: NR (pre-lockdown)^34^  D2: NR (post-lockdown; 8 weeks after lockdown onset) | **Anxiety symptoms (SCL-90-R); depressive symptoms (SCL-90-R); hostility (SCL-90-R); interpersonal sensitivity (SCL-90-R); obsessive-compulsive symptoms (SCL-90-R); paranoid ideation (SCL-90-R); phobic anxiety (SCL-90-R); psychological distress (GSI, SCL-90-R); psychoticism (SCL-90-R); somatization (SCL-90-R)** |
| Ma 2020 [32] | SSOPD^35^ | China | L | Clinical-chronic/ Rehabilitation (long-term hospitalization at Wuhan Mental Health Center) | C | Yes / NR (hospitalization) / course of psychosis (years): isolation group: 20.20 ± 9.26 years; long-term hospitalization (length of hospitalization): isolation group: 4.90 ± 2.67 years | 30 / 18 (60%) / 43.17 ± 11.55 | D1: NR (before isolation)^36^  D2: assessments on 10th-14th days of isolation (isolation period for 30 participants:  10/01/2020 - 30/04/2020) | **Anxiety symptoms (HAMA); depressive symptoms (HAMD); psychological stress (CPSS); severity of participants’ psychiatric symptoms (PANSS); sleep quality (PSQI)** |
| Ma 2021 [33] | SSOPD | China | L | Clinical-chronic/ Rehabilitation (long-term hospitalized psychiatric patients) | C | Yes / previous hospital stays of more than 2 years (length of stay: M ± SD = 4.2 ± 3.4 years); 1 antipsychotic drug: n (%) = 5 (23.8%); 2 antipsychotic drugs: n (%) = 16 (76.2%) / course of schizophrenia: M ± SD = 6.8 ± 5.6 years | 21 / 12 (57.1%) / 43.1 ± 2.6 | D1: 01/2020 (patients were uninfected)^36^  D2: NR (within 3 days of diagnosis with COVID-19 after patient was transferred to isolation ward)  D3: NR (after patients were cured; before they were transferred out of isolation ward; transfer of last cured patient on 30/03/2020) | **Psychopathology (total and subscales general psychopathology [GPS], negative symptoms [NSS], positive symptoms [PSS]); PANSS); stress (CPSS)** |
| Nisticò 2021 [34] | ED | Italy | L | Outpatient (Tertiary level outpatient clinic of San Paolo hospital in Milan) | C | Yes / 57 (97%) treated with psychotherapy (before lockdown) / NR | 59 / 57 (97%) / 30.1 ± 12.9 | D1: 25/04/2020 - 28/04/2020  D2: 25/06/2020 - 28/06/2020 | **Anxiety symptoms (DASS-21); depressive symptoms (DASS-21); psychological distress (DASS-21 total score); losing control over food (EDE-Q); restrictive diet (EDE-Q); seeing body (EDE-Q); stress (DASS-21; PSS); subjective distress caused by traumatic events (total and subscales avoidance, intrusion, hyperarousal; IES-R); thinking about body (EDE-Q); well-being (EDE-Q)** |
| Seethaler 2021 [35] | Mixed (affective or anxiety disorders; no subgroup data reported)^37^ | Germany | L | Mixed inpatient/ outpatient (Participants were inpatients or outpatients) | C | Yes / current or former patients of Psychiatric University Hospital of Charité, Germany; mainly pharmacotherapy with antidepressants (13 [40.63%]), 2 (6.26%) without medication / NR | D1: 32; D2: 24 / D1: 20 (62.5%); D2: 16 (66.67%) / D1: 77.94 ± 8.12; D2: 78.25 ± 8.43 | D1: 04/2020 - 05/2020  D2: 08/2020 | **Anxiety (single item); current suicidality (suicidal thoughts, suicidal plans, suicidal attempt; Likert scale); depressive symptoms (GDS-15); other psychopathology (none or present); psychosocial impact of COVID-19 pandemic (self-developed questionnaire); severity of illness (CGI-S)** |
| Wynn 2021 [36] | SSOPD^38^ | USA | L | NR (recruited via VA administrative datasets from VINCI platform and from previous study cohorts) | C; chart diagnoses from VA medical records | NR / NR / NR | 81 / 9 (11.1) / 54.4 ± 9.8 | D1: mid 05/2020 - mid 08/2020 ^39^  D2: mid 08/2020 - mid 10/2020 | **Alcohol use/cannabis use (ASI); anxiety symptoms (GAD-7); depressive symptoms (PHQ-9); fear and distress related to**  **COVID and**  **quarantine orders (FIVE - Adult Report Form); loneliness (ULS); motivation and pleasure (MAP-SR); obsessive-compulsive traits (DOCS); suicidal ideation (MADRS); suspiciousness (RGPTS)** |
| **C. BOTH PRE- TO PERI-PANDEMIC AND PERI-PANDEMIC CHANGES OF MENTAL HEALTH** | | | | | | | | | |
| Liu 2021 [37] | SUD^40^ | China | L | Outpatient (Wuhan First Health Clinic (part of Wuhan Mental Health Center specialized in distributing free methadone to heroin-dependent patients) | C | Yes / registered at the Wuhan First Health Clinic and receiving MMT for > 1 year; years of MMT: 14.20 ± 3.28 / course of drug addiction: 22.32 ± 8.42 years | 76 / 26 (34.2%) / 48.53 ± 5.99 | P1: 10/2019 - 12/2019  D1: 02/2020 - 04/2020 (outbreak)  D2: 05/2020- 06/2020 (post-pandemic) | **Anxiety symptoms (HAMA);** **alcohol consumption (self-developed questionnaire); amphetamine/ morphine use (urine drug test board);** drug craving (VAS); **depressive symptoms (HAMD);**  **stress (PSS);** **tobacco consumption (self-developed questionnaire)**; withdrawal symptoms (COWS) |
| Mergel 2021 (follow-up study);  Schützwohl 2020 (original study reporting two assessments) [38] | Mixed (schizophrenia, affective disorders, anxiety disorders, personality disorders; no subgroup data reported but separate data for patients with chronic and acute mental disorders)^41^ | Germany | L | Group 1 (chronic mental disorders): Residential care (Group 1: "accessed via institutions providing assisted living facilities")  Group 2 (acute mental disorders): Clinical-acute (receiving acute psychiatric/ psychotherapeutic treatment at the time of initial survey; recruited from acute admissions to Department of Psychiatry and Psychotherapy, TU Dresden) | C | Yes / group 1 (chronic): living in assisted living; 24 (88.9%) in treatment for mental health problems at time of initial survey; group 2 (acute): 30 (100%) in psychiatric or psychotherapeutic treatment at time of initial survey / chronicity: group 1 (chronic): 6-10 years: 26.1%, >10 years: 73.9%; acute group (group 2): <2 years: 4%, 2-5 years: 16%, 6-10 years: 24%, >10 years: 56% | total: P1: 174; D1: 132; D2: 106; group 1 (chronic mental disorders; not acutely ill in 4 weeks prior to initial survey): n = 19-27; group 2 (acute mental disorders): n = 26-30 / group 1: 13 (48.1%); group 2: 18 (60.0%) / group 1: 49.7 ± 13.1; group 2: 44.0 ± 11.8 | P1: 08/2019 - 03/2020  D1: 23/03/2020 – 20/04/2020  D2: 22/06/2020 - 19/07/2020;  first two assessments reported in Schützwohl 2020 | **Anxiety symptoms (BSI-18 subscale); depressive symptoms (BSI-18 subscale); inclusion (F-INK subscale); participation (F-INK subscale); perceived impairments in social participation (usual activities of daily life, family and domestic responsibilities, getting things done outside of home, daily tasks and obligations, recreation and leisure, social activities, close personal relationships, sex life, stress and extraordinary strain; IMET); psychological distress (BSI-18, GSI); somatization (BSI-18 subscale)** |
| Riblet 2021 [39] | Mixed (MDD; BAD Type I, Psychotic Disorder, PTSD, PD, agoraphobia, GAD, SAD, OCD, AUD, SUD), but no subgroup data reported^42^ | USA | L | Outpatient (Rural patients with serious mental illness who recently accessed inpatient psychiatric treatment and transitioned to the outpatient setting (between 3 and 5 months before the COVID-19 stay-at-home order) | C | Yes / had been hospitalized on an inpatient mental health unit between October and December 2019 / NR | 11 / 1 (9.1%) / 48.0 ± 17.7 | P1: 10/2019 - 12/2019  P2: 11/2019 - 01/2020  D1: 02/2020 - 03/2020 ^43^  D2: 23/04/2020 - 04/05/2020 | **Hopelessness (BHS); perceived burdensomeness (INQ-15); suicide attempts (Columbia Suicide Severity Rating Scale research version);**  **suicidal ideation (BSS); thwarted belongingness (INQ-15)** |
| Yocum 2021 [40] | BD (Mixed; mostly bipolar disorders)^44^ | USA | L | Mixed inpatient/outpatient (recruited from Prechter Bipolar Longitudinal Cohort; admissions to the psychiatric outpatient and inpatient services, University of Michigan Health System) | C; diagnosis confirmed by in-house physicians (except for ~4%) | NR / NR / NR | 560 (total), 345 (participants with bipolar disorder), 147 (healthy controls) / 381 (68%) of total sample (bipolar + healthy controls), NR for bipolar patients / 49 (NR) of total sample (bipolar + healthy control), NR for bipolar patients | For comparison pre-pandemic vs. peri-pandemic:  P1: 15/03/2015-2019 to 30/05/2015-2019  D1: 15/03/2020 - 30/05/2020  For peri-pandemic trajectories:  D1: 30/04/2020  D2: 14/05/2020  D3: 28/05/2020 | **Anxiety symptoms (GAD-7; pre vs. during and peri-pandemic); degree of change/disruption due to COVID-19 pandemic (change in routines, family income/employment, access to social supports, experiencing pandemic-related stress; CIS; only peri-pandemic); depressive symptoms (PHQ-9; pre vs. during and peri-pandemic);**  **sleep (% taking >30 min to fall asleep, % bad sleep quality, % taking sleep medications, sleep duration in hours/day, PSQI; pre vs. during and peri-pandemic)** |

*Note.* *Abbreviations:* ◇: peri-pandemic changes of mental health; ◆: pre-pandemic vs. peri-pandemic mental health assessment and peri-pandemic changes of mental health; ~: approximately; ABC: Aberrant Behavior Checklist; AD: anxiety disorder; AN: Anorexia nervosa; ASD: autism spectrum disorder; ASI: Addiction Severity Index; ATC: Addiction Treatment Center; BAD: bipolar affective disorder; BAI: Beck Anxiety Inventory; BD: bipolar disorder; BDI‐II: Beck Depression Inventory‐II; BHS: Beck Hopelessness Scale; BN: Bulimia Nervosa; BNSS: Brief Negative Symptom Scale; BPRS: Brief Psychiatric Rating Scale; BRIAN: Biological Rhythms Interview of Assessment in Neuropsychiatry; BSI: Brief Symptom Inventory; BSI-18: Brief Symptom Inventory; BSS: Beck Scale for Suicidal Ideation; C: Clinician-based (based on ICD-10 or DSM-5); CBT: Cognitive Behavioral Therapy; CES‐D: Center for Epidemiologic Studies Depression Scale; CGI-I: Clinical Global Impression – Improvement; CGI-S: Clinical Global Impression – Severity; CIA: Clinical Impairment Assessment; CIS: Coronavirus Impact Scale; COWS: Chinese Opioid Withdrawal Symptom Scale; CPSS Chinese Perceived Stress Scale; D: during COVID-19 assessment (e.g., D1: first during-COVID-19 assessment); DASS(-21): Depression Anxiety Stress Scale-(21 items); DD: depressive disorder; DERS-SF: Difficulties in Emotion Regulation Scale – Short Form; DJGLS: De Jong Gierveld Loneliness Scale; DL: Danger level; DSM-5: Diagnostic and Statistical Manual of Mental Disorders-5; DOCS: Dimensional Obsessive-Compulsive Scale; ED: eating disorder; EDE: Eating Disorder Examination; EDE-Q: Eating Disorder Examination Questionnaire; EMA: Ecological Momentary Assessments; F-INK: Measure of Participation and Social Inclusion for Use in People with a Chronic Mental Disorder; FIVE: Fear of Illness and Virus Evaluation; GAD-7: General Anxiety Disorder; GAI: Geriatric Anxiety Inventory; GAF: Global Assessment of Function; GAF Eq: Global Evaluation Functioning Equivalent; GAF K: Global assessment of functioning; GD: gambling disorder; GDS: Geriatric Depression Scale; GDS-15: German Geriatric Depression Scale 15-item; GHQ-12: General Health Questionnaire–Short Form; GSI: Global Severity Index; HAMA: Hamilton Anxiety Scale; HAMD: Hamilton Depression Scale; HAM-D: Hamilton Rating Scale for Depression; HRSD: Hamilton Rating Scale for Depression; HARS: Hamilton Anxiety Rating Scale; HSI: Heaviness of Smoking Index; ICD-10: International Classification of Diseases; IES-R: Impact of Event Scale-Revised; IMET: Index for the Assessment of Health Impairments; INQ-15: Interpersonal Needs Questionnaire-15; *IQR*: interquartile range; K10: Kessler- 10; K Axis: Kennedy Axis V; L: longitudinal; *M*: mean; MADRS: Montgomery-Åsberg Depression Rating Scale; MAP-SR: Motivation and Pleasure Scale – Self-Report; *Mdn*: median; MDD: major depressive disorder; MMSE: Mini Mental State Examination; MMT: Methadone Maintenance Treatment; NAD: non-affective disorder; NESDA: Netherlands Study of Depression and Anxiety; NESDO: Netherlands Study of Depression in Older Persons; NOCDA: Netherlands Obsessive Compulsive Disorder Association Study; NR: not reported; OAD: other affective disorder; OAT: Opioid Agonist Treatment; OCD: obsessive-compulsive disorder; P: pre-COVID-19 assessment (e.g., P1: first pre-COVID-19 assessment); PANAS: Positive and Negative Affect Schedule; PANSS: Positive and Negative Symptom Scale; PCL-5: Post-traumatic Stress Disorder Checklist; PHQ-4: Patient Health Questionnaire-4; PHQ-9: Patient Health Questionnaire; PIH: Partners in Health Scale; PROMIS-anxiety: Patient Reported Outcomes Measurement Information System-anxiety scale; PSQI Pittsburgh Sleep Quality Index; PSS: Perceived Stress Scale; PSWQ: Penn State Worry Questionnaire; PTSD: post-traumatic stress disorder; QIDS: Quick Inventory of Depressive Symptoms; R-CS: repeated cross-sectional; RGPTS: Revised Paranoid Thoughts Scale; RMDD: recurrent major depression disorder; SAB: schizoaffective bipolar; SCID: Structured Clinical Interview for DSM-5; SCL-90-R: Symptom Checklist 90 Revised; *SD*: standard deviation; SNRI: Serotonin and Norepinephrine Reuptake Inhibitor; SOGS: South Oaks Gambling Screen; SPS: Social Participation Scale; SSOPD: schizophrenia spectrum and other psychotic disorder; SSRI: Selective Serotonin Reuptake Inhibitors; SUD: substance use disorder; TU: Technical University (German: Technische Universität); UCSD: University of California San Diego; ULS: Revised UCLA Loneliness Scale; UM: University of Miami; UPPS-P: Impulsive Behavior Scale-Negative Urgency Subscale; USA: United States of America; UTD: University of Texas at Dallas; VA: Veterans Affairs; VAS: Visual Craving Scale; VINCI: Veteran Affairs Informatics and Computing Infrastructure; WHOQoL- BREF: World Health Organization Quality of Life- BREF; Y-BOCS(-SC): Yale Brown Obsessive Compulsive Scale(-Symptom Checklist; YMRS: Young Mania Rating Scale.

^1^ Current DSM-5 diagnosis of anorexia nervosa (AN) and bulimia nervosa (BN).

^2^ Patients with psychosis.

^3^ Patients with history of Binge Eating disorder (BED) who were previously seeking treatment in the IMPULS trial; at the time of the COVID-19 follow-up survey (data available for *n* = 34), 50% of the participants still had a BED diagnosis.

^4^ Outcome ‘frequency of objective binge eating episodes (EDE)’ measured retrospectively (on recall bias).

^5^ See study protocol for IMPULS trial.

^6^ Not relevant for this review since work-related outcomes and thus not extracted: basic psychological need satisfaction and frustration (work domain), work satisfaction.

^7^ Patients with diagnosis of major depressive disorder (MDD).

^8^ Current assessment: 01/04/2020 to 23/04/2020; days between baseline (P1) and current assessment (D1): depressive symptoms (PHQ-9): M±SD = 335.8 ± 250.6, i.e., approx. 11 months ± 8 months (i.e., 05/2019 ± 8 months); anxiety symptoms (PROMIS-anxiety): M±SD = 369.0 ± 275.8, i.e., approx. 12 months ± 9 months (i.e., 04/2019 ± 9 months); days between immediately pre-pandemic (P2) and current assessment (D1): depressive symptoms (PHQ-9): M±SD = 148.5 ± 143.4, i.e., approx. 5 months ± 5 months (i.e., 12/2019 ± 5 months).

^9^ GAD: 35.1%, MDD: 18.92%, depressive disorder NOS: 13.51%, social phobia: 10.81%, dysthymia: 8.11%, anxiety disorder NOS: 5.41%, panic disorder: 2.70%, SSD: 2.70%; high comorbidity (81% with comorbid anxiety or unipolar depressive disorder diagnosis).

^10^ Treatment-seeking patients with principal diagnosis of OCD; substantial number of patients with comorbid disorder (120 [44.4%]), primarily depressive and anxiety disorders.

^11^ Schizophrenia (acute and with predominantly negative symptoms).

^12^ Bulimia nervosa: 14 (32.6%); anorexia nervosa: 20 (46.5%); binge eating disorder: 2 (4.7%), other specified feeding or eating disorder: 7 (16.3%).

^13^ First COVID-19 case on March 2, 2020; first lockdown in Portugal on March 18, 2020.

^14^ For OCD patients, only outcomes of loneliness and anxiety were assessed.

^15^ Proportion of female participants and age NR for various subgroups with different diagnoses of mental disorders.

^16^ Schizophrenia: 40 (43.5%), schizoaffective: 52 (56.5%); among patients with schizoaffective disorder: bipolar, psychotic features: 34 (60.7%), bipolar, no psychotic features: 21 (37.5%), MDD, with psychotic features: 1 (1.8%).

^17^ Baseline assessment up until 13/03/2020 (reported as pre-COVID-19 pandemic by the authors); first COVID-19 case in the USA registered on January 20, 2020 (according to WHO); however, based on information “before 13/03/2020”, very unclear when first assessment took place.

^18^ First COVID-19 case in India on January 30, 2020.

^19^ Perceived social support and fear and personal impact of COVID-19 not extracted since only assessed at one time point (D1).

^20^Study in outpatients with outpatients with chronic schizophrenia or schizoaffective disorder, individuals at clinical high risk for psychosis (i.e., those meeting criteria for a prodromal syndrome), and matched healthy controls (n = 31).

^21^ Mean interval between pre-pandemic and during pandemic interviews in patients with chronic schizophrenia (M±SD): 698.55 ± 191.80 days.

^22^ Feelings of distress due to the COVID-19 pandemic (two items) not extracted since only assessed at one time point (D2).

^23^ Developed by JHSPH COVID-19 Mental Health Measurement Working Group (items 4-12).

^24^ First COVID-19 case in Italy on January 29, 2020; first ‘lockdown’ in Italy from March 9, 2020, onwards.

^25^ See inclusion criteria: diagnosis of bipolar disorder type I or II in two cohorts (Cagliari, Tunis); bipolar disorder type II: Cagliari: 26 (65%), Tunis: 17 (56.6%).

^26^ Data on people with attention hyperactivity disorder and ‘other mental health condition’ also reported in publication but not included in this Table since not important for this review or too unspecific.

^27^ About 40% of patients with comorbidities (especially depressive disorders and personality disorders according to DSM-5).

^28^ ‘Trigger anxiety disorders’ according to publication: OCD: 11 (45.8%), social phobia: 2 (8.3%), agoraphobia: 1 (4.2%); ‘Non-trigger anxiety disorders’: generalized anxiety disorder: 6 (25.0%), mixed anxiety and depression: 3 (12.5%), panic disorder: 1 (4.2%).

^29^ First COVID-19 case in Ireland on March 1, 2020.

^30^ Data on global impression of severity (CGI-S) and combined values for CGI-I measured but not reported in publication.

^31^ People Living with HIV and substance use disorders at high risk for treatment failure at HIV clinics; 70% ever diagnosed with mental health disorder other than SUD.

^32^ See publication: First COVID-19 case in Wisconsin on February 5, 2020; first COVID-19 case in the USA on January 20, 2020.

^33^ Diagnosis of bulimia nervosa; comorbidities: MDD: 6.7% (1); social anxiety disorder 6.7% (1); post-traumatic stress disorder 13.3% (2).

^34^ First COVID-19 case in Spain on January 20, 2020; first lockdown in Spain declared on March 14, 2020.

^35^ Schizophrenic patients subjected to social isolation after close contact with COVID-19 patients.

^36^ First COVID-19 case in China on January 4, 2020; isolation wards officially established on January 30, 2020.

^37^ Main diagnosis (N (%)): depressive episode (F32): 5 (15.63%); recurrent depressive disorder (F33): 20 (62.5%); anxiety disorder (F4): 3 (9.38%); BAD (F31): 3 (9.38%); persistent affective disorder (F34): 1 (3.13%).

^38^ Psychotic disorder diagnosis (other than substance-induced psychosis), such as schizophrenia (n = 42), schizoaffective disorder (n= 22), depressive disorder with psychotic features (n = 1), bipolar disorder with psychotic features (n = 9), or psychotic disorder not otherwise specified.

^39^ Retrospective assessment of pre-pandemic situation (participants asked to give ratings for January 2020), as reported in this study, not considered for this review.

^40^ Heroin-dependent patients undergoing MMT.

^41^ German adults with chronic mental disorders (group 1), acute mental disorders (group 2) and without a mental disorder (group 3; general public; n = 45-49); F2 Schizophrenia et al. (group 1: 10 [38.5%], group 2: 1 [3.6%]); F3 Affective disorders (group 1: 11 [42.3%], group 2: 21 [75.0%]), F4 Anxiety disorders et al. (group 1: 7 [26.9%], group 2: 11 [39.3%]), F6 Personality disorders (group 1: 5 [19.2%], group 2: 2 [7.1%]).

^42^ Baseline diagnoses (October to December 2019): MDD (current): 45.5%; BAD type I (current): 45.5.%; psychotic Disorder (current): 9.1%; PTSD (current): 72.7%; PD (current): 54.5%; agoraphobia: 72.7%; GAD (current): 63.6%; SAD (current): 45.5%; OCD (current): 54.5%; AUD (past 12 months): 45.5.%; SUD (past 12 months): 27.3%.

^43^ First COVID-19 case in the USA on January 20, 2020.

^44^ Study assigned to subgroup of patients with bipolar disorder (all bipolar diagnoses including Bipolar I, Bipolar II, Bipolar, NOS, Schizoaffective Bipolar were collapsed into the category of bipolar disease; n = 345); overall: participants diagnosed with Bipolar I (41%), Bipolar II (14%), Bipolar NOS (5%), Recurrent Major Depression Disorder (2%), Schizoaffective Bipolar (2%), Non-Affective Disorder (3%) or Other Affective Disorder (4%); 26% healthy controls; ~4% did not yet have confirmed diagnosis category.

**References**

1. Castellini, G.; Cassioli, E.; Rossi, E.; Innocenti, M.; Gironi, V.; Sanfilippo, G.; Felciai, F.; Monteleone, A.M.; Ricca, V. The impact of COVID-19 epidemic on eating disorders: A longitudinal observation of pre versus post psychopathological features in a sample of patients with eating disorders and a group of healthy controls. *Int J Eat Disord* **2020**, *53*, 1855-1862, doi:10.1002/eat.23368.
2. Chakraborty, A.; Karmakar, S. Impact of COVID-19 on Obsessive Compulsive Disorder (OCD). *Iranian Journal of Psychiatry* **2020**, *15*, 256-259, doi:10.18502/ijps.v15i3.3820.
3. Cordellieri, P.; Barchielli, B.; Masci, V.; Viani, F.; de Pinto, I.; Priori, A.; Torriccelli, F.D.; Cosmo, C.; Ferracuti, S.; Giannini, A.M.; et al. Psychological health status of psychiatric patients living in treatment communities before and during the COVID-19 lockdown: A brief report. *Int J Environ Res Public Health* **2021**, *18*, doi:10.3390/ijerph18073567.
4. Giel, K.E.; Schurr, M.; Zipfel, S.; Junne, F.; Schag, K. Eating behaviour and symptom trajectories in patients with a history of binge eating disorder during COVID-19 pandemic. *Eur Eat Disord Rev* **2021**, *29*, 657-662, doi:10.1002/erv.2837.
5. Goldfarb, Y.; Gal, E.; Golan, O. I Implications of employment changes caused by COVID-19 on mental health and work-related pychological need satisfaction of Autistic employees: A mixed-methods longitudinal study. *J Autism Dev Disord* **2022**, *52*, 89-102, doi:10.1007/s10803-021-04902-3.
6. Hamm, M.E.; Brown, P.J.; Karp, J.F.; Lenard, E.; Cameron, F.; Dawdani, A.; Lavretsky, H.; Miller, J.P.; Mulsant, B.H.; Pham, V.T.; et al. xperiences of American older adults with pre-existing depression during the beginnings of the COVID-19 pandemic: A multicity, mixed-methods study. *Am J Geriatr Psychiatry* **2020**, *28*, 924-932, doi:10.1016/j.jagp.2020.06.013.
7. Johnco, C.J.; Chen, J.T.H.; Muir, C.; Strutt, P.; Dawes, P.; Siette, J.; Dias, C.B.; Hillebrandt, H.; Maurice, O.; Wuthrich, V.M. Long-term relapse rates after cognitive behaviour therapy for anxiety and depressive disorders among older adults: A follow-up study during COVID-19. *Australas J Ageing* **2021**, *40*, 208-212, doi:10.1111/ajag.12928.
8. Khosravani, V.; Aardema, F.; Samimi Ardestani, S.M.; Sharifi Bastan, F. The impact of the coronavirus pandemic on specific symptom dimensions and severity in OCD: A comparison before and during COVID-19 in the context of stress responses. *J Obsessive Compuls Relat Disord* **2021**, *29*, 100626, doi:10.1016/j.jocrd.2021.100626.
9. Kott, A.; Daniel, D.G. P.508 COVID-19 impact on entry symptom severity in schizophrenia clinical trials – preliminary data. *European Neuropsychopharmacology* **2020**, *40*, S286-S287, doi:10.1016/j.euroneuro.2020.09.372.
10. Machado, P.P.P.; Pinto-Bastos, A.; Ramos, R.; Rodrigues, T.F.; Louro, E.; Gonçalves, S.; Brandão, I.; Vaz, A. Impact of COVID-19 lockdown measures on a cohort of eating disorders patients. *J Eat Disord* **2020**, *8*, 57, doi:10.1186/s40337-020-00340-1.
11. Matsunaga, H.; Mukai, K.; Yamanishi, K. Acute impact of COVID-19 pandemic on phenomenological features in fully or partially remitted patients with obsessive-compulsive disorder. *Psychiatry Clin Neurosci* **2020**, *74*, 565-566, doi:10.1111/pcn.13119.
12. Orhan, M.; Korten, N.; Paans, N.; de Walle, B.; Kupka, R.; van Oppen, P.; Kok, A.; Sonnenberg, C.; Schouws, S.; Dols, A. Psychiatric symptoms during the COVID-19 outbreak in older adults with bipolar disorder. *Int J Geriatr Psychiatry* **2021**, *36*, 892-900, doi:10.1002/gps.5489.
13. Pan, K.Y.; Kok, A.A.L.; Eikelenboom, M.; Horsfall, M.; Jörg, F.; Luteijn, R.A.; Rhebergen, D.; Oppen, P.V.; Giltay, E.J.; Penninx, B. The mental health impact of the COVID-19 pandemic on people with and without depressive, anxiety, or obsessive-compulsive disorders: a longitudinal study of three Dutch case-control cohorts. *Lancet Psychiatry* **2021**, *8*, 121-129, doi:10.1016/s2215-0366(20)30491-0.
14. Peckham, E.; Allgar, V.; Crosland, S.; Heron, P.; Johnston, G.; Newbronner, E.; Ratschen, E.; Spanakis, P.; Wadman, R.; Walker, L.; et al. Investigating smoking and nicotine dependence among people with severe mental illness during the COVID-19 pandemic: analysis of linked data from a UK Closing the Gap cohort. *BJPsych Open* **2021**, *7*, e86, doi:10.1192/bjo.2021.45.
15. Pinkham, A.E.; Ackerman, R.A.; Depp, C.A.; Harvey, P.D.; Moore, R.C. A longitudinal investigation of the effects of the COVID-19 pandemic on the mental health of individuals with pre-existing severe mental illnesses. *Psychiatry Res* **2020**, *294*, 113493, doi:10.1016/j.psychres.2020.113493.
16. Rutherford, B.R.; Choi, C.J.; Chrisanthopolous, M.; Salzman, C.; Zhu, C.; Montes-Garcia, C.; Liu, Y.; Brown, P.J.; Yehuda, R.; Flory, J.; et al. The COVID-19 pandemic as a traumatic stressor: Mental health responses of older adults with chronic PTSD. *Am J Geriatr Psychiatry* **2021**, *29*, 105-114, doi:10.1016/j.jagp.2020.10.010.
17. Seitz, K.I.; Bertsch, K.; Herpertz, S.C. A prospective study of mental health during the COVID-19 pandemic in childhood trauma-exposed individuals: Social support matters. *J Trauma Stress* **2021**, *34*, 477-486, doi:10.1002/jts.22660.
18. Sharma, L.P.; Balachander, S.; Thamby, A.; Bhattacharya, M.; Kishore, C.; Shanbhag, V.; Sekharan, J.T.; Narayanaswamy, J.C.; Arumugham, S.S.; Reddy, J.Y.C. Impact of the COVID-19 pandemic on the short-term course of obsessive-compulsive disorder. *J Nerv Ment Dis* **2021**, *209*, 256-264, doi:10.1097/nmd.0000000000001318.
19. Strauss, G.P.; Macdonald, K.I.; Ruiz, I.; Raugh, I.M.; Bartolomeo, L.A.; James, S.H. The impact of the COVID-19 pandemic on negative symptoms in individuals at clinical high-risk for psychosis and outpatients with chronic schizophrenia. *Eur Arch Psychiatry Clin Neurosci* **2022**, *272*, 17-27, doi:10.1007/s00406-021-01260-0.
20. Adams, R.E.; Zheng, S.; Taylor, J.L.; Bishop, S.L. Ten weeks in: COVID-19-related distress in adults with autism spectrum disorder. Autism 2021, 25, 2140-2145, doi:10.1177/13623613211005919.
21. Bal, V.H.; Wilkinson, E.; White, L.C.; Law, J.K.; Feliciano, P.; Chung, W.K. Early pandemic experiences of autistic adults: Predictors of psychological distress. *Autism Res* **2021**, *14*, 1209-1219, doi:10.1002/aur.2480.
22. Brondino, N.; Damiani, S.; Politi, P. Effective strategies for managing COVID-19 emergency restrictions for adults with severe ASD in a daycare center in Italy. *Brain Sci* **2020**, *10*, doi:10.3390/brainsci10070436.
23. Carta, M.G.; Ouali, U.; Perra, A.; Ben Cheikh Ahmed, A.; Boe, L.; Aissa, A.; Lorrai, S.; Cossu, G.; Aresti, A.; Preti, A.; et al. Living with bipolar disorder in the time of Covid-19: Biorhythms during the severe lockdown in Cagliari, Italy, and the moderate lockdown in Tunis, Tunisia. *Front Psychiatry* **2021**, *12*, 634765, doi:10.3389/fpsyt.2021.634765.
24. Daly, M.; Robinson, E. Psychological distress and adaptation to the COVID-19 crisis in the United States. *J Psychiatr Res* **2021**, *136*, 603-609, doi:10.1016/j.jpsychires.2020.10.035.
25. Davide, P.; Andrea, P.; Martina, O.; Andrea, E.; Davide, D.; Mario, A. The impact of the COVID-19 pandemic on patients with OCD: Effects of contamination symptoms and remission state before the quarantine in a preliminary naturalistic study. *Psychiatry Res* **2020**, *291*, 113213, doi:10.1016/j.psychres.2020.113213.
26. Donati, M.A.; Cabrini, S.; Capitanucci, D.; Primi, C.; Smaniotto, R.; Avanzi, M.; Quadrelli, E.; Bielli, G.; Casini, A.; Roaro, A. Being a gambler during the COVID-19 pandemic: A study with Italian patients and the effects of reduced exposition. *Int J Environ Res Public Health* **2021**, *18*, doi:10.3390/ijerph18020424.
27. Gaume, J.; Schmutz, E.; Daeppen, J.B.; Zobel, F. Evolution of the illegal substances market and substance users' social situation and health during the COVID-19 pandemic. *Int J Environ Res Public Health* **2021**, *18*, doi:10.3390/ijerph18094960.
28. Hennigan, K.; McGovern, M.; Plunkett, R.; Costello, S.; McDonald, C.; Hallahan, B. A longitudinal evaluation of the impact of the COVID-19 pandemic on patients with pre-existing anxiety disorders. *Ir J Psychol Med* **2021**, *38*, 258-265, doi:10.1017/ipm.2021.32.
29. Hochstatter, K.R.; Akhtar, W.Z.; Dietz, S.; Pe-Romashko, K.; Gustafson, D.H.; Shah, D.V.; Krechel, S.; Liebert, C.; Miller, R.; El-Bassel, N.; et al. Potential influences of the COVID-19 pandemic on drug use and HIV care among people living with HIV and substance use disorders: experience from a pilot mHealth intervention. *AIDS Behav* **2021**, *25*, 354-359, doi:10.1007/s10461-020-02976-1.
30. Leenaerts, N.; Vaessen, T.; Ceccarini, J.; Vrieze, E. How COVID-19 lockdown measures could impact patients with bulimia nervosa: Exploratory results from an ongoing experience sampling method study. *Eat Behav* **2021**, *41*, 101505, doi:10.1016/j.eatbeh.2021.101505.
31. Lugo-Marín, J.; Gisbert-Gustemps, L.; Setien-Ramos, I.; Español-Martín, G.; Ibañez-Jimenez, P.; Forner-Puntonet, M.; Arteaga-Henríquez, G.; Soriano-Día, A.; Duque-Yemail, J.D.; Ramos-Quiroga, J.A. COVID-19 pandemic effects in people with Autism Spectrum Disorder and their caregivers: Evaluation of social distancing and lockdown impact on mental health and general status. *Res Autism Spectr Disord* **2021**, *83*, 101757, doi:10.1016/j.rasd.2021.101757.
32. Ma, J.; Hua, T.; Zeng, K.; Zhong, B.; Wang, G.; Liu, X. Influence of social isolation caused by coronavirus disease 2019 (COVID-19) on the psychological characteristics of hospitalized schizophrenia patients: a case-control study. *Translational Psychiatry* **2020**, *10*, 411, doi:10.1038/s41398-020-01098-5.
33. Ma, J.; Jiang, T.; Huang, H.; Li, R.; Zhang, L.; Liu, L.; Liu, X. Mental symptoms and stress of hospitalized schizophrenia patients with 2019 novel coronavirus disease: An observation study. *Front Psychiatry* **2021**, *12*, 557611, doi:10.3389/fpsyt.2021.557611.
34. Nisticò, V.; Bertelli, S.; Tedesco, R.; Anselmetti, S.; Priori, A.; Gambini, O.; Demartini, B. The psychological impact of COVID-19-related lockdown measures among a sample of Italian patients with eating disorders: a preliminary longitudinal study. *Eat Weight Disord* **2021**, *26*, 2771-2777, doi:10.1007/s40519-021-01137-0.
35. Seethaler, M.; Just, S.; Stötzner, P.; Bermpohl, F.; Brandl, E.J. Psychosocial Impact of COVID-19 pandemic in elderly psychiatric patients: a longitudinal study. *Psychiatr Q* **2021**, *92*, 1439-1457, doi:10.1007/s11126-021-09917-8.
36. Wynn, J.K.; McCleery, A.; Novacek, D.; Reavis, E.A.; Tsai, J.; Green, M.F. Clinical and functional effects of the COVID-19 pandemic and social distancing on vulnerable veterans with psychosis or recent homelessness. *J Psychiatr Res* **2021**, *138*, 42-49, doi:10.1016/j.jpsychires.2021.03.051.
37. Liu, X.; Jin, X.; Zhang, Y.; Zhang, L.; Li, Y.; Ma, J. Effect of coronavirus disease 2019 on the psychology and behavior of patients on methadone maintenance treatment in Wuhan, China: A clinical observational study. *Front Psychiatry* **2021**, *12*, 653662, doi:10.3389/fpsyt.2021.653662.
38. Mergel, E.; Schützwohl, M. A longitudinal study on the COVID-19 pandemic and its divergent effects on social participation and mental health across different study groups with and without mental disorders. *Soc Psychiatry Psychiatr Epidemiol* **2021**, *56*, 1459-1468, doi:10.1007/s00127-021-02025-9.
39. Riblet, N.B.; Stevens, S.P.; Shiner, B.; Cornelius, S.; Forehand, J.; Scott, R.C.; Watts, B.V. Longitudinal examination of COVID-19 public health measures on mental health for rural patients with serious mental illness. *Mil Med* **2021**, *186*, e956-e961, doi:10.1093/milmed/usaa559.
40. Yocum, A.K.; Zhai, Y.; McInnis, M.G.; Han, P. Covid-19 pandemic and lockdown impacts: A description in a longitudinal study of bipolar disorder. *J Affect Disord* **2021**, *282*, 1226-1233, doi:10.1016/j.jad.2021.01.028.
