# Supplementary material for: Mental Health Impact of Early Stages of the COVID-19 Pandemic on Individuals with Pre-Existing Mental Disorders: A Systematic Review of Longitudinal Research
Source: Int J Environ Res Public Health. 2023 Jan 4;20(2):948. doi: 10.3390/ijerph20020948 (PMC9858748; doi:10.3390/ijerph20020948)
Supplement: Supplementary file 1 [file ijerph-20-00948-s001.zip › Supplementary material/Supplementary material file 9.docx]

**Supplementary material file 9. Quality assessment of included studies**

**Table S9.1.** Quality assessment of included studies.

|  | **1. Research question clearly defined** | **2. Study population clearly defined** | **3. Selection criteria clearly defined** | **4. Sample size justification, power description, or variance and effect estimates described** | **5. Exposure clearly specified** | **6. Exposure consistent across all study participants** | **7. Exposure assessed more than once over time** | **8. Outcome measures clearly defined, valid, reliable and implemented consistently** | **9. Loss to follow-up ≤20%** | **10. Confounding variable(s) assessed/adjusted for** | |
| --- | --- | --- | --- | --- | --- | --- | --- | --- | --- | --- | --- |
| Adams 2021 [1] | Y | Y | Y | N | Y | Y | N | Y | Y^5^ | | Y |
| Bal 2021 [2] | Y | Y | Y | N | Y | Y | N | N | N^5^ | | Y |
| Brondino 2020 [3] | Y | Y | Y | N | Y | Y | N | Y | Y | | N |
| Carta 2021 [4] | Y | Y | Y | N | Y | Y | N | Y | NR | | N |
| Castellini 2020 [5] | Y | Y | Y | N | Y | Y | N | Y | Y | | Y |
| Chakraborty 2020 [6] | Y | Y | Y | N | Y | Y | N | Y | Y | | N |
| Cordellieri 2021 [7] | Y | Y | Y | N | Y | Y | N | Y | NR | | N |
| Daly 2021 [8] | Y | Y | Y | N | Y | Y | Y | Y | Y | | Y |
| Davide 2020 [9] | Y | Y | Y | N | Y | Y | N | Y | NR | | Y |
| Donati 2021 [10] | Y | N | N | N | Y | N | N | Y | Y | | N |
| Gaume 2021 [11] | Y | Y | Y | N | Y | Y | N | N | Y^6^ | | Y |
| Giel 2021 [12] | Y | Y | Y | Y^2^ | Y | N^3^ | N | Y | N | | N |
| Goldfarb 2022 [13] | Y | Y | Y | N | Y | N^3^ | N | Y | N | | N |
| Hamm 2020 [14] | Y | Y | Y | Y^2^ | Y | Y | N | Y | N | | N |
| Hennigan 2021 [15] | Y | Y | Y | N | Y | Y | N | Partly | Y | | N |
| Hochstatter 2021 [16] | Y | N | N | N | Y | N | N | N | NA (R-CS) | | Y |
| Johnco 2021 [17] | Y | Y | Y | N | Y | N^3^ | N | Y | NR | | N |
| Khosravani 2021 [18] | Y | Y | Y | N | Y | N | N | Y | N | | N |
| Kott 2020 [19] | Y | N | NR^1^ | N | Y | NR^1^ | N | Y | NA (R-CS) | | N |
| Leenaerts 2021 [20] | Y | Y | Y | N | Y | N | N | Partly | NR | | Y |
| Liu 2021 [21] | Y | Y | Y | N | Y | N^3^ | Y | Partly | NR | | N |
| Lugo-Marín 2021 [22] | Y | N | Y | Y | Y | NR | N | Y | Y | | N |
| Ma 2020 [23] | Y | Y | Y | N | Y | Y | N | Y | NR | | N |
| Ma 2021 [24] | Y | Y | Y | N | Y | Y | Y | Y | NR | | Y |
| Machado 2020 [25] | Y | Y | Y | N | Y | Y | N | Y | NA | | N |
| Matsunaga 2020 [26] | Y | Y | Y | N | Y | Y | N | Y | NR | | N |
| Mergel 2021 [27] | Y | Y | Y | N | Y | Y | Y | Y | N ^5^ | | N |
| Nisticò 2021 [28] | Y | Y | Y | N | Y | Y | N | Y | N | | N |
| Orhan 2021 [29] | Y | Y | Y | N | Y | Y | N | Y | N | | Y |
| Pan 2021 [30] | Y | Y | Y | Y^2^ | Y | N | N | Y | NA | | Y |
| Peckham 2021 [31] | Y | Y | Y | N | Y | N | N | Y | NA | | N |
| Pinkham 2020 [32] | Y | Y | Y | N | Y | N | N | N | NR | | Y |
| Riblet 2021 [33] | Y | Y | Y | N | Y | Y | Y | Y | Y | | N |
| Rutherford 2021 [34] | Y | Y | Y | N | Y | N | N | Y | NR | | Y |
| Seethaler 2021 [35] | Y | Y | Y | N | Y | N | N | Y | N | | N |
| Seitz 2021 [36] | Y | Y | Y | Y | Y | Y^4^ | N | Y | NA | | Y |
| Sharma 2021 [37] | Y | Y | Y | Y | Y | Y | N | Y | NA (R-CS) | | Y |
| Strauss 2022 [38] | Y | Y | Y | N | Y | N | N | Y | NA | | N |
| Wynn 2021 [39] | Y | Y | Y | N | Y | N | N | Y | Y | | Y |
| Yocum 2021 [40] | Y | Y | Y | N | Y | Y | Y | Partly | N | | Y |

*Note.* Based on adapted NIH Quality Assessment Tool for Observational Cohort and Cross-Sectional Studies.

^1^ Only abstract available.
^2^ Sample size calculation was performed for the original study.
^3^ Survey period only indicated based on months/year and exact dates are missing; for these studies, the survey period was assumed as the whole period throughout the months mentioned.
^4^ Survey period of 32 days (i.e., four weeks are only narrowly exceeded).
^5^ Study investigating a subsample from an original study but reporting ≥ 2 peri-pandemic assessments and the loss to follow-up across the peri-pandemic measurements was judged.

^6^ Gaume, Schmutz, Daeppen and Zobel [81]: No loss to follow-up since n = 49 individuals participated in wave 1 and n = 51 in wave 2; participation had been offered to 70 patients at wave 1.

**References**

1. Adams, R.E.; Zheng, S.; Taylor, J.L.; Bishop, S.L. Ten weeks in: COVID-19-related distress in adults with autism spectrum disorder. Autism 2021, 25, 2140-2145, doi:10.1177/13623613211005919.
2. Bal, V.H.; Wilkinson, E.; White, L.C.; Law, J.K.; Feliciano, P.; Chung, W.K. Early pandemic experiences of autistic adults: Predictors of psychological distress. *Autism Res* **2021**, *14*, 1209-1219, doi:10.1002/aur.2480.
3. Brondino, N.; Damiani, S.; Politi, P. Effective strategies for managing COVID-19 emergency restrictions for adults with severe ASD in a daycare center in Italy. *Brain Sci* **2020**, *10*, doi:10.3390/brainsci10070436.
4. Carta, M.G.; Ouali, U.; Perra, A.; Ben Cheikh Ahmed, A.; Boe, L.; Aissa, A.; Lorrai, S.; Cossu, G.; Aresti, A.; Preti, A.; et al. Living with bipolar disorder in the time of Covid-19: Biorhythms during the severe lockdown in Cagliari, Italy, and the moderate lockdown in Tunis, Tunisia. *Front Psychiatry* **2021**, *12*, 634765, doi:10.3389/fpsyt.2021.634765.
5. Castellini, G.; Cassioli, E.; Rossi, E.; Innocenti, M.; Gironi, V.; Sanfilippo, G.; Felciai, F.; Monteleone, A.M.; Ricca, V. The impact of COVID-19 epidemic on eating disorders: A longitudinal observation of pre versus post psychopathological features in a sample of patients with eating disorders and a group of healthy controls. *Int J Eat Disord* **2020**, *53*, 1855-1862, doi:10.1002/eat.23368.
6. Chakraborty, A.; Karmakar, S. Impact of COVID-19 on Obsessive Compulsive Disorder (OCD). *Iranian Journal of Psychiatry* **2020**, *15*, 256-259, doi:10.18502/ijps.v15i3.3820.
7. Cordellieri, P.; Barchielli, B.; Masci, V.; Viani, F.; de Pinto, I.; Priori, A.; Torriccelli, F.D.; Cosmo, C.; Ferracuti, S.; Giannini, A.M.; et al. Psychological health status of psychiatric patients living in treatment communities before and during the COVID-19 lockdown: A brief report. *Int J Environ Res Public Health* **2021**, *18*, doi:10.3390/ijerph18073567.
8. Daly, M.; Robinson, E. Psychological distress and adaptation to the COVID-19 crisis in the United States. *J Psychiatr Res* **2021**, *136*, 603-609, doi:10.1016/j.jpsychires.2020.10.035.
9. Davide, P.; Andrea, P.; Martina, O.; Andrea, E.; Davide, D.; Mario, A. The impact of the COVID-19 pandemic on patients with OCD: Effects of contamination symptoms and remission state before the quarantine in a preliminary naturalistic study. *Psychiatry Res* **2020**, *291*, 113213, doi:10.1016/j.psychres.2020.113213.
10. Donati, M.A.; Cabrini, S.; Capitanucci, D.; Primi, C.; Smaniotto, R.; Avanzi, M.; Quadrelli, E.; Bielli, G.; Casini, A.; Roaro, A. Being a gambler during the COVID-19 pandemic: A study with Italian patients and the effects of reduced exposition. *Int J Environ Res Public Health* **2021**, *18*, doi:10.3390/ijerph18020424.
11. Gaume, J.; Schmutz, E.; Daeppen, J.B.; Zobel, F. Evolution of the illegal substances market and substance users' social situation and health during the COVID-19 pandemic. *Int J Environ Res Public Health* **2021**, *18*, doi:10.3390/ijerph18094960.
12. Giel, K.E.; Schurr, M.; Zipfel, S.; Junne, F.; Schag, K. Eating behaviour and symptom trajectories in patients with a history of binge eating disorder during COVID-19 pandemic. *Eur Eat Disord Rev* **2021**, *29*, 657-662, doi:10.1002/erv.2837.
13. Goldfarb, Y.; Gal, E.; Golan, O. I Implications of employment changes caused by COVID-19 on mental health and work-related pychological need satisfaction of Autistic employees: A mixed-methods longitudinal study. *J Autism Dev Disord* **2022**, *52*, 89-102, doi:10.1007/s10803-021-04902-3.
14. Hamm, M.E.; Brown, P.J.; Karp, J.F.; Lenard, E.; Cameron, F.; Dawdani, A.; Lavretsky, H.; Miller, J.P.; Mulsant, B.H.; Pham, V.T.; et al. xperiences of American older adults with pre-existing depression during the beginnings of the COVID-19 pandemic: A multicity, mixed-methods study. *Am J Geriatr Psychiatry* **2020**, *28*, 924-932, doi:10.1016/j.jagp.2020.06.013.
15. Hennigan, K.; McGovern, M.; Plunkett, R.; Costello, S.; McDonald, C.; Hallahan, B. A longitudinal evaluation of the impact of the COVID-19 pandemic on patients with pre-existing anxiety disorders. *Ir J Psychol Med* **2021**, *38*, 258-265, doi:10.1017/ipm.2021.32.
16. Hochstatter, K.R.; Akhtar, W.Z.; Dietz, S.; Pe-Romashko, K.; Gustafson, D.H.; Shah, D.V.; Krechel, S.; Liebert, C.; Miller, R.; El-Bassel, N.; et al. Potential influences of the COVID-19 pandemic on drug use and HIV care among people living with HIV and substance use disorders: experience from a pilot mHealth intervention. *AIDS Behav* **2021**, *25*, 354-359, doi:10.1007/s10461-020-02976-1.
17. Johnco, C.J.; Chen, J.T.H.; Muir, C.; Strutt, P.; Dawes, P.; Siette, J.; Dias, C.B.; Hillebrandt, H.; Maurice, O.; Wuthrich, V.M. Long-term relapse rates after cognitive behaviour therapy for anxiety and depressive disorders among older adults: A follow-up study during COVID-19. *Australas J Ageing* **2021**, *40*, 208-212, doi:10.1111/ajag.12928.
18. Khosravani, V.; Aardema, F.; Samimi Ardestani, S.M.; Sharifi Bastan, F. The impact of the coronavirus pandemic on specific symptom dimensions and severity in OCD: A comparison before and during COVID-19 in the context of stress responses. *J Obsessive Compuls Relat Disord* **2021**, *29*, 100626, doi:10.1016/j.jocrd.2021.100626.
19. Kott, A.; Daniel, D.G. P.508 COVID-19 impact on entry symptom severity in schizophrenia clinical trials – preliminary data. *European Neuropsychopharmacology* **2020**, *40*, S286-S287, doi:10.1016/j.euroneuro.2020.09.372.
20. Leenaerts, N.; Vaessen, T.; Ceccarini, J.; Vrieze, E. How COVID-19 lockdown measures could impact patients with bulimia nervosa: Exploratory results from an ongoing experience sampling method study. *Eat Behav* **2021**, *41*, 101505, doi:10.1016/j.eatbeh.2021.101505.
21. Liu, X.; Jin, X.; Zhang, Y.; Zhang, L.; Li, Y.; Ma, J. Effect of coronavirus disease 2019 on the psychology and behavior of patients on methadone maintenance treatment in Wuhan, China: A clinical observational study. *Front Psychiatry* **2021**, *12*, 653662, doi:10.3389/fpsyt.2021.653662.
22. Lugo-Marín, J.; Gisbert-Gustemps, L.; Setien-Ramos, I.; Español-Martín, G.; Ibañez-Jimenez, P.; Forner-Puntonet, M.; Arteaga-Henríquez, G.; Soriano-Día, A.; Duque-Yemail, J.D.; Ramos-Quiroga, J.A. COVID-19 pandemic effects in people with Autism Spectrum Disorder and their caregivers: Evaluation of social distancing and lockdown impact on mental health and general status. *Res Autism Spectr Disord* **2021**, *83*, 101757, doi:10.1016/j.rasd.2021.101757.
23. Ma, J.; Hua, T.; Zeng, K.; Zhong, B.; Wang, G.; Liu, X. Influence of social isolation caused by coronavirus disease 2019 (COVID-19) on the psychological characteristics of hospitalized schizophrenia patients: a case-control study. *Translational Psychiatry* **2020**, *10*, 411, doi:10.1038/s41398-020-01098-5.
24. Ma, J.; Jiang, T.; Huang, H.; Li, R.; Zhang, L.; Liu, L.; Liu, X. Mental symptoms and stress of hospitalized schizophrenia patients with 2019 novel coronavirus disease: An observation study. *Front Psychiatry* **2021**, *12*, 557611, doi:10.3389/fpsyt.2021.557611.
25. Machado, P.P.P.; Pinto-Bastos, A.; Ramos, R.; Rodrigues, T.F.; Louro, E.; Gonçalves, S.; Brandão, I.; Vaz, A. Impact of COVID-19 lockdown measures on a cohort of eating disorders patients. *J Eat Disord* **2020**, *8*, 57, doi:10.1186/s40337-020-00340-1.
26. Matsunaga, H.; Mukai, K.; Yamanishi, K. Acute impact of COVID-19 pandemic on phenomenological features in fully or partially remitted patients with obsessive-compulsive disorder. *Psychiatry Clin Neurosci* **2020**, *74*, 565-566, doi:10.1111/pcn.13119.
27. Mergel, E.; Schützwohl, M. A longitudinal study on the COVID-19 pandemic and its divergent effects on social participation and mental health across different study groups with and without mental disorders. *Soc Psychiatry Psychiatr Epidemiol* **2021**, *56*, 1459-1468, doi:10.1007/s00127-021-02025-9.
28. Nisticò, V.; Bertelli, S.; Tedesco, R.; Anselmetti, S.; Priori, A.; Gambini, O.; Demartini, B. The psychological impact of COVID-19-related lockdown measures among a sample of Italian patients with eating disorders: a preliminary longitudinal study. *Eat Weight Disord* **2021**, *26*, 2771-2777, doi:10.1007/s40519-021-01137-0.
29. Orhan, M.; Korten, N.; Paans, N.; de Walle, B.; Kupka, R.; van Oppen, P.; Kok, A.; Sonnenberg, C.; Schouws, S.; Dols, A. Psychiatric symptoms during the COVID-19 outbreak in older adults with bipolar disorder. *Int J Geriatr Psychiatry* **2021**, *36*, 892-900, doi:10.1002/gps.5489.
30. Pan, K.Y.; Kok, A.A.L.; Eikelenboom, M.; Horsfall, M.; Jörg, F.; Luteijn, R.A.; Rhebergen, D.; Oppen, P.V.; Giltay, E.J.; Penninx, B. The mental health impact of the COVID-19 pandemic on people with and without depressive, anxiety, or obsessive-compulsive disorders: a longitudinal study of three Dutch case-control cohorts. *Lancet Psychiatry* **2021**, *8*, 121-129, doi:10.1016/s2215-0366(20)30491-0.
31. Peckham, E.; Allgar, V.; Crosland, S.; Heron, P.; Johnston, G.; Newbronner, E.; Ratschen, E.; Spanakis, P.; Wadman, R.; Walker, L.; et al. Investigating smoking and nicotine dependence among people with severe mental illness during the COVID-19 pandemic: analysis of linked data from a UK Closing the Gap cohort. *BJPsych Open* **2021**, *7*, e86, doi:10.1192/bjo.2021.45.
32. Pinkham, A.E.; Ackerman, R.A.; Depp, C.A.; Harvey, P.D.; Moore, R.C. A longitudinal investigation of the effects of the COVID-19 pandemic on the mental health of individuals with pre-existing severe mental illnesses. *Psychiatry Res* **2020**, *294*, 113493, doi:10.1016/j.psychres.2020.113493.
33. Riblet, N.B.; Stevens, S.P.; Shiner, B.; Cornelius, S.; Forehand, J.; Scott, R.C.; Watts, B.V. Longitudinal examination of COVID-19 public health measures on mental health for rural patients with serious mental illness. *Mil Med* **2021**, *186*, e956-e961, doi:10.1093/milmed/usaa559.
34. Rutherford, B.R.; Choi, C.J.; Chrisanthopolous, M.; Salzman, C.; Zhu, C.; Montes-Garcia, C.; Liu, Y.; Brown, P.J.; Yehuda, R.; Flory, J.; et al. The COVID-19 pandemic as a traumatic stressor: Mental health responses of older adults with chronic PTSD. *Am J Geriatr Psychiatry* **2021**, *29*, 105-114, doi:10.1016/j.jagp.2020.10.010.
35. Seethaler, M.; Just, S.; Stötzner, P.; Bermpohl, F.; Brandl, E.J. Psychosocial Impact of COVID-19 pandemic in elderly psychiatric patients: a longitudinal study. *Psychiatr Q* **2021**, *92*, 1439-1457, doi:10.1007/s11126-021-09917-8.
36. Seitz, K.I.; Bertsch, K.; Herpertz, S.C. A prospective study of mental health during the COVID-19 pandemic in childhood trauma-exposed individuals: Social support matters. *J Trauma Stress* **2021**, *34*, 477-486, doi:10.1002/jts.22660.
37. Sharma, L.P.; Balachander, S.; Thamby, A.; Bhattacharya, M.; Kishore, C.; Shanbhag, V.; Sekharan, J.T.; Narayanaswamy, J.C.; Arumugham, S.S.; Reddy, J.Y.C. Impact of the COVID-19 pandemic on the short-term course of obsessive-compulsive disorder. *J Nerv Ment Dis* **2021**, *209*, 256-264, doi:10.1097/nmd.0000000000001318.
38. Strauss, G.P.; Macdonald, K.I.; Ruiz, I.; Raugh, I.M.; Bartolomeo, L.A.; James, S.H. The impact of the COVID-19 pandemic on negative symptoms in individuals at clinical high-risk for psychosis and outpatients with chronic schizophrenia. *Eur Arch Psychiatry Clin Neurosci* **2022**, *272*, 17-27, doi:10.1007/s00406-021-01260-0.
39. Wynn, J.K.; McCleery, A.; Novacek, D.; Reavis, E.A.; Tsai, J.; Green, M.F. Clinical and functional effects of the COVID-19 pandemic and social distancing on vulnerable veterans with psychosis or recent homelessness. *J Psychiatr Res* **2021**, *138*, 42-49, doi:10.1016/j.jpsychires.2021.03.051.
40. Yocum, A.K.; Zhai, Y.; McInnis, M.G.; Han, P. Covid-19 pandemic and lockdown impacts: A description in a longitudinal study of bipolar disorder. *J Affect Disord* **2021**, *282*, 1226-1233, doi:10.1016/j.jad.2021.01.028.
